# Thiazole Ring—A Biologically Active Scaffold

**DOI:** 10.3390/molecules26113166

**Published:** 2021-05-25

**Authors:** Anthi Petrou, Maria Fesatidou, Athina Geronikaki

**Affiliations:** School of Pharmacy, Aristotle University of Thessaloniki, 54124 Thessaloniki, Greece; anthi.petrou.thessaloniki1@gmail.com (A.P.); fesa.maria@gmail.com (M.F.)

**Keywords:** thiazoles, antimicrobial, neglected, anticonvulsant, anti-inflammatory, carbonic anhydise, antiviral, HIV, tuberculosis, antioxidant

## Abstract

Background: Thiazole is a good pharmacophore nucleus due to its various pharmaceutical applications. Its derivatives have a wide range of biological activities such as antioxidant, analgesic, and antimicrobial including antibacterial, antifungal, antimalarial, anticancer, antiallergic, antihypertensive, anti-inflammatory, and antipsychotic. Indeed, the thiazole scaffold is contained in more than 18 FDA-approved drugs as well as in numerous experimental drugs. Objective: To summarize recent literature on the biological activities of thiazole ring-containing compounds Methods: A literature survey regarding the topics from the year 2015 up to now was carried out. Older publications were not included, since they were previously analyzed in available peer reviews. Results: Nearly 124 research articles were found, critically analyzed, and arranged regarding the synthesis and biological activities of thiazoles derivatives in the last 5 years.

## 1. Introduction

One of the main objectives of organic and medicinal chemistry is the design, synthesis, and development of molecules having therapeutic potential as human drugs. During the past decade, combinatorial chemistry has provided access to chemical libraries based on privileged structures [1], with heterocyclic scaffolds receiving special attention as they belong to a class of compounds with proven utility in medicinal chemistry [2]. There are numerous biologically active molecules with five-membered rings, containing two hetero atoms. The thiazole ring is one of them. 

**Figure 1:**
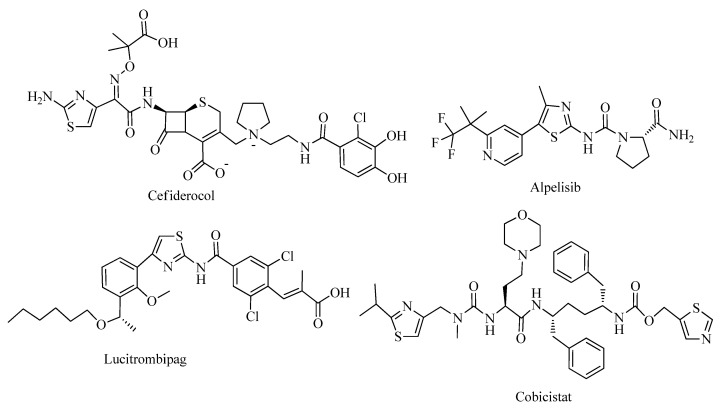
Structures of thiazole-bearing drugs recently approved by the FDA.

Thiazole is a good pharmacophore nucleus due to its various pharmaceutical applications. Its derivatives have wide range of biological activities such as antioxidant, analgesic, antibacterial, anticancer, antiallergic, antihypertensive, anti-inflammatory, antimalarial, antifungal, and antipsychotic [3,4,5,6,7,8]. The thiazole scaffold is present in more than 18 FDA-approved drugs. Among them are cefiderocol, which was the first siderophore antibiotic approved by the FDA in 2019 under the brand name Fetroja^®^ (Figure 1). This thiazole derivative was found to be active against a wide range of multi-drug resistant Gram-negative bacteria, including *Pseudomonas aeruginosa* (*P.aeruginosa*) and used to treat complicated urinary tract infections in case when no other treatment is available [9,10,11]. Another thiazole-based drug is alpelisib with the brand name Pigray^®^, which was approved again in 2019 for the treatment of certain types of breast cancer. Worldwide, breast cancer is one of the most common severe diseases and the second leading cause of cancer death mostly in less developed countries [12]. Lusutrombopag is a medication that was approved in 2018 for the stimulation of platelet formation used to treat thrombocytopenia such as thrombocytopenia associated with chronic liver disease. Another example is cobicistat, which is a drug used for the treatment of human immunodeficiency virus infection (HIV) as a drug prolonging the half-life of some antivirotic drugs. It was approved by the FDA in 2018 [13].

The goal of the present review is to highlight the recent advancement in the discovery of biologically active thiazole derivatives. This review covers a short time from 2015 until now since several reviews have been published.

## 2. Thiazole Derivatives

### 2.1. Chemistry of Thiazole Derivatives

**Scheme 1:**
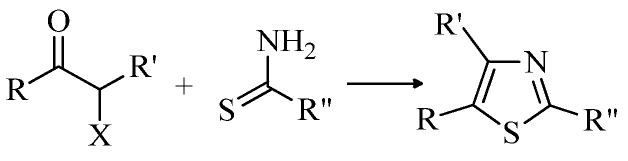
Hantzsch synthesis of thiazoles.

There are many methods for synthesizing thiazole and its derivatives. Some are presented below: Synthesis according to Hantzsch (1889) is the main mode of synthesis of thiazole derivatives and refers to the reaction of α-halocarbonyl compounds with thioamides or thiourea [14] (Scheme 1).

**Scheme 2:**
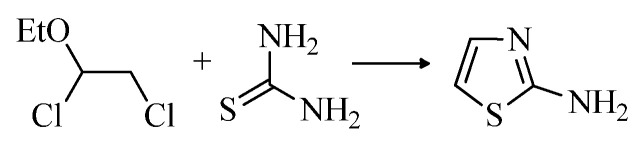
Synthesis of 2-aminothiazole from thiourea and 1,2-dichloro-1-ethoxyethane.

The reaction mechanism consists of the nucleophilic attack of the thioamide sulfur atom on the alpha carbon of the alpha-halocarbonyl, with the formation of an intermediate by subsequent dehydration to the corresponding thiazole. A variation of the foregoing synthesis comprises concentrating thiourea or its derivatives with 1,2-dichloro-1-ethoxyethane [15,16] (Scheme 2).

**Scheme 3:**
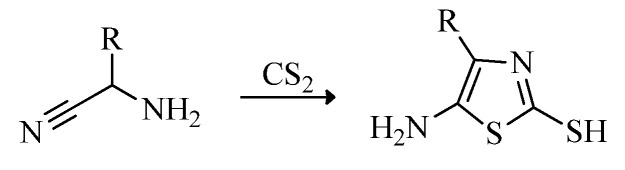
Cook–Heilbron thiazole synthesis.

Another way to synthesize thiazole derivatives is the Cook–Heilbron method, wherein an aminonitrile reacts with carbon disulfide [17]. According to this method, 2,4-disubstituted 5-aminothiazole derivatives are synthesized (Scheme 3).

**Scheme 4:**
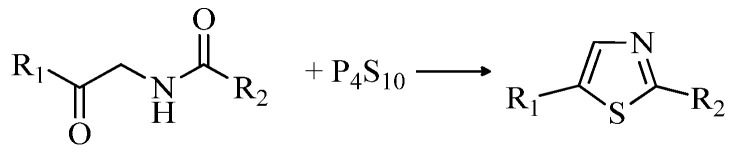
Robinson–Gabriel thiazole synthesis.

Thiazole derivatives can be synthesized by the Robinson–Gabriel method based on the cyclization of acylaminocarbonyl compounds in the presence of stoichiometric amounts of phosphorus pentasulfide [18] (Scheme 4).

**Scheme 5:**
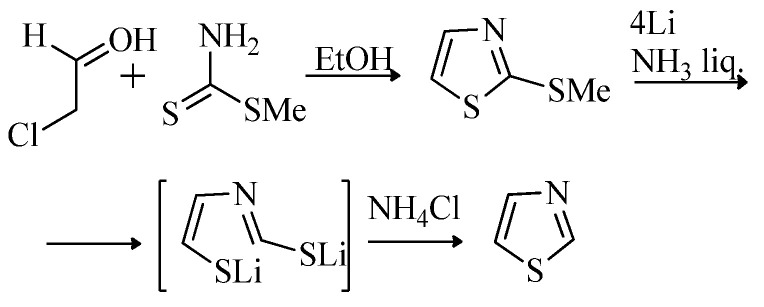
Synthesis of thiazole by reduction of methylthiothiazole.

An interesting synthesis of thiazole is mediated by reduction of 2-methylthiothiazole [19] (Scheme 5).

**Scheme 6:**
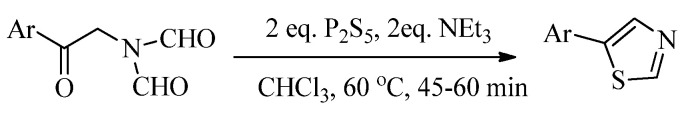
Synthesis of 5-arylthiazoles.

Additionally, there are novel recent methods for the synthesis of thiazole derivatives. Sheldrake et al. [20] reported the synthesis of 5-arylthiazoles by treatment of *N,N*-diformylaminomethyl aryl ketones with phosphorus pentasulfide and triethylamine in chloroform. The reaction results in 5-arylthiazoles with good yields (Scheme 6).

**Scheme 7:**
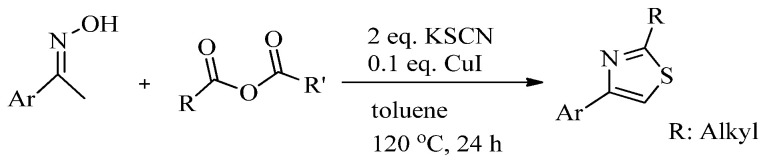
Synthesis of thiazoles from oximes, anhydrides, and KSC.

Tang et al. [21] reported the synthesis of 5-arylthiazoles by a copper-catalyzed [3+1+1]-type condensation of oximes, anhydrides, and potassiumthiocyanate (KSCN (Scheme 7).

**Scheme 8:**
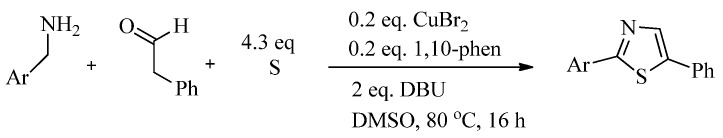
Synthesis of thiazoles from aldehydes, amines, and elemental sulfur.

Wang et al. [22] synthesized thiazoles from simple aldehydes, amines, and sulfur in the presence of molecular oxygen as a green oxidant by a practical Cu-catalyzed oxidative, multiple Csp^3^-H bond cleavage process (Scheme 8).

**Scheme 9:**
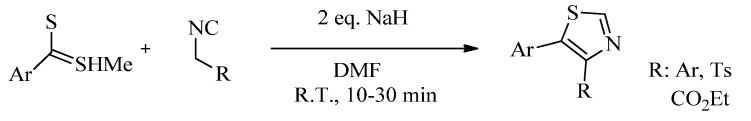
Synthesis of thiazoles by cyclization of isocyanide with methyl arene- and hetarenecarbodithioatesa.

Lingaraju et al. [23] synthesized 4,5-disubstituted thiazoles via the base-induced cyclization of active methylene isocyanides such as tosylmethyl isocyanide, ethyl isocyanoacetate, and arylmethyl isocyanides with methyl arene- and hetarenecarbodithioates (Scheme 9).

**Scheme 10:**
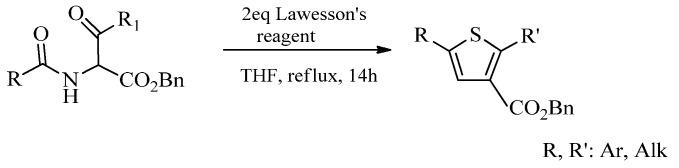
Synthesis of thiazoles from vinyl azides and potassium thiocyanate under different reaction conditions.

Sanz-Cervera [24] synthesized a small library of compounds with thiazole scaffolds and structural diversity in both positions 2 and 5. Double acylation of a protected glycine affords intermediate α-amido-β-ketoesters, which in turn reacting with Lawesson’s reagent furnished 1,3-thiazoles (Scheme 10).

**Scheme 11:**
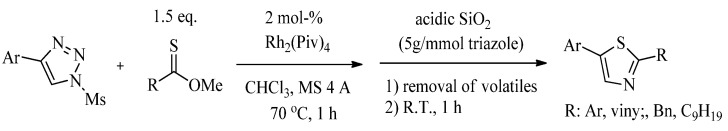
Synthesis of thiazoles from sulfonyl-1,2,3 –triazoles and thionoesters.

Miura et al. [25] reported the synthesis of 2,5-disubstituted thiazoles from 1-sulfonyl-1,2,3-triazoles and thionoesters using rhodium (II) catalyst (Scheme 11).

**Scheme 12:**
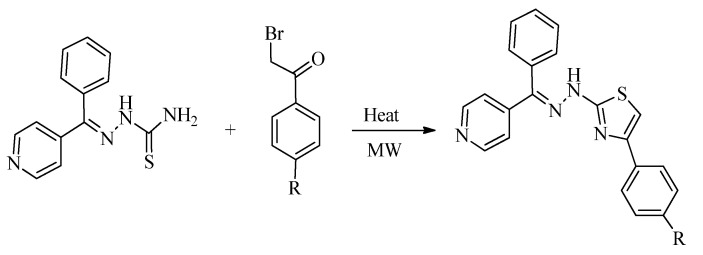
Synthesis of thiazoles from thiosemicarbazones and α-bromoketones under microwave irradiation.

Some thiazoles derivatives were synthesized by microwave irradiation [26,27]. Chinnaraja and Rajalakshmi [28] synthesized novel hydrazinyl thiazole derivatives with good yield and purity by this method (Scheme 12).

**Scheme 13:**
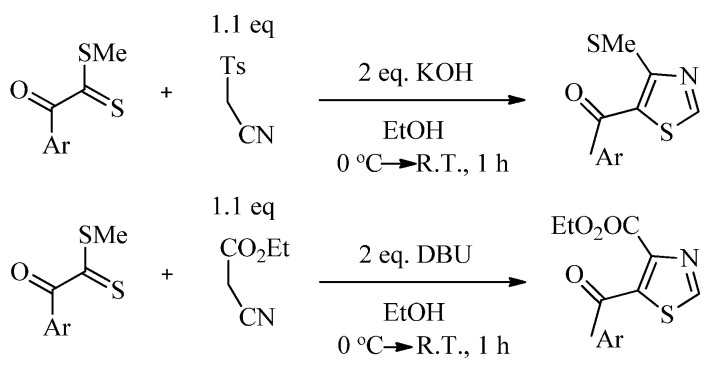
Synthesis of substituted thiazoles via cyclization of tosylmethyl isocyanide with α-oxodithioesters.

Recently, Kiran et al. [29] synthesized 4-methylthio-5-acylthiazoles and 4-ethoxycarbonyl-5-acylthiazoles via a cyclization of tosylmethyl isocyanide with α-oxodithioesters in the presence of KOH and ethyl isocyanoacetate with α-oxodithioesters in the presence of DBU/EtOH, respectively (Scheme 13).

**Scheme 14:**
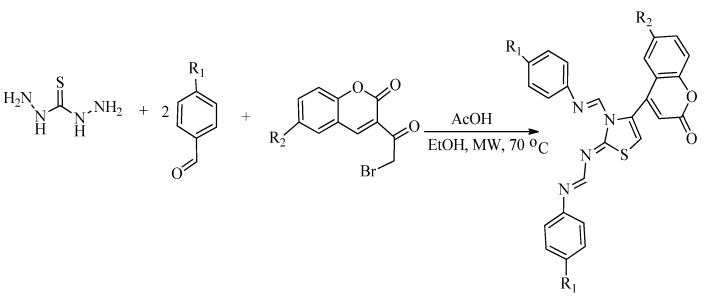
Synthesis of new coumarin-based thiazole derivatives, by reaction of thiocarbohydrazide, aldehydes, and α-halocarbonyl coumarins.

Mamidala et al. [30] reported the synthesis of new coumarin-based thiazole derivatives, by reaction of thiocarbohydrazide, aldehydes, and α-halocarbonyl coumarins in a molar ratio of 1:2:1, under microwave heating (Scheme 14). Different solvents were used. Ethanol with a catalytic amount of acetic acid gave the highest yield (88–93%) after a short time (5–8 min) and mild conditions. 

### 2.2. Synthesis of 2-Aminothiazoles

**Scheme 15:**
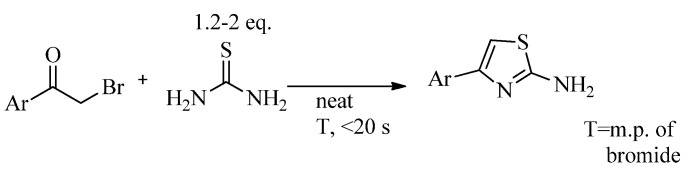
Synthesis of 2-aminothiazole by condensation of 2-bromoacetophenones with thiourea.

Facchinetti et al. [31] reported the Hantzsch condensation of 2-bromoacetophenones with thiourea or selenourea as a simple, fast, and eco-friendly solvent-free synthesis of 2-aminothiazoles and 2-amino-1,3-selenazoles without the use of a catalyst. This reaction is accomplished within a few seconds with good yields (Scheme 15).

**Scheme 16:**
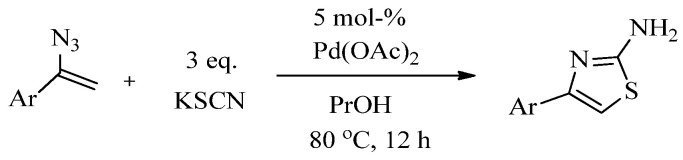
Synthesis of 4-substituted 2-aminothiazoles from vinyl azides and potassium thiocyanate.

Chen et al. [32] synthesized 4-substituted 2-aminothiazoles from vinyl azides and potassium thiocyanate, using palladium (II) acetate as a catalyst, while 4-substituted 5-thiocyano-2-aminothiazoles are prepared by using ferric bromide (Scheme 16).

**Scheme 17:**
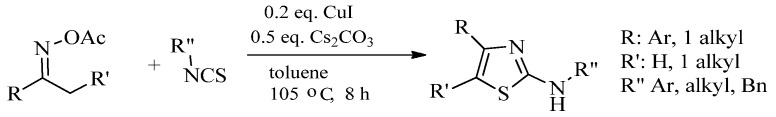
Synthesis of 4-substituted and 4,5-disubstituted 2-aminothiazoles through a copper-catalyzed coupling of oxime acetates with isothiocyanates.

Tang et al. [33] used a copper-catalyzed coupling of oxime acetates with isothiocyanates in order to synthesize various 4-substituted and 4,5-disubstituted 2-aminothiazoles (Scheme 17).

**Scheme 18:**
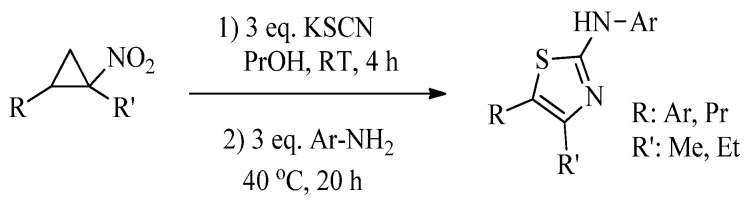
Synthesis of polysubstituted 2-aminothiazoles.

Another way for the synthesis of polysubstituted 2-aminothiazoles is a one-pot three-component reaction of α-nitro epoxides, potassium thiocyanate, and primary amines reported by Zhu [34]. This method is highly efficient and yields are good (Scheme 18).

**Scheme 19:**
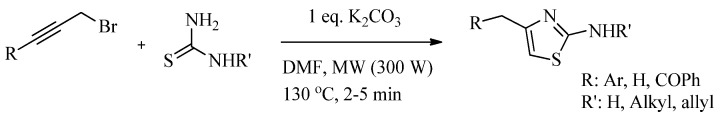
Synthesis of 2-aminothiazoles via a domino alkylation-cyclization reaction of propargyl bromide with thiourea.

Castagnolo et al. [35] synthesized 2-aminothiazoles and 5*H*-thiazolo [3,2-*a*]pyrimidin-5-ones via a domino alkylation-cyclization reaction of propargyl bromides with thioureas and thiopyrimidinones under microwave irradiation in a very short time and with good yields (Scheme 19).

**Scheme 20:**
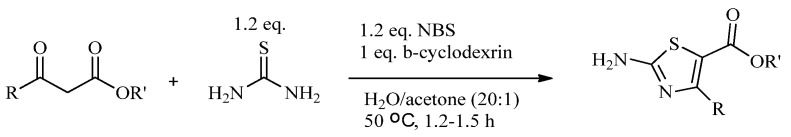
Synthesis of thiazole derivatives by α-halogenation of β-keto esters, followed by cyclization with thiourea.

Narender et al. [36] reported the synthesis of 2-amino-4-alkyl- and 2-amino-4-arylthiazole-5-carboxylates by α-halogenation of β-keto esters with *N*-bromosuccinimide, which was followed by cyclization with thiourea (Scheme 20).

**Scheme 21:**
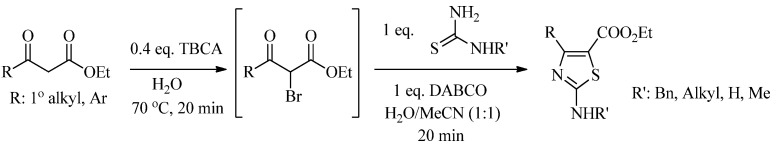
Synthesis of 2-aminothiazoles from β-keto ester, tribromoisocyanuric acid, and thiourea.

De Andrade et al. [37] reported a one-pot protocol for the synthesis of 2-aminothiazoles using readily available β-keto esters and tribromoisocyanuric acid via α-monohalogenation in aqueous medium and subsequent reaction with thiourea and DABCO (Scheme 21).

**Scheme 22:**
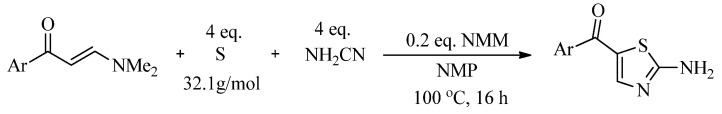
Synthesis of 2-amino-5-acylthiazoles by a one-pot three-component cascade.

Fu et al. [38] reported the synthesis of 2-amino-5-acylthiazoles by a one-pot three-component cascade cyclization of enaminones, cyanamide, and elemental sulfur (Scheme 22).

## 3. Biological Activities of Thiazoles

### 3.1. Thiazole Derivatives as Antimicrobial Agents

Currently, medicinal chemists pay serious attention to the design and development of antimicrobial agents with different modes of action due to the growth of the bacterial resistance accentuated by appearance of multidrug-resistant strains such as *Staphylococcus aureus*, *Enterococcus* sp., *Acinetobacter baumannii (A. baumani)*, *Pseudomonas aeruginosa (P. aeruginosa)*, *Enterobacter cloeacae (E. cloacae)* [39] and *Candida* sp. with acquired resistance to fluconazole [40], playing roles in many human infections (e.g., pulmonary and urinary).

The development of novel antimicrobial drugs was quite poor for some decades, and this precipitated in a lack of highly active drugs to resistant Gram-negative bacteria. Hence, the need for the discovery of novel agents targeting both sensitive and resistant strains [41] with different and rather novel modes of action is obvious.

**Figure 2:**
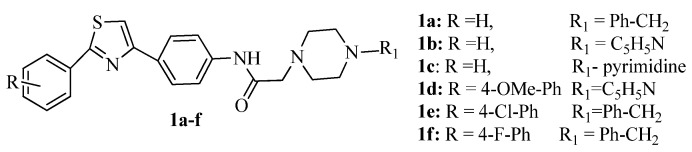
Structure of N-(4-(2-phenylthiazol-4-yl)phenyl)-2-(piperazin-1-yl)acetamide derivatives.

Yurttas et al. [42] reported the synthesis and evaluation of antimicrobial activity of sixteen 2-(4-arylpiperazine-1-yl)-*N*-[4-(2-(4-substituted phenyl)thiazol-4-yl)phenyl] acetamide derivatives (**1a**–**1p**) (Figure 2). Compounds were tested against a panel of Gram-positive, Gram-negative bacteria and fungi, using as reference compounds chloramphenicol and ketoconazole, respectively. In general, the antibacterial activity of these compounds was much lower (MIC at 100–400 μg/mL) compared to reference drug (MIC 25–50 μg/mL). Only two compounds **1b**, **1c** showed a slightly better activity against Gram-positive *E faecalis* with a MIC of 100 μg/mL. 

The antifungal activity of the compounds was more pronounced than their antimicrobial activity. Compounds **1a**, **1b**, **1e**, and **1f** were found to be equipotent with ketoconazole (MIC 50 μg/mL) against *Candida albicans* (*C. albicans*), while compound **1d** was found to be equipotent against *Candida glabrata (C. glabrata).* Furthermore, compounds **1a** and **1b** were also equipotent with the reference drug against *Candida krusei* (*C. krusei*) and *Candida parapsilosis* (*C. parapsilosis*), respectively. 

**Figure 3:**
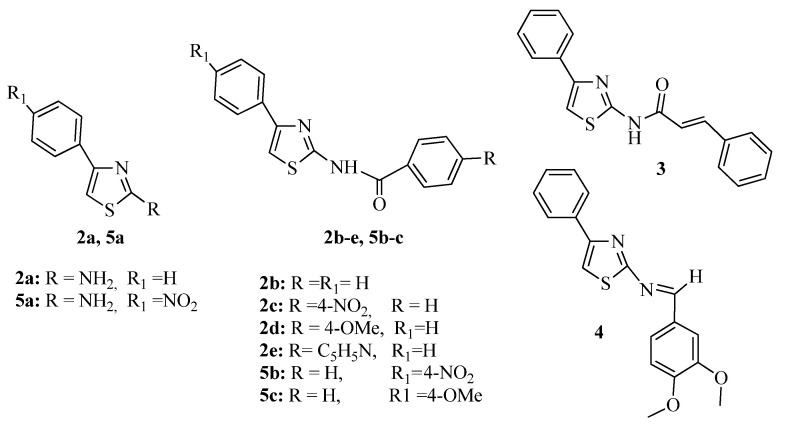
Structure of 2, 4-disubstituted thiazole derivatives.

Arora et al. [43] synthesized novel 2, 4-disubstituted thiazole derivatives (**2a**–**d**, **3**, **4**, **5a**–**5c**) (Figure 3) and evaluated their antimicrobial activity against two Gram-positive (*S. aureus*, *B. subtilis*), one Gram-negative (*E. coli*) bacteria and two fungal species (*C. albicans* and *A. niger*) by the tube dilution method [44]. The evaluation revealed that compounds **2c**, **4**, **5b**, and **5c** were found to be the most potent among all compounds tested against *B. subtilis* with MIC values of 4.51–4.60 mM. The same activity was achieved for compounds **2c**, **4**, and **5c** against *S. aureus.*

These four compounds **2c**, **4**, **5b**, and **5c** appeared to be active against *C. albicans* (pMICca = 3.92–4.01 mM), while compounds **5b** and **5c** exhibited also good activity against *Aspergilus niger (A. niger)* with MIC values of 4.01–4.23 mM. Their effects were clearly lower than those of the reference drugs ciprofloxacin and fluconazole. Structure–activity relationship analysis revealed that the presence of electron-withdrawing NO_2_ (**2c**, **5b**, **5c**) and electron-donating OMe (**2d**, **4**, **5c**) groups in the para position of benzene ring at thiazole moiety is beneficial for the activity. An increase in lipophilicity by replacement of the NH_2_ group (**2a** and **5a**) with benzamide (**2b**–**2d** and **5b**, **5c**), amide (**2e**, **3**), and 3,4-dimethoxybenzylidene (**4**) had a positive effect on the antimicrobial activity as well.

According to docking results, compounds (**2a**–**d**, **3**, **4**, **5a**–**5c**) are able to bind to glucosamine-6-phosphate synthase (GlcN-6-P synthase) by binding with one or the other amino acids in the active pockets.

Lączkowski et al. [45] presented the synthesis of novel imidazolythiazoles **6a**–**6i** and investigated their antimicrobial activity against twelve bacterial and fungal species using the broth microdilution method [44] and ciprofloxacin/fluconazole as reference drugs, respectively.

**Figure 4:**
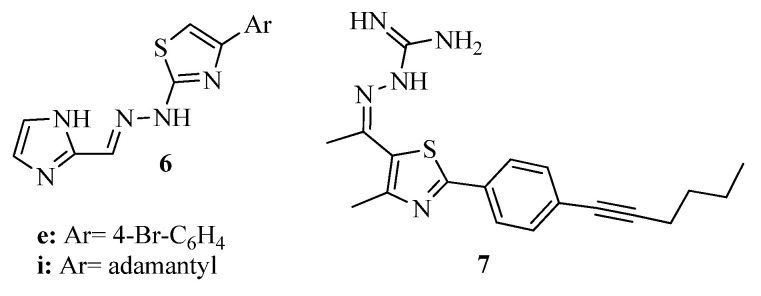
Structures of imidazolothiazole (**6**) and 2,5-disubstituted thiazole derivatives (**7**).

Compounds **6**e and **6i** (Figure 4) were found to be the most active among all tested against some species. They exhibited a good activity against *Micrococcus luteus* ATCC 10240 and *Bacillus* spp with MIC at 1.95–3.91 μg/mL and 3.91–15.62 μg/mL, respectively. The MBC was in range of 7.81–125 μg/mL. These compounds were also potent against *Streptococcus* spp. with MIC/MBC at 7.81–15.62 μg/mL and 31.25–500 μg/mL.

Mohammad et al. [46], based on their precious study [47] identified a novel lead thiazole compound with potent antimicrobial activity against clinically relevant isolates of MRSA. These synthesized series of 2,5-disubstituted thiazole derivatives were evaluated for their antimicrobial activity against MRSA, VISA, and VRSA strains. The most potent compound against VISA and different VRSA strains was compound **7** (Figure 4) with MIC in a range of 0.7–2.8 μg/mL compared to vancomycin (MIC 0.7→190.2 μg/mL). The structure–activity relationship studies revealed that the presence of a nonpolar, hydrophobic moiety at position 2 of thiazole and the ethylidenehydrazine-1-carboximidamide head group at position 5 are beneficial for the antibacterial activity of these compounds.

**Figure 5:**
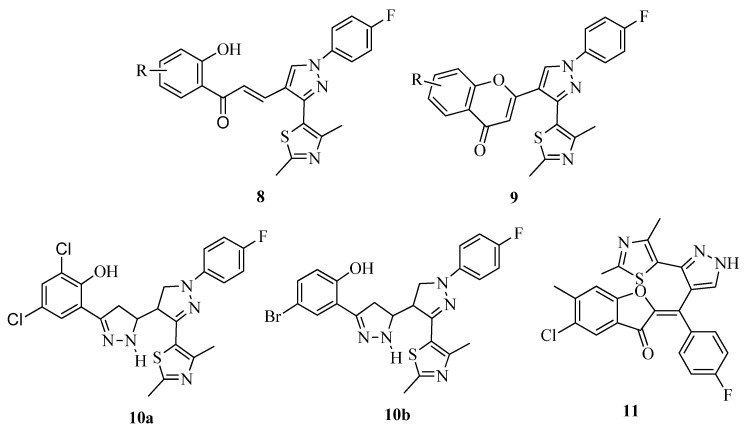
Structures of derivatives of 1-(2-hydroxyphenyl)-3-[3-(2,4-dimethyl-thiazol-5-yl)-1-(4-fluoro-phenyl)-1*H*-pyrazol-4-yl]-propenone (**8**), 2-[3-(2,4-dimethyl-1,3-thiazol-5-yl)-1-(4-fluorophenyl)-1*H*-pyrazol-4-yl]-4*H*-chromen-(**9**), 2-[3′-(2,4-dimethyl-thiazol-5-yl)-1′-(4-florophenyl)-3,4-dihydro-2*H*,1′*H*-[3,4′]bipyrazolyl-5-yl]-phenol (**10**), 2-[3-(2,4-dimethylthiazol-5-yl)-1-(4-fluorophenyl)-1*H*-pyrazol-4-ylmethylene]-benzofuran-3-one.

Karale et al. [48] reported the synthesis of twenty-four compounds, derivatives of 1-(2-hydroxyphenyl)-3-[3-(2,4-dimethyl-thiazol-5-yl)-1-(4-fluoro-phenyl)-1*H*-pyrazol-4-yl]-propenone (**8**), 2-[3-(2,4-dimethyl-1,3-thiazol-5-yl)-1-(4-fluorophenyl)-1*H*-pyrazol-4-yl]-4*H*-chromen-(**9**), 2-[3′-(2,4-dimethyl-thiazol-5-yl)-1′-(4-florophenyl)-3,4-dihydro-2*H*, 1′*H*-[3,4′]bipyrazolyl-5-yl]-phenol (**10**), 2-[3-(2,4-dimethylthiazol-5-yl)-1-(4-fluorophenyl)-1*H*-pyrazol-4-ylmethylene]-benzofuran-3-one (**11**) (Figure 5), and evaluated their antibacterial activity. Compounds were tested against bacterial strains *E. coli*, *Salmonella typhi*, *B. subtilis*, and *S. aureus* using the agar well diffusion method with ciprofloxacin as the reference drug. Compounds from groups **8** and **9** were inactive against all bacteria in concentration of 100 μg/mL, while groups of compounds **10** and **11** exhibited moderate to low activity with inhibition zone (IZ) 12-18 mm compared to the reference drug (IZ of 35–46 mm). Compounds **10a**–**b** and **11** showed the highest activity among compounds tested against *Bacilus subtilis (B.subtilis)*, (ZI 16, 18, and 16 mm, respectively). Compound **11** was also active against *Staphylococcus aureus (S. aureus)* (17 mm). 

**Figure 6:**
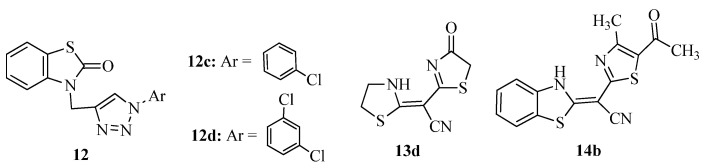
Structures-bromobenzo[d]thiazol-2(3H)-one derived 1,2,3-triazole derivatives (**12**) and (E)-2-(4-oxo-4,5-dihydrothiazol/dihydrobenzothiazol-2-yl)-2-(thiazolidin-2-ylidene) acetonitrile (**13**,**14**).

Nagavelli et al. [49] synthesized a series of new 6-bromobenzo[d]thiazole-2(3H)-one derived 1,2,3-triazole derivatives (**12a**–**j**) (Figure 6) with the aim to study their cytotoxicity and antibacterial activity as well. The synthesized compounds (**12a**–**j**) were tested for their in vitro antibacterial activity against both Gram-positive and Gram-negative bacteria such as *Streptococcus pyogenes (S. pyogenes), S. aureus, P. aeruginosa*, and *Klebsiella pneumonia (K. pneumoniae)* by the agar well diffusion method [50]. Compounds with 3-chlorophenyl and 3,5-dichloro phenyl groups on the triazole ring (**12c** and **12d**) (Figure 6) showed excellent inhibition (MIC 25 μg/mL) against *S. aureus*, being equipotent with streptomycin used as the reference drug. These compounds exhibited also good activity against *P. aeruginosa* with MIC at 25 μg/mL compared to streptomycin (MIC 1.25 μg/mL), while compound **12d** was also the most active among compounds tested against *S. pyogenes *(MIC 25 μg/mL) being twice less active than streptomycin. The rest of compounds showed moderate to low antibacterial activity.

Ghasemi et al. [51] reported the inhibitory effects of eight thiazole derivatives **13a**–**d** and **14a**–**d** (Figure 6), which have been assessed on three animal bacterial pathogens, such as *Rhodococcus equi* (*R. equi)* ATCC33701, *Pasteurella multocida (P. multocida)* ATCC 12948, and *Brucella abortus (B. abortus)* ATCC 23448. Only two derivatives **13d** and **14b** were active against *R. equi* and *P. multocida*, with MIC of 64 and 32 μg/mL, respectively.

**Figure 7:**
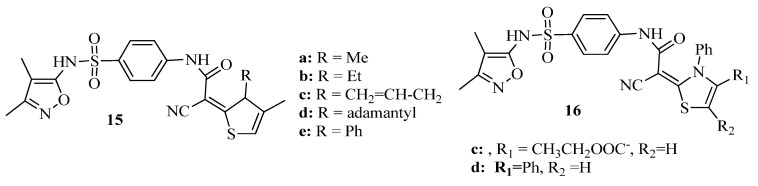
Structures of 2,3-dihydrothiazoles tagged with sulfisoxazole moiety.

Nasr et al. [52] presented the design and synthesis of new functionalized 2,3-dihydrothiazoles tagged with sulfisoxazole moiety (**15a**–**15e**, **16a**–**e**, Figure 7) and evaluated their antimicrobial activity against three Gram-positive and three Gram-negative bacteria as well as against two filamentous fungi and one fungus that is a member of the human microbiome (*Aspergillus fumigatus* (RCMB 02568), *Syncephalastrum racemosum* (RCMB 05922), and *Geotricum candidum* (RCMB 05097). Compounds **15a**–**e** displayed a good antibacterial activity against *S. pneumoniae, B. subtilis, S. epidermitis*, and *E. coli*, with compounds **15c** and **15d** (MIC 0.06 μg.ml) being four times more active than ampicillin (MIC 0.24 μg/mL) against *B. subtilis*. Moreover, compound **15c** displayed superior activity than ampicillin (MIC 0.12 μg/mL) against *S. pneumonia*, while **15d** was equipotent with ampicillin (0.12 μg/mL) and **15e** (MIC 19.95 μg/mL) was almost equipotent with gentamycin (MIC 19.9 μg/mL) but less active than sulfisoxazole (MIC 15.2 μg/mL). Compounds **15a**–**e** exhibited also higher activity against *P vulgaris* with MIC in a range of 13.0–19.8 μg/mL and *K. pneumonia* with MIC ranging from 17.9 to 24.8 μg/mL than gentamycin (MIC 23.4 μg/mL and 26.32 μg/mL respectively). Compounds **15a**, **b**, **d**, and **e** (MIC 13.6–20.3 μg/mL) were more potent than amphotericin B (MIC 23.7 μg/mL) against *A. fumigatus*, while **15a**–**15d** (MIC 11.7–18.8 μg/mL) were more potent than the reference drug (MIC 19.7 μg/mL) against *S. racemosum* and compounds **15a**–**15e** (MIC 11.5–26.9 μg/mL) exhibited higher activity than amphotericin B (MIC 28.7 μg/mL) against *Geotrichum candidum* (*G. candidum)*. *N*-Phenyl-thiazoles (**16a**–**e**) showed good to excellent activity against all bacteria tested with compound **16e** to be the most active except against *Proteus vulgaris (P. vulgaris)*, while **16b** was the most active against all fungal species tested.

The structure–activity relationship studies revealed that the larger the N-alkyl, the higher the activity: the reason is probably a higher lipophilicity. The docking studies demonstrated that compounds could occupy not only PABA but also pterin binding pockets of deoxyhypusine synthase (DHPS).

**Figure 8:**
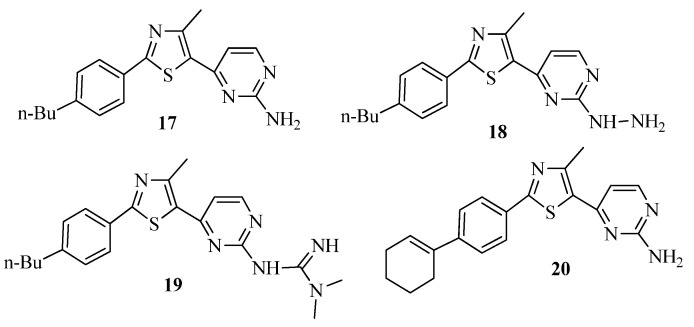
Structures of 2-(1-(2-(4-butylphenyl)-4-methylthiazol-5-yl)ethylidene)aminoguanidine analogues.

Mayhoub et al. [53] synthesized a series of second-generation analogues for 2-(1-(2-(4-butylphenyl)-4-methylthiazol-5-yl) ethylidene) aminoguanidine (**17**–**20**, Figure 8), which were tested against *MRSA*. The hydrolysable C=N bond was hidden inside a more metabolically stable pyrimidine nucleus in order to enhance the pharmacokinetic profile of compounds. Among the tested compounds, hydrazide and *N,N*-dimethylguanidine-containing derivatives **18** (MIC 0.4 μg/mL) and **19** (MIC 0.78 μg/mL) were found to be more potent than vancomycin, which was the drug of preference for treatment of systemic MRSA infections.

The replacement of butyl substituent (**17**) with MIC 3.12 ± 0.0 μg/mL by a more lipophilic cyclohexyl moiety led to compound **20**, which was 4-fold more active (MIC 0.78 μg/mL). Nevertheless, the compound was still 1-fold less active than vancomycin.

**Figure 9:**
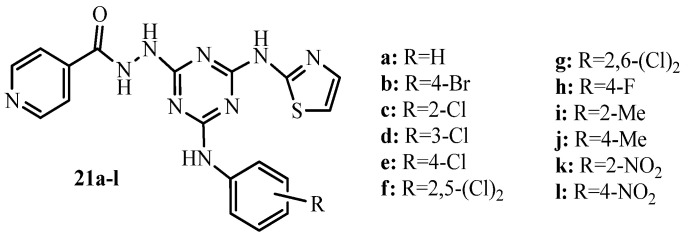
Structures of N-(4-(arylamino)-6-(thiazol-2-ylamino) 1,3,5-triazin-2-yl) isonicotinohydrazides.

Desai et al. [54] synthesized a series of N’-(4-(arylamino)-6-(thiazol-2-ylamino)1,3,5-triazin-2-yl)isonicotinohydrazides (**21a**–**l**, Figure 9) and studied their antibacterial activity against Gram-positive *S. aureus* (MTCC-96), *S. pyogenes* (MTCC-442), Gram-negative bacteria *E.coli* (MTCC-443), *P.aeruginosa* (MTCC-1688), and fungi (*C. albicans, A. niger*, and *Aspergillus clavatus*) strains using ciprofloxacin and griseofulvin as reference drugs.

All compounds showed varying degrees of inhibition against the different bacterial species. The highest activity against all species was observed for compounds **21k** and **21l**. They were equipotent or even more potent than ciprofloxacin against *E. coli* (MIC 25 μg/mL), *P. aeruginosa* (MIC 25 μg/mL), *S. aureus*, and *S pyogenes* (MIC 50 μg/mL). Against *C. albicans* and *A. niger*, most compounds were at least equipotent compared to the standard drug griseofulvin. Thus, compounds **21a**, **21c**, **21i**, and **21j** showed the same activity as griseofulvin (MIC 500 μg/mL) against *C. albicans*, while compounds **21d**, **21e**, **21g**, **21h** (MIC 100 μg/mL), and **21k** (MIC 25μg/mL) exhibited much higher activity. Compounds **21d**, **21f**, and **21g** were equipotent with griseofulvin (100 μg/mL) against *A. niger*, whereas **21e**, **21h**, **21l** (MIC 50 μg/mL), and **21k** (MIC 25 ug) were even 2 to 4-fold more active. On the other hand, all compounds showed moderate to low activity against *A. clavatus* (MIC 100μg/mL) except for **21k** and **21l** (MIC 50 and 25 μg/mL, respectively), which were 2 and 4-fold more potent than griseofulvin, while compound **21h** was equipotent with the mentioned reference drug.

**Figure 10:**
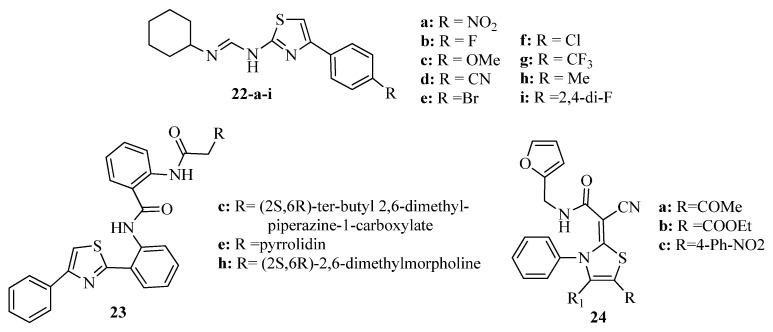
Structures of (E)-N’-cyclohexyl-N-(4-(p-alkyl) thiazol-2-yl) formimidamide and thiazole acetamides (**22**) and (E)-2-cyano-2-(4,5-dialkyl-3-phenylthiazol-2(3H)-ylidene)-N-(furan-2-ylmethyl) acetamide (24).

Łazczkowski et al. [55] reported the synthesis and investigation of antimicrobial activity of other novel thiazole derivatives (**22a**–**22i**, Figure 10) against the panel of fourteen Gram-positive and Gram-negative bacteria strains as well as against fungi *C. albicans* ATCC 2091, *C. albicans* ATCC 10231, *C. parapsilosis* ATCC 22,019, and ten clinical strains of yeast from *Candida spp*., using the broth microdilution method [44,56]. Compounds **22f** and **22h** were found to be more potent with MIC/MBC at 500/1000 μg/mL than ciprofloxacin (MIC/MBC 1000/1000 μg/mL), while compounds **22f** and **22h** were less potent/equipotent to ciprofloxacin against *B. cereus*. All compounds except for **22a** displayed a good antifungal activity that was higher than that of the reference drug (MIC/MFC 1000/>1000 μg/mL) against all fungi species, including clinical isolates. Thus, the MIC/MFC values of compounds **22b**–**22i** were in a range of 0.24–31.25 μg/mL and 1000–>1000 μg/mL. The most resistant fungal strain toward the tested compounds appeared to be *Candida pulcherrima (C. pulcherrima).* The best activity among all compounds tested was achieved by compound **12b** with MIC and MBC values of 0.98 μg/mL against *C. albicans.*

Subhashini et al. [57] designed and synthesized a series of thiazole-acetamide derivatives (**23a**–**j**, Figure 10) and evaluated their antibacterial activities against both Gram-negative and Gram-positive bacterial strains, such as *E. coli, P. aeruginosa*, and *S. aureus*, while *S. pyogenes* was evaluated by a disc diffusion method using norfloxacin (100 μg/mL) as the reference drug. Three compounds **23c**, **23e**, and **23h** (Figure 9) exhibited a good activity against *P. aeruginosa, E. coli, S. pyogenes*, and *S. aureus.* Compounds **23c** and **23e** were the most active against *E. coli* (IZ 18 mm and 19 mm). Their potency was equal to norfloxacin (20 mm), while compound **23e** showed a good activity against *P. aeruginosa* and *S. pyogenes* with inhibition zones of 20 mm and 24 mm, respectively. Norfloxacin was slightly more active (26 mm and 27 mm). Finally, compound **23h** (18 mm) appeared to be almost equipotent with norfloxacin against *S. aureus* (IZ 21mm). Although promising, none of the compounds demonstrated a more potent activity than the reference drug.

Salem et al. [58] evaluated the antimicrobial activity of fourteen synthesized thiazole and thiazolidinone derivatives against Gram-positive bacteria *S. aureus* (RCMB 010027), *S. epidermidis* (RCMB 010024), and *B. subitilis* (RCMB010063). In addition, *P. aeruginosa* (RCMB 010043), *P. vulgaris* (RCMB 010085), and *K. pneumonia* (RCMB 010093) were included in the testing as examples of Gram-negative bacteria as well as a panel of the following fungi *A. fumigatus*, *A. clavatus*, and *G. candidum.* The agar diffusion method was employed with reference drugs gentamycin, ampicillin, and amphotericin B.

The study revealed that derivatives with the electron withdrawal group (**24a**–**24c**, Figure 10) showed good inhibitory activity against *S. aureus, E. epidermitis*, and *B. subtilis* with zone inhibition between 20.6 and 22.3 mm and MIC at 3.9, 1.95, and 0.98 μg/mL, respectively. However, ampicillin was more active MIC 0.24 μg/mL. Only low or moderate activity was observed against other tested bacterial and fungal species.

**Figure 11:**
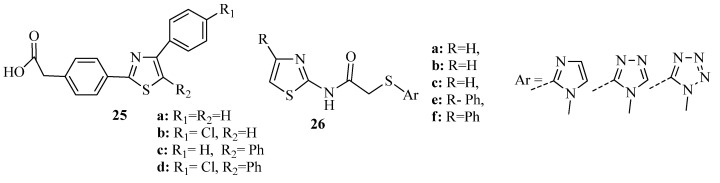
Structures of thiazole phenylacetic acid derivatives (**25**) and 2-[(1/4-methylimidazol/triazol/tetrazol-2/3/5-yl) thio]-*N*-(4-substituted thiazol-2-yl) acetamide derivatives (**26**).

Gençer et al. [59] synthesized new thiazole-phenylacetic acid derivatives (**25a**–**25d**, Figure 11) with potent dual antimicrobial and anti-inflammatory activities. Compounds were evaluated for antimicrobial activity by the microdilution method against *S. aureus* (ATCC 25923), *Enterococcus faecalis* (ATCC 29212), *Listeria monocytogenes* (ATCC 1911), *K. pneumoniae* (NCTC 9633), and *E. coli* (two strains ATCC 35218 and ATCC 25922). Chloramphenicol was used as a control. All compounds showed good antibacterial activity against all bacterial strains. The most active compound **25d** was equipotent to chloramphenicol (MIC 6.25 μg/mL) against all bacteria**.** Compounds **25a**–**25c** (MIC 6.25 μg/mL) were very potent against *K. pneumoniae* and *E. coli*, while against other strains, they exhibited half of the activity of ciprofloxacin. 

Cankılıc et al. [60] presented the synthesis of six 2-[(1/4-methylimidazol/triazol/tetrazol-2/3/5-yl) thio]-*N*-(4-substituted thiazol-2-yl) acetamide derivatives (**26a**–**f**, Figure 11) and evaluation of their antimicrobial activity against different bacteria, yeast, and filamentous fungi. All compounds showed low to moderate antimicrobial activity. Only compounds **26a** and **26c** exhibited 50% inhibition of *L. monocytogenes*. **26b** was active against *L. monocytogenes, K. pneumonia*, and *P. vulgaris*, whereas **26e** and **26f** were active against *Yasimina enterocolitica* (MIC 125 μg/mL).

**Figure 12:**
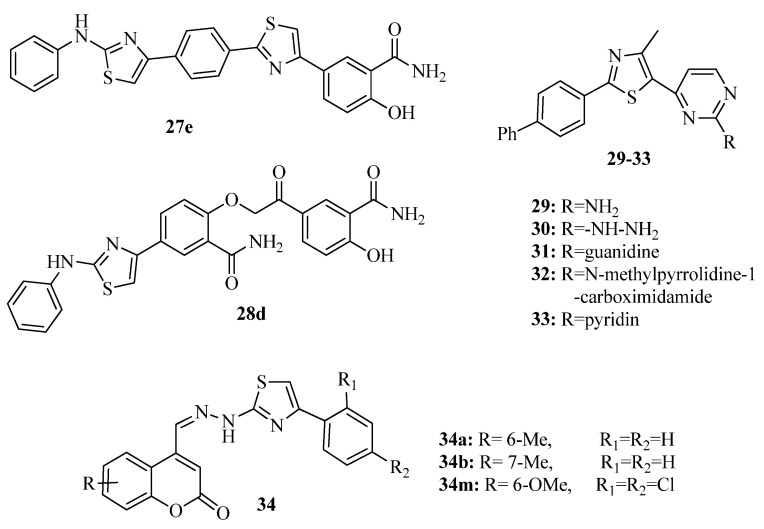
Structures of *N*-phenyl-4-(4-(thiazol-2-yl)-phenyl)-thiazol-2-amine derivatives (27,28), biphenylthiazole analogues, and chlorophenyl thiazolocoumarinyl hydrazides.

Bikobo et al. [61] reported the synthesis and evaluation of antimicrobial activity of fourteen new *N*-phenyl-4-(4-(thiazol-2-yl)-phenyl)-thiazole-2-amine derivatives (**27**, **28**, Figure 12). Compounds were tested against Gram-positive *Enterococcus faecalis (E.faecalis, ATCC 29212), S. aureus (ATCC 29213)*, and Gram-negative *(Salmonella typhimurium, ATCC 14028*) bacterial strains as well as against fungal strains (*C. albicans ATCC 10,231* and *C. krusei ATCC 6258)* using as reference drugs spectinomycin and fluconazole, respectively. All compounds exhibited good antibacterial activity against all species, being twice more potent than spectinomycin (MIC 125 μg/mL) against Gram-positive bacteria *S. aureus* and *E. faecalis*, showing 50% lower inhibitory activity than reference drug (MIC 31.25 μg/mL) against *S. typhimurium.* The highest activity was achieved for compound **27e** against both Gram-positive bacteria with an MIC of 31.25 μg/mL as well as against both fungal species with an MIC of 7.81 μg/mL. Hence, the reference antifungal drug fluconazole (MIC 15.62 μg/mL) was less active. Among the second group of compounds, the most potent activity was achieved in the case of compound **28d** (MIC 62.5 μg/mL) against *E. faecalis*. Again here, the activity was higher than the reference drug (MIC 125 μg/mL), while against all other bacteria and fungi, the activity was only half that of the reference drugs. 

Hagras et al. [62], based on their previous results [53], generated a new hybrid scaffold of 37 biphenylthiazole analogues (**29**–**33**, Figure 12) with the aim to enhance both anti-MRSA activity and drug-like properties. Starting from the lead compound **29** with MIC 6.25 μg/mL against MRSA, some modifications were performed. The structure–activity relationships revealed that the presence of a hydrophobic region around the pyrimidine position-2 is unfavorable. The introduction of a second amino group led to hydrazinyl compound **30** with an activity similar to vancomycin (MIC 1.25 μg/mL). The incorporation of the terminal amine within the morpholine or piperazine moieties resulted in two very active cationic compounds, **31** and **32** (Figure 11) with MIC values of 3.12 and 0.78 μg/mL, respectively. Thus, derivatives with a piperazinine side-chain exhibited a better activity than piperidine analogues. Moreover, aminopiperazine derivative **32** was found to be more potent than the corresponding derivative **33** without amine.

Shaikh et al. [63] synthesized new chlorophenyl thiazolocoumarinyl hydrazides (**34a**–**o**) (Figure 12) with dual antimicrobial and anti-inflammatory activities. For the evaluation of antimicrobial activity, the broth dilution method [56,64] was used with ciprofloxacin and fluconazole, respectively, serving as reference drugs. Most of the compounds except for **34a**–**e** (Figure 11) exhibited exceptional activity against *S. aureus* (MIC at 0.4–0.8 μg/mL) and *E. coli* (MIC at 0.4–1.6 μg/mL), being 2.5–5 times more potent than ciprofloxacin (MIC 2 μg/mL). *B. subtilis* and *P. aeruginosa* were found to be resistant toward the compounds tested. None of the compounds (MIC at 12.6–100 μg/mL) reached the activity of the reference drug. The tested compounds exhibited good antifungal activity and were even more potent than fluconazole (MIC 8 μg/mL) against *A. flavus* (MIC 0.4–3.12 μg/mL) and *A. fumigatus* with MIC being 0.4 μg/mL in all compounds except for **34b** (MIC 0.8 μg/mL). All compounds showed a very good docking score to binding to DNA gyrase (PDB code 1KZN) of *S. aureus*. Compound **34m** formed four hydrogen bonds as well as hydrophobic interactions and seems to be suitable as chemotherapeutic drug against both Gram-positive and Gram-negative bacteria.

**Figure 13:**
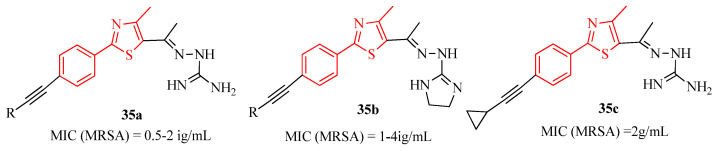
Structure of phenylthiazoles with alkynyl side chain.

Elsebaei et al. [65] evaluated the antibacterial activity of a series of new phenylthiazoles with alkynyl side chain (**35a**–**c**, Figure 13) against multidrug-resistant strains. The most active compound inhibited the growth of clinically applicable MRSA strains with MIC <0.5 μg/mL. The mechanism was suggested to involve inhibition of the cell wall synthesis via two enzymes (undecaprenyl diphosphate phosphatase and undecaprenyl diphosphate synthase). Compound **35c** exhibited good stability to hepatic metabolism (t_1/2_-4.5 h). This compound was found to be equipotent with vancomycin in a neutropenic mouse thigh-infection model at a low dose.

**Figure 14:**
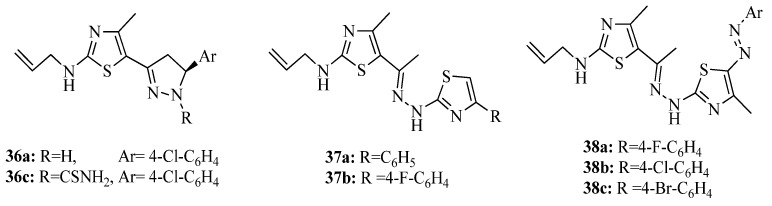
Structures of functionalized 5-hetarylthiazole.

Bondock et al. [66] designed, synthesized, and investigated the antimicrobial activity against the human pathogenic microbes of a new series of functionalized 5-hetarylthiazole. The evaluation revealed that nine compounds had a broad spectrum of activity. Among them, the 5-(2-pyrazolin-3-yl) thiazole (**36a**) (Figure 14) and bithiazolylhydrazone (**38c**) were twice more active than ampicillin against *S. pneumoniae* (MIC 0.06 µg/mL), while 5-(1-thiocarbamoyl-2-pyrazolin-3-yl) thiazole (**36c**) exhibited superior potency as ampicillin toward *S. pneumoniae* and *E. coli* (MIC, 0.03 µg /mL), twofold against *B. subtilis*, and half potency of ampicillin against *S. epidermidis* (MIC, 0.12 µg/ mL). Furthermore, it was found to be equipotent with gentamycin against *K. pneumoniae*. The thiazolyl-hydrazothiazole (**37b**) was superior to ampicillin (MIC 0.03 µg /mL) against *S. pneumoniae*, while compound **38b** (MIC 1.95 µg/mL) was equipotent with gentamycin against *E. coli*. Some compounds displayed good activity also against fungi. E.g., compounds **36c a**nd **38c** were found to be equipotent with amphotericin B (MIC 0.12 µg /mL) against *A. fumigatus*, while compound **38a** (MIC 0.03 µg /mL) demonstrated four-fold higher activity than the mentioned reference drug. Compounds showed a much powerful activity against *S. racemosum.* Ten compounds displayed equal/higher activity with amphotericin B (MIC 7.81 µg /mL), the rest were less potent. The highest activity was achieved against this fungus in the case of compounds **36c**, **37a**, and **37b** (Figure 13) with MIC at 0.24 and 0.06 µg /mL. 

**Figure 15:**
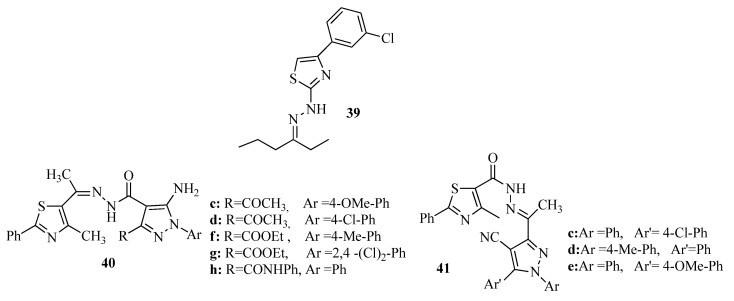
Structure of compounds.

Lino et al. [67] synthesized a novel series of thiazole derivatives (Figure 15) bearing a hydrazone group and evaluated them as well as thiosemicarbazone intermediates against seven fungal strains: *C. albicans, C. krusei, C. parapsilosis, C. tropicalis, Cryptococcus neoformans (C. neoformans), C. gatti*, and *Paracoccidioides brasiliensis* in vitro. The absence of the activity of intermediate compounds indicated the necessity of thiazole ring for antifungal activity. The presence of a hydrophobic aliphatic chain linked to the hydrazone functional group and the chlorine atom in the para position on the benzene ring plays a positive role, since it enhanced the antifungal activity. Compound **39** showed higher activity than fluconazole against all *Candida* and *Cryptococcus* species used in this assay.

Abu-Melha et al. [68] reported the synthesis of a series of thiazole-based heterocycles (**40**–**41**, Figure 15) aiming to study their antimicrobial activity as well as cytotoxicity and hepatoprotective properties. Compounds were tested for their antimicrobial activity against bacteria *S. aureus*,* B. subtilis*,* E. coli*, *P. vulgaris*, and two fungi *A. fumigatus* and *C. albicans* using the agar diffusion method and were compared to standard reference drugs. The most potent appeared to be compounds **40d** and **40f** against *S. aureus* with MIC 156.25 μg/mL and 76.13 μg/mL, respectively, while compound **40g** (MIC 76.13 μg/mL) exhibited the most potent activity against *B. sereus*. Compound **40f** showed also the highest activity (MIC 156.25 μg/mL) against *E. coli*. None of the compounds were active against fungi.

The hepatoprotective potential of newly synthesized thiazole derivatives studied using an in vitro model of CCl_4_-induced hepatotoxicity revealed that **40c**, **40d**, **40f**, **40g**, **40h**, **41c**, **41d** (Figure 15), and **41e** protected the liver against damage, but the effect was lower than the standard drug gentamycin.

**Figure 16:**
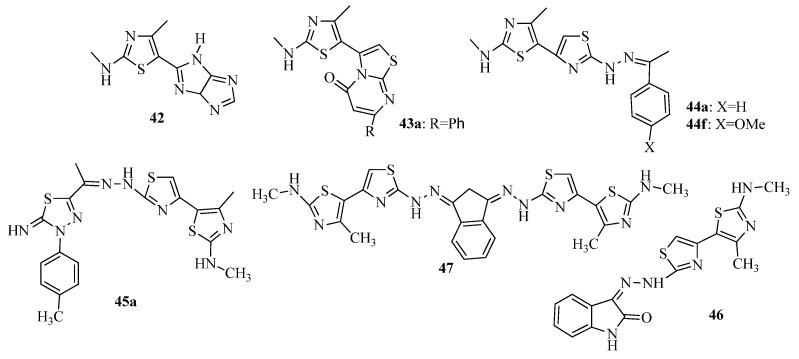
Structures of (Z)-N,4′-dimethyl-2-(2-(1-phenylethylidene)hydrazinyl)-[4,5′-bithiazole]-2′-amine, derivatives, 2-(2-(1-(5-imino-4-(p-tolyl)-4,5-dihydro-1,3,4-thiadiazol-2-yl)ethylidene)hydrazinyl)-N,4′-dimethyl-[4,5′-bithiazole]-2′-amine, (*Z*)-3-(2-(3-(4-methyl-2-(methylamino)thiazol-5-yl)cyclopenta-1,3-dien-1-yl)hydrazono)indolin-2-one, 2,2″-((2Z,2′Z)-2,2′-(1H-indene-1,3(2H)-diylidene)bis(hydrazin-1-yl-2-ylidene))bis(N,4′-dimethyl-[4,5′-bithiazole]-2′-amine).

Athagati et al. [69] synthesized a new series of compounds containing di-, tri-, and tetrathiazole moieties and investigated their antimicrobial activity against four Gram- positive bacteria, four Gram-negative bacteria, and two fungal species. Compounds were synthesized via the reaction of 2-bromo-1-(4-methyl-2-(methylamino) thiazol-5-yl) ethan-1-one with different heterocyclic amines and thiosemicarbazones. The evaluation revealed that compound **42** showed a higher activity (IZ, 24.3mm) than ampicillin (IZ 23.7) against *S. aureus*, while compounds **43a** and **44a** (Figure 16) were equipotent. On the other hand, compounds **43a**, **44a**, **44f**, and **46** showed better activity against *E. coli* (with IZ values of 24.3–26.9 mm), *S. typhimurium* (IZ 24.8–26.7mm), and *K. pneumoniae* (IZ 23.1–26.3mm) than gentamycin (IZ 25.4, 23.3, and 22.6 mm respectively). Compound **44e** exhibited equipotent activity against *K. pneumoniae*, while being more potent than gentamycin against *S. typhimurium* (IZ 22.5 and 26.3 mm, respectively)**.** Compounds **42**, **43a**, **44a**, **e**–**f**, **45a**, **46**, and **47** (IZ 25.0–26.9 mm) exhibited higher activity than amphotericin B (IZ 23.3 mm) against *A. niger*. 

**Figure 17:**
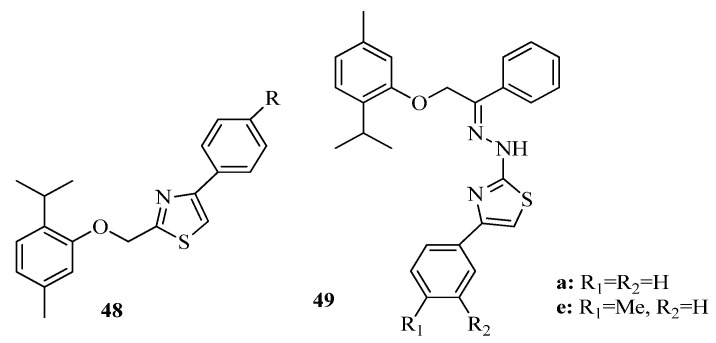
Structures of 4-phenyl-1,3-thiazole and 2-hydrazinyl-4-phenyl-1,3-thiazole derivatives.

Pricopie et al. [70] reported the synthesis of two novel series of 4-phenyl-1,3-thiazole and 2-hydrazinyl-4-phenyl-1,3-thiazole derivatives which were tested in vitro for their effect against three *Candida* sp. It was observed that the 2-hydrazinyl-4-phenyl-1,3-thiazole derivatives (49**a**–**e**, Figure 17) proved to be superior than those with missing the C2-hydrazone linkage (**48a**–**e**) in terms of antifungal potency. Thus, the most active compounds were **49a** (MIC/MFC 7.81/15.62 μg/mL) and **49e** (MIC/MFC 3.9/7.8 μg/mL) being more potent than fluconazole used as the reference drug (MIC/MFC 15.62/31.24 μg/mL) against *C. albicans* (ATCC 10231). The activity against *C. parapsilosis* (ATCC 22019) of these compounds showed half of the fluconazole potency (MIC/MFC 7.81/15.62 μg/mL). No activity against *C. zeylanoides* ATCC 201,082 was found. It was observed that the presence of lipophilic electrone-donating substituent (-CH_3_) in position 4 of the phenyl ring is more beneficial compared to hydrophilic, polar substituents (–CN, –NO_2_, –OH) as regards the antifungal activity.

**Figure 18:**
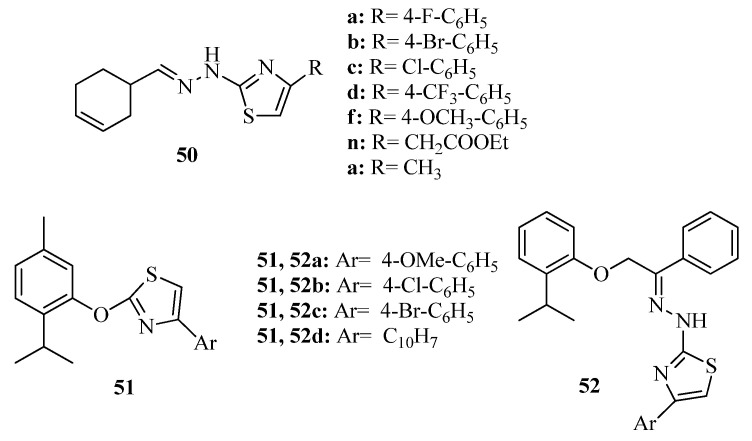
Structures of thiazoles containing cyclohexene moiety and 4-phenyl-1,3-thiazole and 2-hydrazinyl-4-phenyl-1,3-thiazole derivatives.

Biernasiuk et al. [71] synthesized fifteen novel thiazoles containing cyclohexene moiety (Figure 18) and studied their antimicrobial activity against a panel of seven Gram-positive, seven Gram-negative bacteria, and four fungal Candida species as well as 14 clinical isolates. The study revealed that compounds **50a**, **50d**, **50f**, **50n**, and **50o** (Figure 18) showed a very good activity with MIC 0.015–3.91 μg/mL against C. species, exhibiting equipotent or higher activity than nystatin used as the reference drug. The highest activity was achieved in the case of compounds **50n** and **50o** with MIC/MFC at 0.015–0.03 μg/mL/0.015–0.06 μg/mL. Thus, the presence of CH_2_COOEt and CH_3_ groups directly bound to the thiazole ring appeared to be beneficial for anti-candidal activity. On the other hand, compounds **50o** showed also a very strong activity against *B. broncjiseptice* ATTC 4617 with MIC/MFC at 0.06/0.48 μg/mL.

Pricopie et al. [72] taking into account the increasing need for novel antifungal drugs for resistant *Candida sp*. Infection, and based on the results of their previous work [65], synthesized two novel series of 1,3-thiazole derivatives (**51a**–**d**, **52a**, Figure 18) with lipophilic C4 substituent and evaluated their antifungal activity against three human pathogenic Candida species (Figure 17). The investigation revealed that compounds of the second series (**51a**–**d**) showed an excellent activity against *C. albicans* ATCC10231 with MIC/MFC 3.9/7.8 μg/mL, being fourfold more potent than fluconazole (MIC/MFC 15.62/32.24 μg/mL), while against *C. zeylanoides* ATCC 201,082, they had the same activity as fluconazole. Against *C. parapsilosis* ATCC 22,019, these compounds showed half of the fluconazole’s activity, while compounds **51a**–**d** were found to be less potent against all fungal species used.

Thus, 2-hydrazinyl-thiazole derivatives having a lipophilic (+π) para-substituent in the C4 position were the most promising, highlighting that the antifungal activity is dependent on an optimal hydro-lipophilic balance. The molecular docking studies to 14α-lanosterol demethylase (CYP51) were in accordance with experimental data, since the binding affinity increased with increasing the lipophilicity from −10.87 to −13.16, which was probably due to the hydrophobic interactions with the CYP51 lipophilic area.

**Figure 19:**
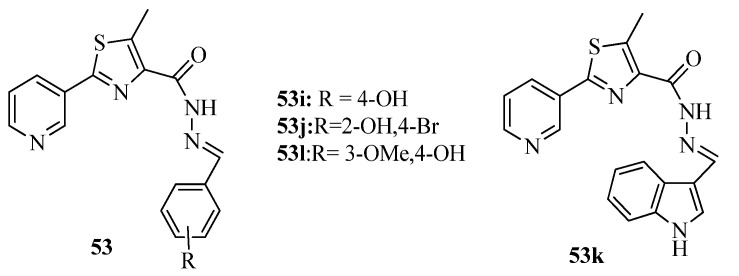
Structure of (E)-N’-substituted-5-methyl-2-(pyridin-3-yl)thiazole-4-carbohydrazides.

Kamat et al. [73] designed and synthesized a new class of compounds by the linkage of −C(O)−NH− between pyridine and thiazole moieties with the aim to study their antimicrobial as well as anti-inflammatory activity. All compounds were tested against antibacterial strains such as *S. aureus (MTCC 9660), B. subtilis (MTCC 441), E. coli (MTCC 443)*, and *P. aeruginosa (MTCC 424)* as well as fungal strains, namely *A. flavus* (MTCC 1316), *Trichoderma atroviridae *(*T. atroviridae*, MTCC 28036), *Penicillium citranum (P. citranum*, MTCC9849), and *C. albicans* (MTCC 461). Results revealed that compounds **53j**, **53k**, **53l**, **53e**, and **53i** (Figure 19) showed good antimicrobial activity with MIC in the range of 1.32–17.33 μg/mL and antifungal with an MIC of 1.66–16.66 μg/mL. The most potent against all bacterial strains with MIC in range of 1.32–2.66 μg/mL was the compound **53j**. It has comparable activity to the reference drugs, tetracycline (MIC at 0.66–1.66 μg/mL) and streptomycin (1.0–1.66 μg/mL). Compound **53j** was found to be also the most active against all fungal strains with MIC in the range of 1.66–2.66 μg/mL. It was more potent than nystatin (MIC 1.66–3.66 μg/mL) against *A. flavus* and *C. albicans*, but it was not more potent than fluconazole (MIC 1.00–2.00 μg/mL) against all fungal species.

The structure–activity relationship studies indicated that the modifications in the aryl ring play an important role for enhancing the activity. The combination of bromo substitution at the 5th position and hydroxy group at the 2nd position (**53j**) was found to be beneficial in enhancing antimicrobial potency.

### 3.2. Antituberculosis

Tuberculosis (TB) is a contagious disease causing the death of many millions of people around the world [74]. Due to the growth of resistant strains of *Mycobacterium tuberculosis (M. tuberculosis)*, it continues to be one of the most serious infectious diseases. According to the World Health Organization (WHO), 1.4 million people died from TB in 2019, which was the highest number of deaths from any infectious disease worldwide. Additionally, TB is listed in the top 10 causes of death worldwide [75].

Despite TB being both preventable and curable by treatment through standard antitubercular drugs, such as isoniazid, rifampicin, ethambutol, and pyrazinamide used for decades [76], according to WHO reports, the cases of TB fall by only about 2% per year. Furthermore, cases of Multidrug Resistant-TB (MDR-TB) are increasing each year and continue to remain major public health crises and health threats. Thus, there is an urgent need for a new antitubercular agent against resistant strains.

**Figure 20:**
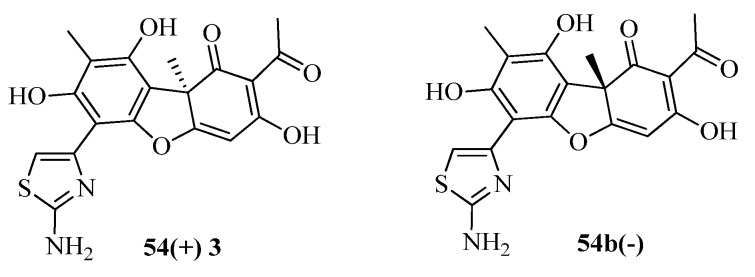
Structures of new usnic acid derivatives.

Bekker et al. [77] reported an in vitro investigation of the potential inhibitory activity of new usnic acid derivatives on the growth of *M. smegmatis* and *M. tuberculosis*. The study revealed that the most active compounds contain an amino group in the thiazole ring. Both **54a** (−) (−3) and **54b** (+) (−3) (Figure 20) isomers were tested for their kinase inhibitory activity. Significant protein kinase activity was observed in the *S. lividans* APHVIII+ and *M. smegmatis* APHVIII+ test systems. It was found that thiazole derived from (−) usnic acid was more potent than (+) isomer against the *M. smegmatis* kanamycin-resistant strain APHVIII+, while both isomers demonstrated the same activity as the compound **53j**. As far as *M. tuberculosis* H37Rv is concerned, both isomers exhibited high inhibitory activity with an MIC99 of 25 μg/mL and an MIC100 of 50 μg/mL. Again, isomer **54b** (−3) was more active than **54a** (+3). 

**Figure 21:**
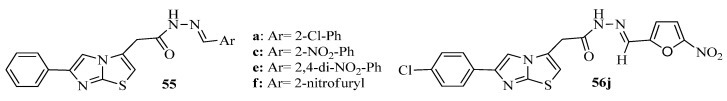
Structures of *N’*-(arylidene)-2-[6-(4-bromophenyl) imidazo[2,1-*b*]thiazol-3-yl]acetohydrazides.

Guzeldemirci et al. [78] synthesized new *N’*-(arylidene)-2-[6-(4-bromophenyl) imidazo[2 ,1-*b*]thiazol-3-yl]acetohydrazides (**55a**–**I**, Figure 21) with the purpose to evaluate their *in vitro* antimycobacterial activity against *M. tuberculosis* H37Rv. Compounds were studied using a BACTEC 460 radiometric system by a broth microdilution assay with rifampicin as the reference drug. It was found that compounds exhibited varying degrees of inhibition in a primary test performed in a concentration of 6.25 μg/mL. Only compounds **55a**, **55c**, **55e**, and **55f** showed >90% inhibition in the primary screen at 6.25 μg/mL. The most active compound among these four chosen was **55e** with 99% inhibition and IC_50_ >1.6 mg/mL. On the other hand, compound **55c** showed even lower inhibition (91%) and IC_50_ 1.05 μg/mL.

In their next work, Guzeldemirci et al. [79] reported on the synthesis of arylidenehydrazide derivatives bearing imidazo[2,1-b] thiazole moiety (**56a**–**j**, Figure 21) in order to study their antitubercular activity against *M. tuberculosis* H37Rv (ATCC 27294). All compounds were evaluated in BACTEC 12B medium using a broth microdilution assay [80] showing a weak activity with IC_50s_ in the range of 6.16–100 mg/mL compared to rifampicin (IC_50_ of 0.125 μg/mL) used as the reference drug. The most potent among all compounds tested was compound **56j** (IC_50_ of 0.125 μg/mL). It was found equipotent to rifampicin.

**Figure 22:**
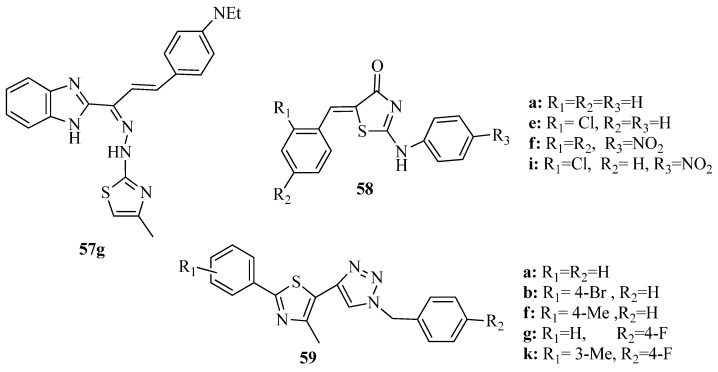
Structures of benzimidazolallylidenehydrazinylmethylthiazole derivatives, (*Z*)-5-(substituted benzylidene)-2-((substituted phenyl)amino)thiazol-4(5H)-one analogues, and 5-(1-benzyl-1H-1,2,3-triazol-4-yl)-4-methyl-2-arylthiazole derivatives.

Zhang et al. [81] presented the design, synthesis of benzimidazolallylidenehydrazinylmethylthiazole derivatives (**57a**–**o**, Figure 22), and their screening as well as their precursors for in vitro antimycobacterial activity against the selectable *M. tuberculosis* strain H37Ra [82,83]. All tested compounds (30) showed in vitro antimycobacterial activity against *M. tuberculosis* with MICs in range of 2–100 μg/mL. Most of the compounds exhibited moderate activity (MIC ≤ 10 μg/mL). The most active appeared to be compound **57g**, showing the same MIC of 2.5 μg/mL. However, it was less active compared to the reference drug. Since compound **57g** was the less toxic one, it is promising for further optimization.

Shelke et al. [84] reported on the synthesis of (*Z*)-5-(substituted benzylidene)-2-((substituted phenyl) amino) thiazol-4(5H)-one analogues **58a**–**j**, Figure 22) with the aim of studying their antituberculosis activity. Compounds were tested for their ability to inhibit the growth of an avirulent strain of *MTB H37Ra* (ATCC 25177) and *M. bovis BCG* (ATCC 35743) following a screening protocol [85,86] using rifampicin and isoniazid as reference drugs. The study revealed that only compounds **58e** and **58f** showed more or less strong activity with MIC_90s_ of 26 and 25 μg/mL, respectively, against *MTB H37Ra*, while compounds **58a** and **58i** were active against *M. bovis BCG* strain with MIC_90_ values of 15 and 12 *μ*g/mL, respectively. The reference drugs rifampicin and isoniazid have MIC_90_ values of 0.044 ± 0.14 and 0.070 ± 0.25 μg/mL, respectively. All compounds were found to be not very potent.

A series of new 5-(1-benzyl-1H-1,2,3-triazol-4-yl)-4-methyl-2-arylthiazole derivatives (**59a**–**w**) were synthesized by Shindle et al. [87] for the evaluation of their potential antimycobacterial activity. Compounds were tested in vitro against *M. tuberculosis* H37Ra (dormant) using the XTT reduction menadione assay [88,89,90]. The evaluation of antituberculosis activity revealed that the majority of compounds showed good activity with IC_50_ values in the range of 0.58-1.74 μΜ. Among the group of compounds, **59a**–**f** (Figure 22), with unsubstituted phenyl ring A and substituted phenyl ring B, compounds **59a**, **59b,** and **59f**, showed good activity with IC_50_ values of 0.84–1.28 μΜ. However, rifampicin had better effect (IC_50_ = 0.02μΜ). The highest activity was observed among the compounds **59g**–**l** with IC_50_ values of 0.58–0.83 μg/L. Compounds **59g** and **59k** exhibited lower activities with MIC_90_ values of 4.41 and 2.2 mg/mL. These compounds were further tested on two cancer cell lines (HeLa and THR-1), and they were found to be nontoxic with IC_50_ >80 μg/mL. Compounds were docked to enoylacyl carrier protein reductase, which is a key enzyme involved in the metabolism and many conservative processes in mycobacterium in order to find the mechanism of their action. Compound **59g** showed aromatic and hydrophobic interaction with amino acid residues of the enzyme as well as van der Waals interactions, while compound **59k** formed a hydrogen bond with MET103 except for almost the same aromatic, hydrophobic, and van der Waals interactions.

**Figure 23:**
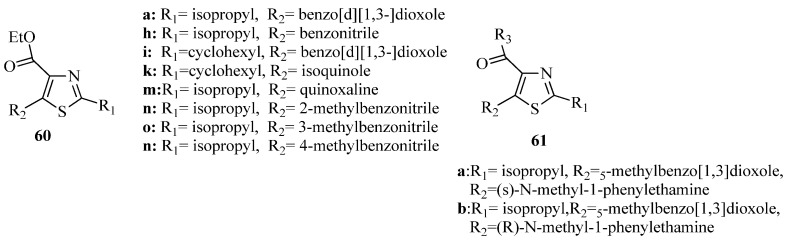
Structures of 2,4,5-trisubstituted thiazoles.

Karale et al. [91] synthesized a series of 2,4,5-trisubstituted thiazoles (50) and evaluated their inhibitory potential against *M. tuberculosis* strain H37Rv using MABA assay. Compounds **60a**–**h** (Figure 23) showed antituberculosis activity with an MIC range of 1.8–40 μg/mL. Among all these compounds, only **60h** with an isopropyl substituent and **60p** with cycloxehyl moiety showed good activity with MIC values at 1.8 μg/mL and 2.1 μg/mL, respectively, compared to rifampicin (IC_50_ = 0.2 μg/mL). Compounds **60d** and **60k**–**o** showed moderate activity, with MIC values in the range of 4.0–4.5 μg/mL, while the rest of the compounds were less active. In order to improve the activity, an additional 32 compounds were synthesized by introducing a third substituent. The investigation revealed that compounds **60a**, **60i**, **60k**, **60m**–**p**, **61a**, and **61b** showed the ability to inhibit dormant *M. tuberculosis* H37Ra by more than 90% at 10 μM concentration

Next, the compound **60p** was tested against a panel of drug-resistant TB isolates and showed an MIC of 8 μg/mL against isolates 1, 2, 5, and 6, while an MIC of 16, 6, and 16 μg/mL was reported against MDR-TB isolates 3, 4, and 7. Among them, isolate 4 was resistant against four drugs. Finally, compounds with an MIC ≤ 4.2 μg/mL were found to be nontoxic against CHO-KI cells.

**Figure 24:**
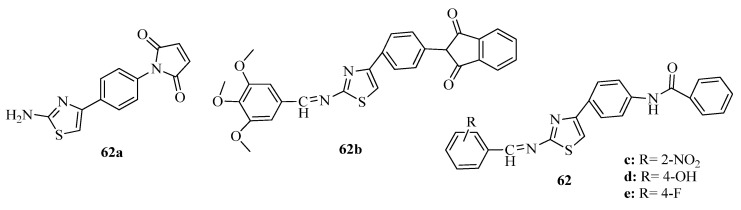
Structures of 2-amino thiazole Schiff bases.

Cordeiro et al. [92] reported the synthesis of 2-amino thiazole Schiff bases (Figure 24) and evaluation of their anti-tuberculosis activity against *Mycobacterium tuberculosis* H37Rv strain by the Microplate Alamar Blue assay (MABA) method. As reference drugs isoniazid, ethambutol, pyrazinamide, rifampicin, and streptomycin were used. Only four compounds N-(4-(2-((2-nitrobenzylidene)amino) thiazol-4-yl)phenyl)benzamide (**62c**, Figure 24), N-(4-(2-((4-hydroxybenzylidene)amino)thiazol-4-yl) phenyl)benzamide (**62d**), 2-(4-(2-((3,4,5-trimethoxybenzylidene)amino)thiazol-4-yl)phenyl)-1H-indene-1,3(2H) dione (**62b**), and 1-[4-(2- amino-thiazol-4-yl)-phenyl]-pyrrole-2,5-dione (**62a**) showed a substantial activity with MIC values of 6.25 μg/mL, being twofold less active than pyrazinamide but much less potent than the other reference drugs. Lower activity was found for N-[4-(2-amino-thiazol-4-yl)-phenyl]-benzamide and 1-[4-(2-amino-thiazol-4-yl)-phenyl]-pyrrole-2,5-dione derivatives with 4-fluoro substitutions on the benzene ring (**62e** and **62a**, respectively) with MIC at 12.5 μg/mL. The rest of the compounds showed only a low activity.

### 3.3. Thiazole Derivatives as Anticancer Agents

Cancer is one of the major public health problems in most countries around the world [93]. The disease is associated with genetic acquired or inherited abnormalities [94,95].

There are a numerous number of antitumor agents present in the literature and commercially available; unfortunately, most of them exhibit high toxicity and develop resistance, which may lead to failure in the treatment. Thus, there is a huge interest to develop promising new prototypes of anticancer drugs.

Braga et al. [96] synthesized a series of thiazole derivatives with the purpose to investigate their cytotoxicity against three human cancer cell lines: HL-60 (promyelotic leukemia), Jurkat (acute lymphoblastic leukemia), and MCF-7 (breast cancer) as well as normal (Vero cells) cell lines. Compounds were tested in vitro at a concentration of 50 μΜ, while the reference drug etoposide was investigated at 10 μΜ.

**Figure 25:**
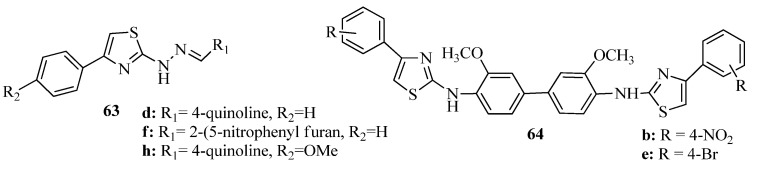
Structures of hydrazinil and bis-thiazole derivatives.

The study revealed that only three compounds **63d**, **63f**, and **63h** (Figure 25) were able to inhibit the viability of breast carcinoma cells (MCF-7) with IC_50_ values of 54, 43, and 76 μM, respectively while derivative **63h** was found to be active against promyelocytic leukemia cells (HL-60) with an IC_50_ value of 43 μM. None of compounds was found to be significantly cytotoxic against Jurkat cells [97].

The pro-apoptotic potential of the more promising compounds (**63d**, **63f**, and **63h**) (Figure 25) was tested and found that the percentage of hypodiploid DNA varied for HL-60 and MCF-7 cells. Thus, **63h** showed 90.9 ± 2% hypodiploid DNA against HL-60, while against MCF-7, the percentage for compounds **63d**, **63f**, and **63h** was from 45% to 53.8%. Since compound **63h** induced DNA fragmentation in both lineages (p53-null and wild type), and taking into account that the HL-60 cell line does not express p53 protein, indicated that apoptosis induced by **63h** is not dependent on this pathway.

Based on literature data regarding the nature of bis-thiazole-DNA interaction [98,99,100,101,102,103,104] and also the fact that dasatinib, a second-generation tyrosine kinase inhibitor, has a bis-thiazole moiety [105] and encouraging previous results [106,107], Turan-Zituni et al. [108] synthesized new bis-thiazole derivatives (**64a**–**j**, Figure 25) as anticancer drugs. Compounds were tested against A549 human lung adenocarcinoma, C6 rat glioma, 5RP7 H-rasoncogene transformed rat embryonicfibroblast, and NIH/3T3 mouse embryonic fibroblast cell lines using MTT assay.

The evaluation revealed that in general, all compounds showed moderate to low effect on these cell lines, exhibiting more significant cytotoxicity against C6 rat glioma cell lines than toward A549 human lung adenocarcinoma. The best activity among all compounds tested against C6 and A549 lines was achieved for compound **64e** with IC_50_ values of 11.3 ± 1.2 µg/mL and 37.3 ± 6.8 µg/mL, respectively. These results are very encouraging, since mitoxanthone, which was used as a reference drug, had an IC_50_ of 5.7 ± 4.0 µg/mL. Compound **64b** displayed the highest activity against 5RP7 cell lines with an IC_50_ of 5.83 ± 1.04 µg/mL. Mitoxanthone (IC_50_ = 0.73 ± 0.06 µg/mL) was more active. None of the compounds exhibited good activity against NIH/3T3 mouse embryonic fibroblast cell lines.

**Figure 26:**
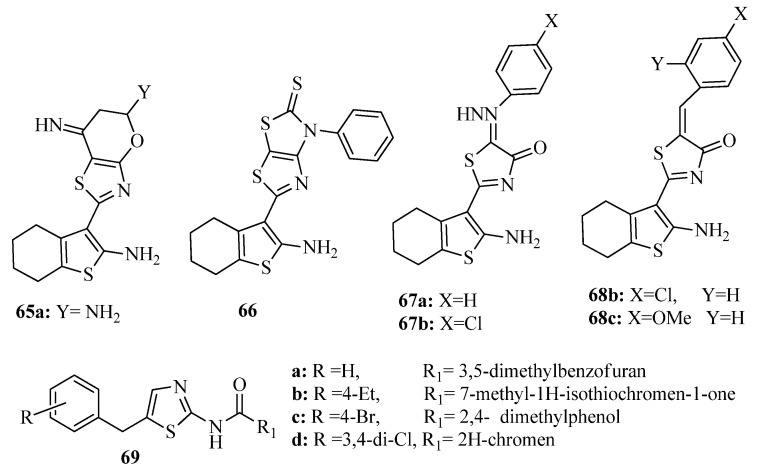
Structures of 2-amino-4,5,6,7-tetrahydrobenzo[b]thiophene-3-carbonitrile and *N* acylated 2-amino-5-benzyl-1,3-thiazoles.

Mohareb et al. [109] synthesized a series of heterocyclic compounds (Figure 26) and evaluated their cytotoxic activity against six human cancer cell lines, namely: human gastric cancer (NUGC), human colon cancer (DLD-1), human liver cancer (HA22T), and HEPG-2), human breast cancer (MCF-7), and nasopharyngeal carcinoma (HONE-1) as well as against normal fibroblast cells (WI-38). All compounds showed different activity against different cell lines. Thus, compound **67a** displayed the highest activity against the NUGC cell line with an IC_50_ value of 23 ± 80 nmol/L, which was not different to that of the reference compound CHS-828 (IC_50_ = 25 ± 10 nmol/L), while **67b** exhibited tenfold higher activity (IC_50_ = 120 ± 38 nmol/L) against HEPG-2 than the reference drug (Figure 26. On the other hand, compound **68b** with an IC_50_ = 24 ± 18 nmol/L showed much higher cytotoxicity against DLD-1 than the reference drug (IC_50_ + 2315 ± 13 nmol/L), which was followed by **66** (IC_50_ = 55 nmol/L). Compound **69c** was the only potent compound against the MCF-7 cell line being equipotent with the reference drug (IC_50_ = 18 ± 70 nmol/L). Finally, thiazole derivative **65a** (IC_50_ = 59 ± 22 nmol/L) showed an excellent activity that was 35 times higher than the reference drug against HA22T. The second thiazole derivative 66 was found to be potent against NUGC, but it was almost twice less potent than the reference drug. Against DLD-1 (IC_50_ = 55 ± 12 nmol/L), it was much more potent than the comparator CHS-828.

Finiuk et al. [110] reported the synthesis of N-acylated 2-amino-5-benzyl-1,3-thiazoles (**69a**–**d**) (Figure 26) and evaluated their antitumor activity toward several human cancer cells. Initially, compounds were studied against adenocarcinoma cells, namely human breast adenocarcinoma cells MCF-7 line and human lung adenocarcinoma cells A549 line by MTT assay using doxorubicin as the reference drug. The study demonstrated that all thiazole derivatives used in doses up to 50 μM were less toxic than doxorubicin, which showed stronger cytotoxicity for MCF-7 cells compared to A549. The study of the antineoplastic effects of synthesized compounds against human glioblastoma cells of U251 line and human melanoma cells of WM793 line indicated the concentration-dependent cytotoxicity against glioblastoma cells. Compound **69a** displayed a high toxic effect toward the glioblastoma cells (IC_50_ = 9.8 ± 0.82 μM), being more toxic than doxorubicin (IC_50_ = 21.0 ± 1.64 μM). The same behavior of these compounds was observed toward melanoma cells WM793 line with compounds **69b**, **69c**, and **69d** showing IC_50s_ in a range of 28.5–34.6 μM, whereas the most toxic appeared again to be **69a** (IC_50_ = 7.2 ± 0.48 μM). Its effect was comparable to doxorubicin (IC_50_ = 6.1 ± 0.38 μM). Nevertheless, none of these compounds demonstrated significant antineoplastic effect toward human myeloid leukemia cells K562 line (IC50 = 40.0–44.7 μM) and human acute T-cell leukemia cells of Jurkat line (IC_50_ = 27.0–33.0 μM). However, doxorubicin was markedly more potent (IC_50_ = 0.7 ± 0.05 μM). Anyway, thiazole derivatives demonstrated a wide spectrum of growth inhibition against human tumor cells of different tissue origin, being more cytotoxic for melanoma and glioma cells.

Gomha et al. [111] synthesized a series of arylazothiazoles (Figure 26) and evaluated their in vitro growth activity against hepatocellular (HepG2) and colorectal (HCT-116) carcinoma cell lines by colorimetric MTT assay with 5-fluorouracil and cisplatin as reference drugs. The study revealed that in general, all compounds showed moderate to good activity against both cell lines

**Figure 27:**
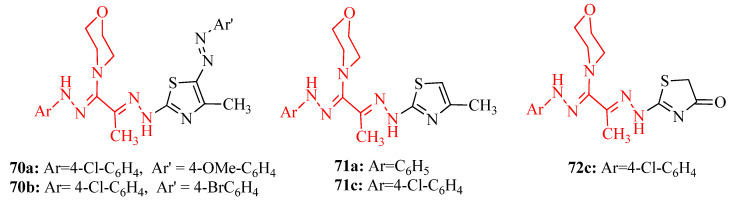
Structures of arylazothiazoles.

Compounds **70a**, **70b**, **70c**, **71c**, and **72c** (Figure 27) with IC_50_ values ranging from 4.9 to 9.3 μM showed the highest activity among all compounds tested against hepatocellular (HepG2) carcinoma cells, with **72c** (IC_50_ = 4.9 ± 0.5 μM) being even more potent than cisplatin (IC_50_ = 6.9 ± 0.7 μM). Similarly, compounds **71a** and **72c** were the most potent against colorectal (HCT-116) carcinoma cell lines (IC_50_ = 10.1 ± 1.2 and 7.3 ± 0.9 μM) compared to cisplatin (IC_50_ = 3.1 ± 0.6 μM).

It seems that the presence of a chlorophenyl substitution at phenyl hydrazine moiety at the 2nd position (**71c**, **70c**) is favorable for anticancer activity, while the arylazo group at the thiazole ring decreased the activity (**70a**, **70b**) compared to **71c** and **72c.** The most active compounds **70b**, **71a**, **71b**, and **72c** showed the best docking score with the active site of the c-kit receptor protein-tyrosine kinase.

**Figure 28:**
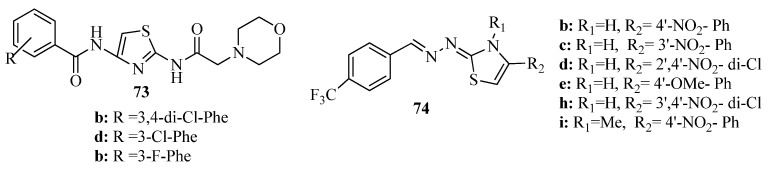
Structures of substituted 2-amino-4-phenylthiazole derivatives and (Z)-3,4-disubstitutedl-2-((E)-(4-(trifluoromethyl) benzylidene) hydrazono)-2,3-dihydrothiazole.

Based on the structural characteristics of crizotinib, Zhang et al. [112] designed and synthesized a series of appropriately substituted 2-amino-4-phenylthiazole derivatives (**73a**–**o**) (Figure 28) as the mesenchymal–epithelial transition factor (c-Met) kinase inhibitors. The target compounds were studied for antiproliferative activities by the MTT method using human non-small cell lung cancer cell A549, human cervical cancer cell HeLa, human colon cancer cell HT29, and human lymphoma cell line Karpas299. Crizotinib served as positive control. The study revealed that compounds **73b**, **73d**, and **73g** exhibited strong growth inhibitory effects, especially for HT29 cells with IC_50s_ of 2.01, 2.19, and 5.22 μM, respectively. The effect was comparable to that of crizotinib (IC_50_ = 1.1 μM). Compound **73b** also showed better growth inhibitory effect than others for Karpas 299 (IC_50_ = 4.46 μM) and HeLa cells (IC_50_ = 9.56 μM). The structure–activity studies indicated that the most beneficial activity is meta substitution on benzene ring 2, especially Cl substitution. It was found that 3,4-di-Cl (**73b**) and 3-Cl (**73d**) derivatives are more potent than 2,4-di-Cl (**73c**) derivative. The replacement of a phenyl ring by furyl was not favorable for the activity and indicated the importance of the phenyl ring. In an in vitro enzymatic assay, compound **74b** displayed a moderate c-Met enzymatic inhibitory potency. Thus, it can be concluded that inhibition of c-Met kinase is probably the main mechanism of the antiproliferative activity of compound **73b**. The docking studies showed that the thiazole ring of compound **73b** formed a π–π interaction with Tyr 1230 of the c-Met protein, similar to that observed for crizotinib.

De Santana et al. [113] presented the synthesis of a series of thiazole derivatives with the aim to receive high specificity to tumor cell and low toxicity to the organism. Compounds were evaluated for their cytotoxic activity against five tumor cell lines, namely NCI-H292, HEp-2, HT-29, HL-60, K562, and six compounds (**74b**–**e**, **74h**, **74i**, Figure 28) showed significant cytotoxic activity in at least three tumor cell lines. As a reference compound, doxorubicin was used. The evaluation revealed that disubstituted compounds 2,4-dichloro (**74d**) and 3,4-dichloro (**74h**) displayed the best activity against four tumor cell lines (NCI-H292, HEp-2, HT-29, K5620) with IC_50_ values of 10.41 ± 0.55, 7.65 ± 0.83, 11.15 ± 2.70, and 5.48 ± 0.85 μΜ, respectively. NCI-H292, HEp-2, HT-29, and HL-60 had IC50 values of 6.57 ± 1.38, 10.38 ± 2.15, 12.21 ± 1.71, and 4.23 ± 0.81 μΜ, respectively. Thus, the presence of di-Cl substitution was beneficial for cytotoxic activity. Compound **74b** with an electron withdrawal group (4-NO_2_) was found to be more active against NCI-H292 with an IC_50_ of 7.41 ± 1.37 μΜ than **74c** with a 3-NO_2_ group (IC_50_ of 22.77 ± 2.77 μΜ), indicating the importance of not only the nature but also of the position of the substituent at the thiazole ring as far as the activity is concerned.

Compound **74d**, as the most promising, was evaluated for its mechanism of action by flow cytometry. The results demonstrated cell cycle arrest in the G1 phase, DNA fragmentation, and alteration in mitochondrial membrane, suggesting cell death due to apoptosis with involvement of the mitochondrial pathway in the mechanism of action. Nevertheless, to prove this, some complementary studies are needed.

**Figure 29:**
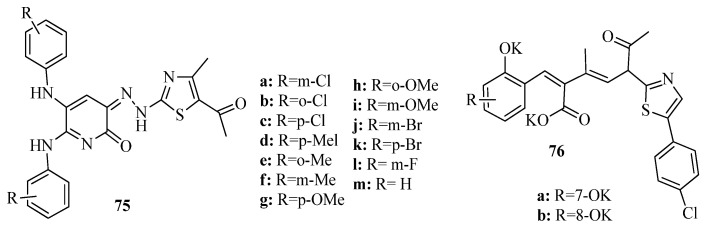
Structures of pyridinethiazole hybrids and 5-(4-chlorophenyl)-2-[(7/8-hydroxycoumarin -3-ylethylidene)acetylhydrazine]-1,3-thiazoles.

Xie et al. [114] designed, synthesized, and studied the in vitro antiproliferative activity of a new series of pyridinethiazole hybrid compounds (**75a**–**m**, Figure 29). The antiproliferative activity was evaluated against colon cancer (HCT-116), gastric carcinoma (MGC803), and hepatocellular cancer (Huh7) by the MTT (3-(4,5-dimethylthiazol-2-yl)-2,5-diphenyltetrazolium bromide) method, using 5-fluorouracil (5-FU) as positive control. The study revealed that most of the tested compounds exhibited potent cytotoxicity especially against colon HCT-116 and gastric carcinomas MGC803.

Compounds **75a**, **75b**, and **75c** with IC_50_ values between 8.17 and 10.59 μΜ against HCT-116 and 3.15–8.56 μΜ exhibited good activity against MGC803 and HCT-116 cell lines higher than 5-FU (IC_50_ = 11.29 ± 1.06 μΜ and 25.54 ± 0.05 μΜ, respectively). Compound **75b** was found to be the most potent against these two cell lines with IC_50_ values of 3.15 ± 1.68 μΜ and 8.17 ± 1.89 μΜ, respectively.

It was observed that the presence of electron-withdrawal groups (Cl, Br, F) on the benzene ring (**75a**–**c**, **75j**, **75k**, **75l**) favored the anticancer activity, enhancing it compared to an unsubstituted derivative (**75m**), while electron-donating groups (Me, OMe) had the opposite effect, decreasing the activity compared to derivative **75m**. Among the three most active compounds, the highest was achieved for compound **75b** (IC_50_ 8.17 ± 1.89 μΜ and 3.15 ± 1.68 μΜ), being more potent against the gastric carcinoma (MGC803) cell lines. This is an indication that not only the nature but also the position of the substituent affects the anticancer activity.

Mohammed et al. [115], based on the literature survey regarding the antitumor activity of coumarin compounds [116,117] as well as thiazole derivatives [118], combined these two moieties in one molecule and synthesized the potassium salt of 5-(4-chlorophenyl)-2-[(7-hydroxycoumarin-3-ylethylidene)acetylhydrazine]-1,3-thiazole (**76**a) and 5-(4-chlorophenyl)-2-[(8-hydroxycoumarin-3-ylethylidene)acetylhydrazine]-1,3-thiazole (**76b**) with the aim to investigate in vivo their antitumor activity (Figure 29). Compounds were tested for their effect on caspase-3 and Bax (BCL-2 associated protein) in Ehrlich ascites carcinoma (EAC) and in the liver. Compounds **76a** and **76b** increased the life span of EAC-bearing mice due to the reduced viability of EAC cells and decreasing tumor value, thus proving the result of Gabr [119].

The mean values of caspase-3 in the liver tissue were found to be 3.07 ± 0.119 (ng/mL) in the negative control group, which decreased in the positive control group by 70.57%, while the mean values of caspase-3 activities for both therapeutic and preventive groups treatments with compounds **76a** and **76b** significantly increase by 13.25% and 229.4%; 116.9% and 139.77%; respectively. Compound **76b** was more active than **76a** against the EAC of mice.

**Figure 30:**
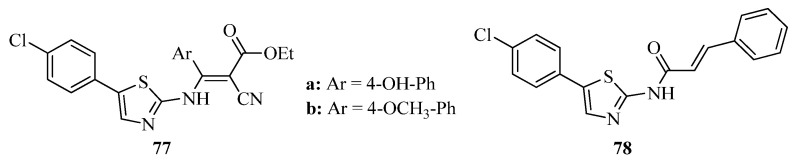
Structure of 2-(substituted)amino-1,3-thiazole derivatives.

Bakare [120] reported the synthesis of a new series of 2-(substituted) amino-1,3-thiazole derivatives and evaluation of their effect on the viability of the Leukemia HL-60 cell line using a colorimetric MTT assay. Compounds **77a**, **77b**, and **78** (Figure 30) exhibited the best activity among all compounds tested with IC_50_ values in range of 1.3–8.9 μM. The highest activity was achieved for compound **77b** with an IC_50_ value 1.3 ± 0.29 μM higher than that of doxorubicin (IC_50_ = 3.01 ± 0.07 μM), which was used as the reference drug. Compound **77b** was further subjected to cell cycle analysis using DNA flow cytometric assay [121] and showed 37.08% of cells in G2/M phase in comparison with the untreated cells, which had 7.13%. Next, the ability to activate caspase-3 was studied. The obtained data demonstrated that compound **77b** is able to enhance the concentration of caspase-3, since the optical density values were increased by 4.55-fold compared with the untreated control. Thus, these compounds can be considered as a scaffold for the design of new anticancer agents.

**Figure 31:**
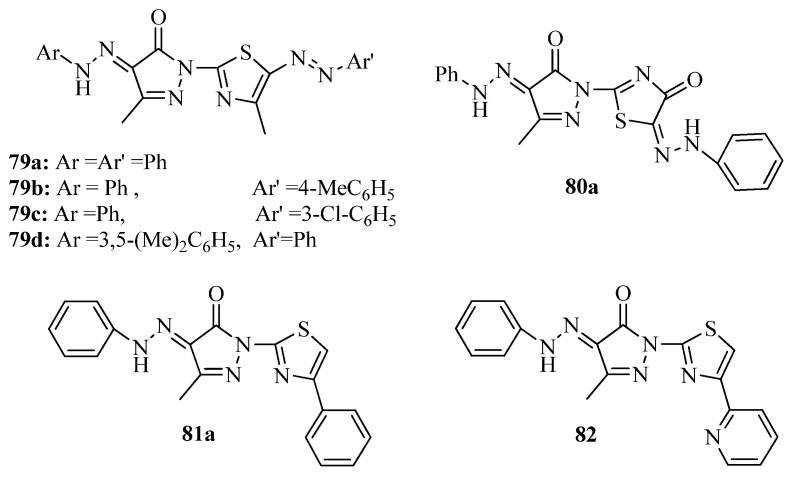
Structures of thiazolyl-pyrazole derivatives.

Sayed et al. [122] synthesized a series of novel thiazolyl-pyrazole derivatives and evaluated against the human liver carcinoma cell line (HepG-2) using MTT assay and doxorubicin as a reference drug. Among all the tested compounds, **79d**, **80a**, **81a**, and **82** (Figure 31) exhibited good anticancer activity with IC_50_ values ranging from 1.25 to 4.8 μg/mL compared to doxorubicin (IC_50_ of 3.07 ± 0.27 μg/mL). The best activity was achieved for compound **81a** with IC_50_ values of 2.20 ± 0.13 μg/mL, followed by compound **79d** with IC_50_ = 3.90 ± 0.41 μg/mL. The presence of two methyl groups in positions 3 and 5 of the phenyl ring seems to be beneficial for anticancer activity, since this derivative is more active than **79a**–**c** with Ar unsubstituted. In general, the activity of compounds followed the order: **80a** > **79d** > **82** > **81a**. According to docking results on the EGER kinase active site, all compounds showed low binding energy from −3.4 to −1.3 kcal/mol with an order that confirms the experimental findings.

**Figure 32:**
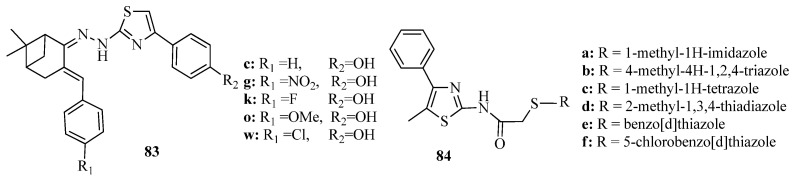
Structures of *β*-pinene-based thiazole derivatives and N-(5-methyl-4-phenylthiazol-2-yl)-2-(substituted thio) acetamides.

Wang et al. [123] based on the data from the literature [113,114,124] and as a continuation of their previous work on the synthesis and anticancer activity of triterpene derivatives [125,126] designed 24 compounds that were evaluated for cytotoxicity against three cancer cell lines (colon cancer CT-26 cells, human cervical carcinoma, HeLa cells, and human hepatocarcinoma SMMC-7721 cells). The evaluation was performed using the MTT assay with etoposide as a reference drug. The study revealed that compounds **83c**, **83g**, **83k**, **83o**, and **83w** (Figure 32) exhibited a strong cytotoxic effect against all three cancer cell lines, indicating the beneficial role of a strong electron-donating hydroxyl group in position 4 of the benzene ring as a substituent at the thiazole ring (R_2_). On the other hand, the presence of an electron-withdrawing group (R_2_ = F) as well as OCH_3_ and CH_3_ groups as R_1_ substituents had a negative effect, decreasing the anticancer activity or leading to inactive compounds respectively, while compounds **83d**, **83h**, and **83x**, bearing a methoxy group such as R_2_ showed moderate activity.

Compounds **83c** and **83w** displayed slightly weaker activity against SMMC-7721 cells, while **83g** was the most potent against cervical carcinoma HeLa cells. In general, compound **83g** appeared to be the most active with IC_50_ values ranging from 3.48 to 8.84 μM. According to the mechanistic studies, the compound induced apoptosis via the mitochondria-mediated apoptosis pathway.

Based on their previous data [127,128,129], Evren et al. [130] synthesized new N-(5-methyl-4-phenylthiazol-2-yl)-2-(substituted thio) acetamides (**84a**–**f**, Figure 32) and evaluated their anticancer activity against A549 and NIH/3T3 cell lines by MTT assay with cisplatin as the reference drug. The highest cytotoxic activity against A549 cells (IC_50_ of 23.30 ± 0.35 μM) was achieved for compound **84c**, but it was not toxic against NIH/3T3 cells.

The study of their apoptotic effect on A549 human lung adenocarcinoma revealed different ways of killing these cells. Thus, compounds **84b**, **84e**, and **84f** killed the biggest percentage of A549 cells by the way of necrosis, while compounds **84a** and **84c** killed them through apoptosis. From the obtained results, it is obvious that **84c** (1-methyl-1*H*-tetrazol-5-yl) is both selective and the most active compound against A549 lung adenocarcinoma cells.

**Figure 33:**
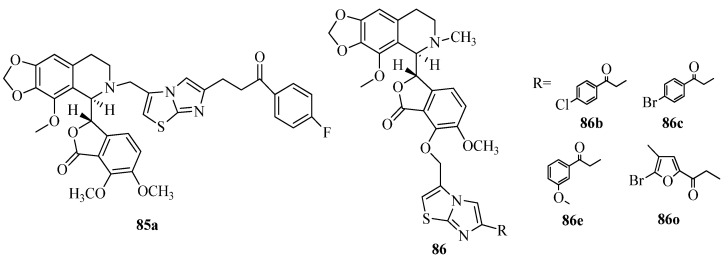
Structures of imidazo[2,1-b]thiazole-coupled noscapine derivatives.

Kantevari et al. [131] reported the synthesis of a series of imidazo[2,1-b]thiazole-coupled noscapine derivatives **85a**–**o** and **85a**–**o** (Figure 33) and their in vitro cytotoxicity screening against four tumor cell lines: DU-145 (prostate), MCF-7 (breast), SK-N-SH (neuroblastoma), and MIAPaCa-2 (pancreatic), by SRB assay. The screening revealed that among the tested compounds **85a**, **86b**, **86c**, **86e**, and **86o** were found to be active against all tested cancer cells. O-derived imidazothiazoles (**86o**, **86b**, and **86c**) displayed the highest potency with IC_50_ values of 3.6, 3.9, and 4.2 μM, respectively, whereas *N*-derived imidazothiazoles (**85a**–**o**) appeared to be less active, except for compound **86a** (IC_50_ of 4.2 ± 0.6 μM).

The presence of the bromine and methoxy group was more beneficial for O-noscapine derivatives **86o** and **86c** compared to N-noscapinoids (**86o**, **86c**). Compound **86e** showed good cell proliferation inhibition with IC_50_ of 6.9 ± 1.4μM, compared to the other compounds. The most active compounds **85a**, **86b**, **86c**, **86e**, and **86o** were also potent in the G2/M phase of the cell cycle.

**Figure 34:**
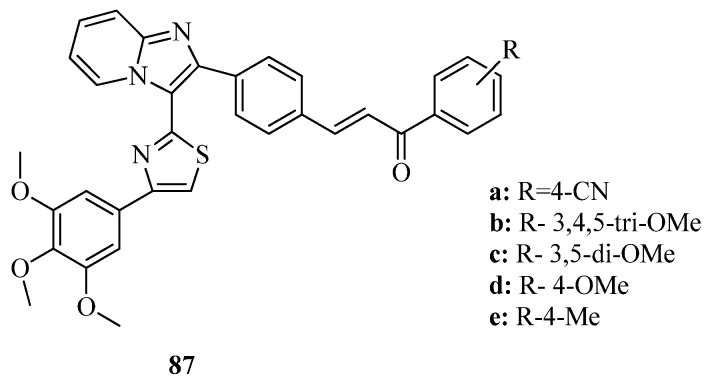
Structures of chalcone-linked thiazole-imidazopyridine derivatives.

Encouraged by the data from the literature regarding the anticancer activity of chalcones [132,133,134,135,136], Suma et al. [137] designed and synthesized a library of chalcone-linked thiazole-imidazopyridine (**87a**–**j**, Figure 34) with the purpose to study their anticancer activity. Compounds were tested on the following four human cancer cell lines MCF-7 (breast carcinoma), A549 (lung carcinoma), DU-145 (prostate carcinoma), and MDA MB-231 (breast carcinoma) using MTT assay and etoposide as positive control. The study revealed that among the ten tested compounds, five (**87a**–**e**) showed very good anticancer activity, in most cases higher or equipotent to etoposide. The highest activity was achieved for compound **87b** against MCF-7, A549, DU-145, and MDA MB-231 with IC_50_ values of 0.18, 0.66, 1.03, and 2.31 μM, respectively compared to reference drug (IC_50_ = 2.11, 3.08, 1.97, and 1.91 μM). The activity decreased from **87b** with an electron-rich (3,4,5-thrimethoxy) group on the phenyl ring to **87c** (3,5-dimethoxy) and more to **87d** with one methoxy group in position 4, indicating the positive role of the presence of an electron-rich donating group. The replacement of the 4-methoxy group with electron-withdrawing substituents (4-Cl, 4-Br, 4-NO_2_ and 3,5-di-nitro) had a negative effect, decreasing the anticancer activity even more.

In order to elucidate the potential mechanism of action of synthesized compounds, the docking studies were carried out against three potential targets, protein kinases CLK1 (5 × 8I), EGFR (2J5F), and tubulin (1SA0). The best docking score was found against CLK1 and binding energy (−9.264 to −8.794 kcal/mol) and was in accordance with anticancer activity.

**Figure 35:**
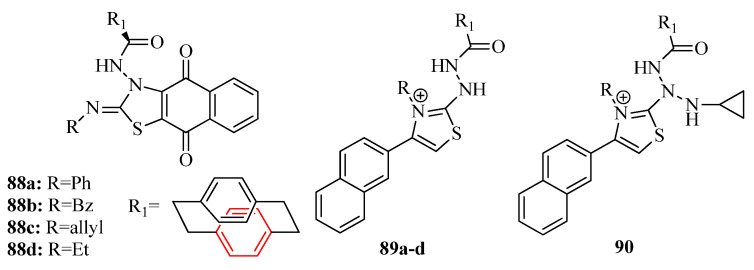
Structures of paracyclophanyl-thiazole hybrids.

Based on the data from the literature [138,139,140] as well as on their previous results [141], Aly et al. [142] designed and synthesized a novel series of derivatives incorporating three bioactive entities such as 1,4 dihydronapthoquinone, thiazole, and [2.2] paracyclophane in the frame of one molecule **88a**–**e**, **89a**–**d**, **90** (Figure 35). Compounds **88a**–**e** displayed the highest inhibition of proliferation of different cancer cell lines, showing a broad spectrum of anticancer activity against nine tumor subpanels in an in vitro five dose full NCI 60-cell panel assay. The best activity against CDK kinase with an IC_50_ of 54.8 nM was achieved for compound **88c**, which showed also the highest proliferation inhibition with IC_50_ of 0.81 μM. Its effect on caspase-3, downregulation of phosphor-Tyr15, was comparable to the dinaciclib as a reference. All compounds showed good binding affinities to CDK1 (−8.6 to −9.5 kcal/mol) compared to dinaciclib (−10.6 kcal/mol). Thus, it can be concluded that CDK1 inhibition is a probable mechanism of the anticancer activity of these compounds.

### 3.4. Thiazole Derivatives as Antiviral Agents

Viral infections ranged from self-limiting to the more serious and fatal infections. Some viral infections are of great public health importance worldwide e.g., Hepatitis B, Hepatitis C, and HIV [143]. Although antiviral nucleosides represent an attractive approach for the development of new antiviral drugs, yet the developing resistance against them limits their application. Hence, there is a need for new antiviral drugs in the fight against fatal infections.

**Figure 36:**
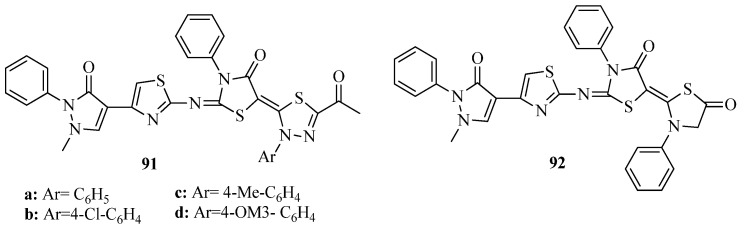
Structures of bis-1,3-thiazole derivatives.

Dawood et al. [144] reported the synthesis of new bis-1,3-thiazole derivatives and evaluation of their antiviral activity. Compounds were screened in vitro against four viruses, Hepatitis B (HBV), Hepatitis C (HCV), Influenza A virus (H1N1), and Poliovirus 3. The results of antiviral assay showed that Polio and Hepatitis B viruses were resistant against all tested compounds (EC_50_ >100 μM of each) compared to the reference drug. On the other hand, the screening against Hepatitis C virus revealed that bis-thiazole derivative **91a** demonstrated good activity against HCV virus of EC_50_ 0.56 μM. Furthermore, compounds **91b**–**d** (Figure 36) also exhibited promising antiviral activity of EC_50_ 1.12, 1.04, and 2.80 μM, respectively. Their toxicity (CC_50_ > 20 μΜ) was comparable to the reference drug ΙFNa-2b (CC_50_ >20 μM). The introduction of substituents to the phenyl ring was not beneficial, leading to less active compounds. Interestingly, compound **92**, the only one with an appreciated effect against influenza virus H1N1 (EC_50_ 24 and 23 μg/mL in visual and neutral red assays, respectively), was found to be potent also against Hepatitis C virus with EC_50_ of 12.7 μΜ.

**Figure 37:**
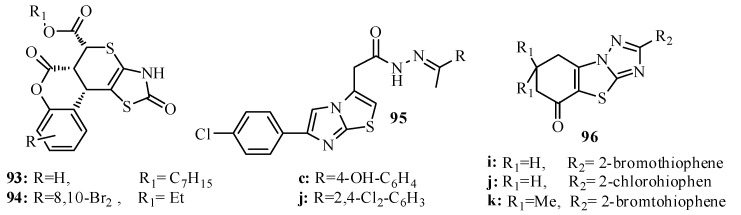
Structures of thiopyrano-[2,3-d][1,3]thiazoles, arylidenehydrazide derivatives bearing imidazo[2,1-b]thiazole moiety and imidazo[2,1-*b*]thiazole-based compounds.

Lesyk et al. [145] synthesized twenty-four thiopyrano[2,3-d][1,3]thiazole derivatives (Figure 37) and performed antiviral assay of a virus’s panel with a protocol of the NIAID’s antimicrobial acquisition [146]. According to the results of antiviral assay, some derivatives showed certain sensitivity toward influenza virus types A, H_3_N_2_, and H_5_N_1_, as well Dengue virus. The most promising group of compounds was found to be derivatives of rel-(5R,5aR,11bs)-2,6-dioxo-3,5a,6,11b-tetrahydro-2 H, 5 h-chromeno [4′,3′:4,5]thiopyrano[2,3-d][1,3]thiazole-5-carboxylic acids. Thus, compounds **93** and **94** (Figure 37) were the most active, exhibiting high activity against influenza virus type A (H_3_N_2_, Perth strain) with EC_50_ values of 0.6 ÷ 2.5 μg/mL and SI = 40.0 ÷ >170.0, and against influenza virus type A (H_5_N_1_, Vietnam strain) with EC_50_ of 0.31 ÷ 0.32 μg/mL and SI = >310.0 ÷ >320.0 respectively. In general, most of the compounds from this group showed activity against influenza virus.

Güzeldemirci et al. [79] presented the synthesis of arylidenehydrazide derivatives bearing imidazo[2,1-b]thiazole moiety (**95a**–**j**, Figure 37) and studied their antiviral activity against a panel of viruses including influenza, parainfluenza-3 virus, Feline coronavirus, coxsackie B4 virus, punta toro virus in Vero, HSV-1 (KOS), and many others. The study revealed that only two compounds demonstrated some activity. Thus, compound **95j** was found to be active against Feline coronavirus with EC_50_ of 7.5 μΜ, while **95c** was active against HSV-1(KOS) virus with EC_50_ of 9 μΜ compared to ribavirin (HCV EC_50_ of 2 μΜ).

Galochkina et al. [147] described the synthesis and biological evaluation of a series of novel imidazo[2,1-*b*] thiazole-based compounds (**96a**–**k**) as potential inhibitors of influenza virus in vitro (Figure 37). Compounds were tested against influenza virus A/Puerto Rico/8/34 (H1N1) in MDCK cells [145]. The evaluation revealed that among eleven tested compounds, only three compounds, thiophene-substituted derivatives (**96i**–**k**), exhibited activity against influenza virus with an EC_50_ in the range of 13 ± 3 to 49 ± 6 μΜ, which is better than rimantadine (EC_50_ of 72 ± 8 μΜ). The highest activity was achieved for compound **97j** with EC_50_ of 13 ± 3 μΜ and SI 77. Despite compounds **96i**–**k** having a favorable toxicity profile (CC_50_ > 200 μΜ) compared to another reference drug, oseltamivir carboxylate (EC_50_ of 0.2 ± 0.0 and SI > 1000), they were much less potent.

Compounds **96i**–**k** were tested also for their ability to inhibit influenza virus neuraminidase in luminescent MUNANA-based assay [148] in order to elucidate the mechanism of activity of this group of compounds. The obtained results did not show any correlation between the anti-neuraminidase activity of compounds and their anti-virus activity, indicating that they are not targeted against neuraminidase and act by another mechanism.

**Figure 38:**
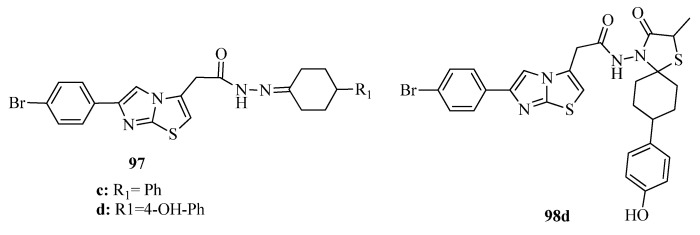
Structures of Imidazo[2,1-b] thiazole derivatives.

Gürsoy et al. [149] synthesized a series of novel acyl-hydrazone and spirothiazolidinone (**97a**–**d**, **98a**–**d**) derivatives of imidazo[2,1-b] thiazole and evaluated their antiviral activity (Figure 38). The study revealed that among all compounds tested, only compound **98d** exhibited very good activity against Feline corona virus in CRFK cells, with an EC_50_ value of 4.8 μM and SI more than 20, being much more potent than ganciclovir, used as reference drug (EC_50_ value of >100 μM). Nevertheless, it was twice less active than UDA (EC_50_ 2.4 μM), the second reference drug. On the other hand, two derivatives with acyl-hydrazone moiety **97c** and **97d** displayed weak activity against DNA viruses (HSV-1, ACV-HSV-1, HSV-2, vacinia virus) in HeLa cells with an EC value of 45 μM. From these results, it is obvious that the presence of spirothiazolidinone moiety with -4hydroxyphenyl substituent at the 8-position is beneficial for the antiviral activity against Feline coronavirus, while the presence of acyl-hydrazone moiety showed a positive effect against DNA viruses.

Compound **98d** is another hit compound, exhibiting activity against Coxsackie B4 virus with an EC_50_ value of 10 μM, being much more active than both reference drugs DS-5000 (EC_50_ of 84 μM) and Ribavirin (EC_50_ > 250 μM).

#### Thiazole Derivatives as Anti-HIV Agents

**Figure 39:**
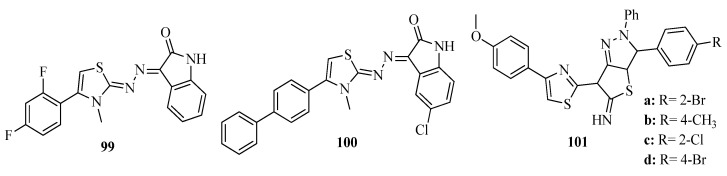
Structures of 3-3-{2-[2-3-methyl-4-phenyl-2,3-dihydro-1,3-thiazol-2-ylidene]hydrazin-1-ylidene-2,3-dihydro-1H-indol-2-one and pyrazolo[3.4,d]thiazole derivatives.

Meleddu et al. [150] synthesized a novel series of 3-3-{2-[2-3-methyl-4-phenyl-2,3-dihydro-1,3-thiazol-2-ylidene]hydrazin-1-ylidene-2,3-dihydro-1H-indol-2-one derivatives (Figure 39) and performed docking experiments into the two RT (Reverse Transcriptase) associated functions of the HIV-1 virus. The results indicate that compounds **99** and **100** are generally able to inhibit both RDDP and RNase H functions at µM concentrations and that these molecules are a good starting point for the design of new dual HIV-1 RT inhibitors.

A new series of pyrazolo [3.4,d] thiazole derivatives was synthesized and evaluated for their anti-HIV-1 activity by Kasralikar et al. [151]. The HIV-1 RT inhibition activity ranged from 60 to 94% inhibition at 100 μg/mL concentration. The most potent compounds were **101a** and **101b** (Figure 39), which showed the highest inhibitory activity (90.57%, 89.80%). In the cell-based assay, these compounds were the most potent inhibitors of HIV-1 replication against HIV-1 IIIB with EC_50_ of 0.74 and 1.08 μg/mL, respectively.

Docking studies showed that the inhibitory activity of compounds **101a** and **101b** decreased due to the absence of π–π interaction of the methoxy phenyl-thiazolyl moiety into the hydrophobic binding pocket, surrounded by the aromatic Trp229 residue and the lack of interaction with the hydrophobic binding pocket, surrounded by the residue lys101.

**Figure 40:**
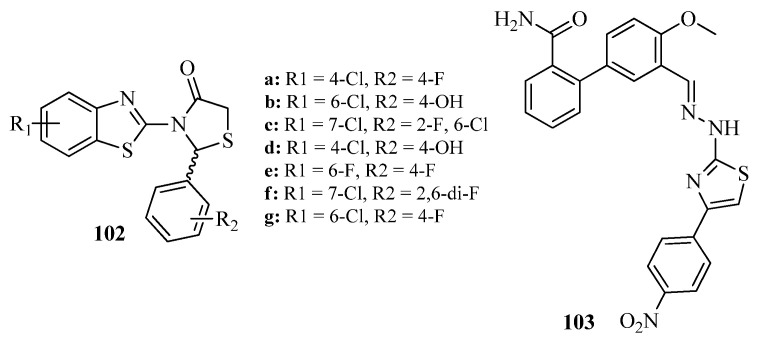
Structures of benzothiazole-based thiazolidin-4-ones and biphenylhydrazo 4-arylthiazoles derivatives.

Petrou et al. [152] took into account the best features of available NNRTIs, designed, and synthesized a novel series of benzothiazole-based thiazolidin-4-ones as potential non-nucleoside inhibitors of HIV-1 Reverse Transcriptase. The evaluation of HIV-1 RT inhibitory activity of synthesized compounds showed that seven compounds **102a**–**g** (Figure 40) had lower ΙC_50_ values than reference drug nevirapine (0.3 μΜ), while three of the compounds (**102a** ≥ **102b** > **102c**) exhibited IC_50_ values lower than 5 nM. Two compounds **102a** and **102b** exhibited very good inhibitory activity with an IC_50_ 1 nM). Moreover, the activity of compounds seemed to depend not only on the nature of substituent and its position in benzothiazole ring but also on the nature and position of substituents in the benzene ring.

Continuing their previous work, Meleddu et al. [153] synthesized a small library of biphenylhydrazo 4-arylthiazoles derivatives and evaluated them in order to investigate their ability to simultaneously inhibit both associated functions of HIV reverse transcriptase. Results revealed that all compounds were active toward the two functions. Derivative **103** (Figure 40) was the most potent with IC_50_ values of 4.5 and 8.0 μM toward RNase H and RDDP, respectively. Moreover, docking studies highlighted the relevant role of the 2-amido substituent in stabilizing the ligand/enzyme complex.

### 3.5. Thiazole Derivatives as Antidiabetic Agents

#### 3.5.1. Protein Tyrosine Phosphatase 1 B (PTP1B) Inhibitors

Protein tyrosine phosphatase 1 B (PTP1B) is an important target for the therapy of type-II diabetes. It modulates the signal pathway of insulin and leptin in a negative way, increasing the phosphorylation of insulin receptor and its substrate, glucose uptake in insulin-responsive cells and endorsing insulin action in receptive tissues. Based on the variety in the mechanisms of PTP1B inhibitors, an enormous number of novel molecules has been designed and synthesized in the past 6 years, highlighting the importance of PTP1B in the treatment of type-II diabetes.

**Figure 41:**
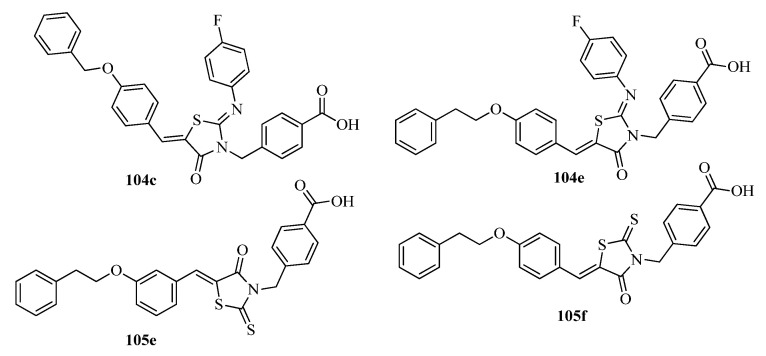
Structures of {[5-arylidene-2-(4-fluorophenylimino)-4-oxothiazolidin-3-yl]methyl}benzoic acids (5) and 2-thioxo-4-thiazolidinone analogues.

Ottana et al. [154] based on their previous research on PTP1B inhibitors [155,156,157], synthesized {[5-arylidene-2-(4-fluorophenylimino)-4-oxothiazolidin-3-yl]methyl}benzoic acids and 2-thioxo-4-thiazolidinone analogues (**104**, **105**, Figure 41) as potent PTP1B inhibitors. The most active compounds were **104c**, **104e**, and **105f** with IC_50_ values of 1.6, 1.5, and 1.9 μΜ, respectively. The SAR studies revealed that the presence of ethoxy chain between the two phenyl rings was beneficial, leading to excellent inhibitors independent of the position of this group. In the most active compound among 2-(4-fluorophenylimino)-4-thiazolidinon **104e**, 2-phenylethoxy substituent is in position 4 of the 5-benzylidene ring, whereas in the most effective inhibitor of series 2-thioxo-4-thiazolidinones (compound **105f**)), this group is in position 3. Kinetic studies revealed that the behavior of compounds **104** is mixed non-competitive, while compound **105** showed mixed type inhibition. According to docking studies in the active side, the [4-(2-phenylethoxy)phenyl]methylidene substituted compounds (**104e**, **105e**) interact with the catalytic cavity in a way similar to many published PTP1B inhibitors [158]. This substitution was involved in hydrogen bond interactions with the residue Arg221 as well as with residues Arg221 and Ser216.

**Figure 42:**
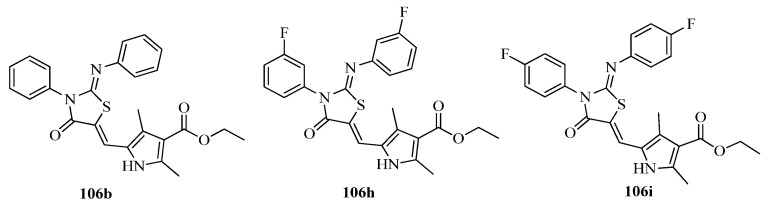
Structure of 2-imino-3-substituted 5-heteroarylidene-1,3-thiazolidine-4-one derivatives.

That same year, Meng et al. [159] synthesized a series of 2-imino-3-substituted-5- heteroarylidene-1,3-thiazolidine-4-one derivatives as possible PTP1B inhibitors. It was found that substitution by pyrrole groups at the 5-position of the 1,3-thiazolidine-4-one was more beneficial than that of the compounds with the substituted pyridine groups on the same position. Thus, compounds **106b**, **106h**, and **106i** (Figure 42) exhibited good activity against PTP1B among which compound **106i** was the most active with an IC_50_ of 6.37 μM. This compound can be considered as the lead for further modifications.

**Figure 43:**
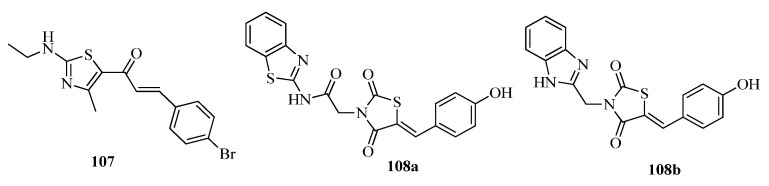
Structures of (E)-3-(4-bromophenyl)-1-(2-(ethylamino)-4-methylthiazol-5-yl)prop-2-en-1-one (**107**) and thiazolidine-2,4-dione/benzazole derivatives (**108**).

Ganou et al. [160] evaluated the PTP1B inhibitory activity of ten previously synthesized thiazole derivatives. Among all compounds, compound **107** (Figure 43) was the most active with an IC_50_ value of 18 μΜ. Molecular docking studies revealed that compound **107** can bind to the catalytic site of PTP1B with its thiazole moiety, forming a pi–pi interaction with the amino acid Phe182, while ethylamino substituted formed a hydrogen bond with residue Asp181. Structure–activity relationship studies showed that the positions and the nature of substituents in the benzene ring would affect the activity of the compounds. Compounds with the 3-bromo group on the benzene ring showed better inhibitory activity, whereas compounds with ethylamino substituents exhibited higher inhibition than the corresponding methylamino substituents.

**Figure 44:**
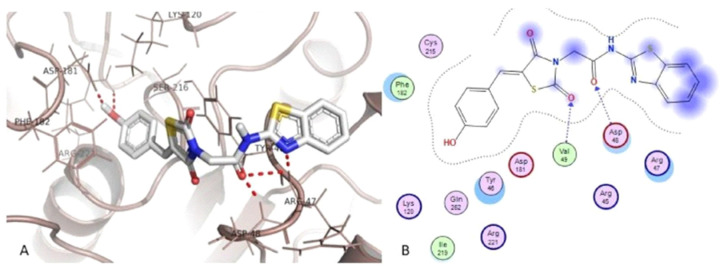
(**A**) Three-dimensional (3D) docking pose of compound **108a** bind to the catalytic site of PTP1B and simultaneously to the second aryl phosphorylate site. (**B**) Two-dimensional (2D) diagram of docking pose of compounds **108a**.

Hidalgo-Figueroa et al. [161] designed and synthesized two thiazolidine-2,4-dione/benzazole derivatives (**108a**, **108b**) as PTP1B inhibitors (Figure 43). The compounds **108a** and **108b** (K_i_ = 3.7 ± 0.5; 3.5 ± 0.4 µM) reduced the enzyme activity by 85% and 50% respectively and non-competitive inhibited the PTP1B enzyme. Compound **108a** bearing an amide spacer exhibited higher inhibitory activity against PTP1B with an IC_50_ value of 9.6 ± 0.5 μM than compound **108b**, which exhibited 50% inhibition of PTP1B at a concentration of 20 μM. Molecular docking studies showed that compound **108a** binds to the catalytic site, which consists of residues Asp181 and Arg221, and simultaneously to the second aryl phosphorylate site, which consisted of residues Arg47 and Asp48 of PTP1B (Figure 44).

**Figure 45:**
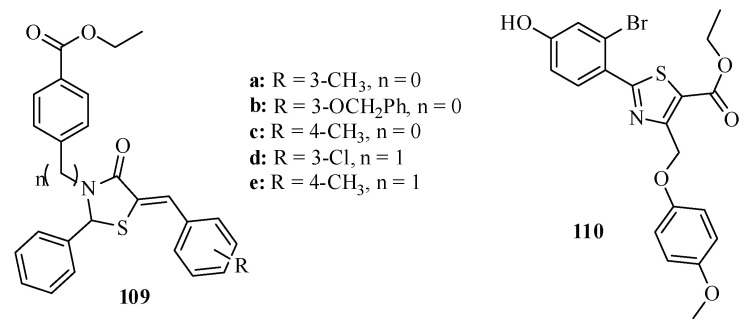
Structures of 4-thiazolinone (**109a**–**e**) and ethyl 4-(substituted phenoxymethyl) thiazole-5-carboxylate derivatives (**110**).

Liu et al. [162] reported a series of 4-thiazolinone derivatives (**109a**–**e**, Figure 45) with high PTP1B inhibitory activity (IC_50_ values < 3 μM). Compound **109e** was the most active PTP1B inhibitor with an IC_50_ value of 0.92 µM. Molecular docking studies revealed that this compound could bind to the active site of PTP1B through non-covalent interactions and can interact forming hydrogen bonds with the catalytic residues Asp181, Cys215, and Gln262.

Wu et al. [163] synthesized a class of ethyl 4-(substituted phenoxymethyl) thiazole-5-carboxylates as PTP1B inhibitors. Among all compounds, compound **110** (Figure 45) was found to have the highest inhibitory activity against PTP1B enzyme with an IC_50_ value at 4.46 μM). The structure–activity relationship showed that different substituents on the benzene ring affected the biological activity of the compounds.

**Figure 46:**
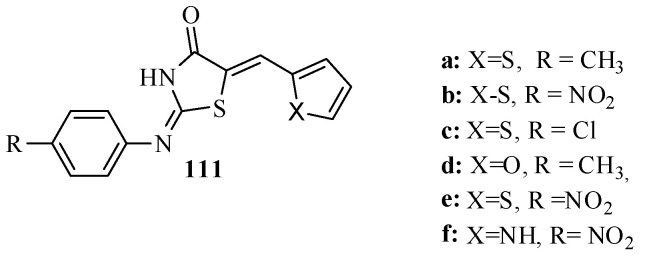
Structure of thiazolidin-4-one derivatives **111a**–**f**.

Patel et al. [164] reported the synthesis of three series of thiazolidin-4-one derivatives and evaluation of their PTP1B inhibitory activity. Among all compounds, **111a**–**f** (Figure 46) exhibited potent inhibitory activity with an IC_50_ values < 10 µM with compound **111e** being the best competitive PTP1B inhibitor with an IC_50_ value of 5.88 ± 0.06 µM. Molecular docking studies revealed that this compound is a catalytic inhibitor that can interact with the catalytic residues Cys215, Ser216, and Gln262 of PTP1B. Structure–activity relationship studies showed that the presence of a furan moiety and the introduction of a nitro group in the benzene ring can increase the PTP1B inhibitory activity of the compounds.

#### 3.5.2. Dipeptidyl Peptidase-4 (DPP-4) Inhibitors

Dipeptidyl peptidase-4 (DPP-4) is a serine exopeptidase capable of inactivating various oligopeptides through the removal of N-terminal dipeptides. The regulation of the secretion of insulin after meals is accomplished by hormones incretins. DPP-4 acts by removing incretin from the body, which is the normal metabolic process in people without diabetes. Thus, molecules acting as DPP-4 inhibitors have been identified as a worthwhile therapy for T2DM management.

**Figure 47:**
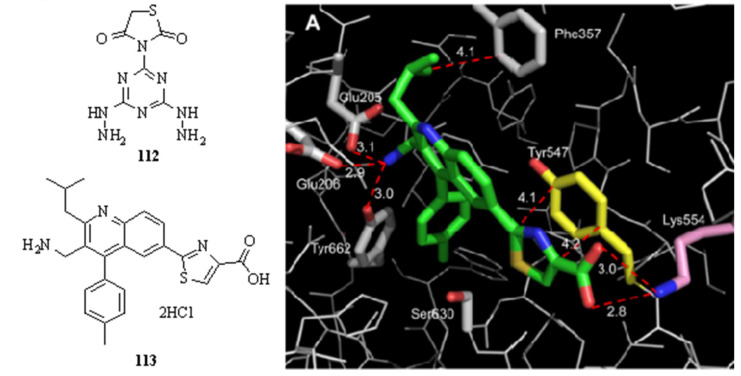
Structure of 1,3,5-triazine thiazolidine-2,4-dione **112** and thiazole based quinoline salt **113.** (A) Docking study of compound **113** (green) with DPP-4 protein. Lys554 (pink), Tyr547 (yellow), and other amino acids (gray).

Shrivastava et al. [165] reported a series of 1,3,5-triazine thiazolidine-2,4-diones analogues (Figure 47) and evaluated them as DPP-4 inhibitors. Compound **112** was the most potent with an IC_50,_ value of 6.25 μM. Molecular docking studies revealed that the amine near the wing of the triazine forms a hydrogen bond with residue Glu205, which stabilizes the complex. Moreover, structure–activity relationship studies demonstrated that bulky substituents, such as nitro- and chloro-phenyl, decrease the inhibitory activity of compounds.

Maezaki et al. [166] synthesized a series of novel thiazole-based quinoline salts as DPP-4 inhibitors. Compound **113** (Figure 47) exhibited the most potent inhibitory activity with an IC_50_ value of 0.38 nM. Molecular docking studies suggested that a hydrophobic interaction with Tyr547 as well as the salt bridge interaction is important for the extremely high activity of the compounds.

#### 3.5.3. Aldose Reductase (ALR) Inhibitors

Aldose reductase is an enzyme of the polyol pathway that alters glucose to sorbitol in the presence of nicotinamide adenine dinucleotide phosphate (NADPH), which then is converted to fructose in the presence of sorbitol dehydrogenase [167]. In type-II diabetes mellitus (T2DM) patients with poor sorbitol penetration and metabolism, an excessive accumulation of sorbitol occurs in tissues, resulting in DM-associated complications such as cataracts and glaucoma. Thus, inhibition of the aldose reductase enzyme is a promising therapy for preventing T2DM complications.

**Figure 48:**
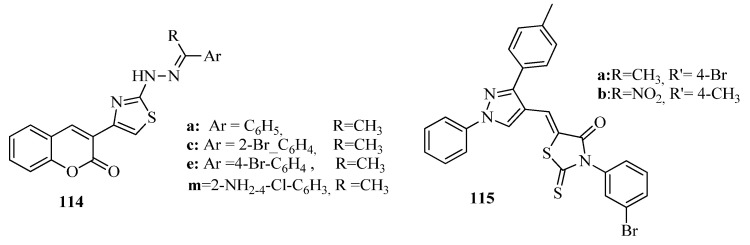
Structure of coumarin–thiazole **114** and pyrazole–rhodanine hybrids **115a**–**b**.

Ibrar et al. [168] reported the synthesis and evaluation of a novel series of coumarin-thiazole (**114a**–**o**) and oxadiazole derivatives against ALR2 and ALR1. Among all synthesized coumarin-thiazole derivatives **114a**, **114c**, **114e**, and **114b** (Figure 48) exhibited the highest activity against ALR2 with IC_50_ values at 0.12 ± 0.05, 0.16 ± 0.06, and 0.11 ± 0.001 μM, respectively, which is better than sulindac used as a reference drug (EC_50_ = 0.293 ± 0.08 μM). On the other hand, compound **114m** was the most potent against ALR1 with an IC_50_ value of 0.459 ± 0.001 μM being more than a 100 times stronger inhibitor compared to valproic acid. The structure–activity relationship of the tested compounds revealed that activity and selectivity is dependent on various substituents in the aromatic ring. Docking studies performed using X-ray structures of ALR1 and ALR2 with the given synthesized inhibitors show that the coumarinyl-thiazole series lacks the carboxylate function that could interact with the anionic binding site, which is a common ALR1/ALR2 inhibitors trait.

A series of novel pyrazole–rhodanine hybrids has been reported by Andleed and co-workers [169]. Among all compounds synthesized, two compounds **115a** and **115b** (Figure 48) were found to be potent inhibitors of ALR2 with IC_50_ values of 1.22 ± 0.67 and 2.34 ± 0.78 μM respectively, compared to the reference drug sorbinil (IC_50_ = 3.10 ± 0.20 μM). Furthermore, docking studies showed that compound **115a** binds ALR2 in a favorable way with its pyrazole ring occupying the binding pocket and interacting with residues Trp111, His110, Thr113, and Leu300. Structure–activity relationship studies revealed that the presence of methoxy, nitro, and fluoro groups at the para position of the aryl ring close to the pyrazole moiety decrease the inhibition of ALR1. However, their replacement with bromo and hydroxyl groups resulted in an increase of the inhibitory potency.

**Figure 49:**
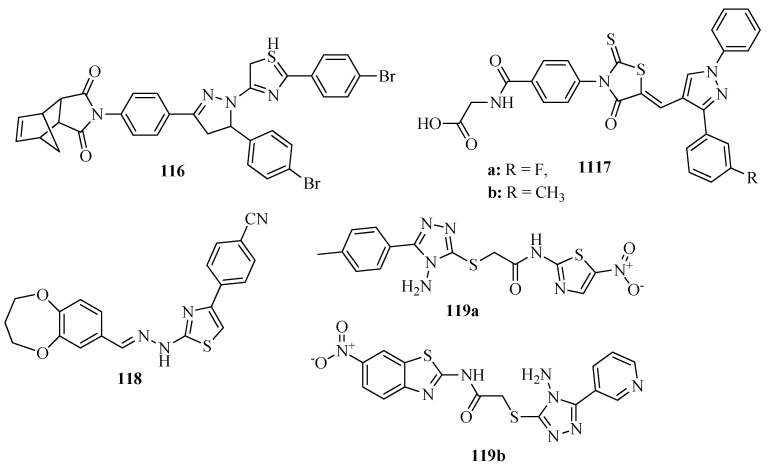
Structures of pyrazolyl–thiazole (**116**), 1,3-diarylpyrazol-rhodanine-3-hippuric acid derivatives (**117**), -aryl-2-[2-((3,4-dihydro-2*H*-1,5-benzodioxepine-7-yl) methylene) hydrazinyl] thiazole (**118**), and 2-[(4-amino-5-aryl-4*H*-1,2,4-triazol-3-yl)thio]-*N*-(thiazol/benzothiazol-2-yl)acetamides (**119a**,**b**).

The inhibitory effects of pyrazolyl–thiazoles derivatives (**116a**–**i**) (Figure 49) on AR and α-glycosidase enzymes were investigated by Demir et al. [170]. All compounds showed good AR inhibitory activity, with compound **116d** being the most potent, with a *K*_i_ value of 7.09 ± 0.19 µM.

Celestina et al. [171] synthesized a series of novel 1,3-diarylpyrazol-rhodanine-3-hippuric acid derivatives. Various substituents such as fluorine, bromine, methyl, chlorine, and nitro were added on the *meta—*or *para*—position of the phenyl ring in pyrazole’s 3-position. Among all compounds synthesized, compounds **117a** and **117b** (Figure 49) were found to be potent ALR2 inhibitors with IC_50_ values of 40 and 60 nM, respectively. Docking studies analysis revealed that hippuric acid’s chain is responsible for strong interaction with the main catalytic residues in the anionic binding pocket of the ALR2 enzyme.

Sever et al. [172] synthesized and screened new 4-aryl-2-[2-((3,4-dihydro-2*H*-1,5-benzodioxepine-7-yl) methylene) hydrazinyl] thiazole derivatives for their inhibitory effects on AR. The results showed that all compounds were promising AR inhibitors with the K_i_ values in the range of 0.018 ± 0.005 to 3.746 ± 1.321 μM compared to the reference drug quercetin (K_i_ = 7.025 ± 1.780 μM). 4-(4-Cyanophenyl)-2[2-((3,4-dihydro-2*H*-1,5-benzodioxepin-7-yl) methylene) hydrazinyl] thiazole (**118,** Figure 49) was detected as the most potential AR competitive inhibitor with the K_i_ value of 0.018 ± 0.005 µM. Moreover, molecular docking studies of the compounds into the active site of AR revealed that the thiazole scaffold was interacting through π–π stacking interactions with residues Trp20 and Phe122 (Figure 48).

In continuation of their work, Sever et al. [173] synthesized novel 2-[(4-amino-5-aryl-4*H*-1,2,4-triazol-3-yl)thio]-*N*-(thiazol/benzothiazol-2-yl)acetamides and evaluated them for their inhibitory activities on AR. Compounds **119a** and **119b** (Figure 49). were identified as the most potent AR inhibitors with K_i_ values of 0.04 ± 0.01 µM and 0.08 ± 0.02 µM, respectively as compared to the reference drug quercetin (K_i_ = 5.66 ± 0.66 μM). These two compounds displayed competitive AR inhibition and were found to be nontoxic against healthy cells. In addition, molecular docking studies were in agreement with the biological data.

### 3.6. Neglected Diseases

Neglected tropical diseases (NTD) are the most widespread infections of the poor people in developing regions of sub-Saharan Africa, Asia, and tropical regions of America. Among these diseases is malaria, which is a female mosquito-borne infectious disease that affects humans and animals, being the most lethal human parasitic infection [174].

On the other hand, Leishmaniasis is a disease caused by the protozoan parasite of the genus *Leishmania*, which is transmitted to the mammalian hosts by the bite of the phlebotomies and fly. Leishmaniasis is a significant health problem and causes morbidity and mortality in many regions of the world [175,176,177,178].

Another tropical disease is American Trypanosomiasis or Chagas diseases caused by *T. cruzi*. Six to seven million people worldwide are infected with Chagas disease and found mainly in endemic areas of 21 continental Latin American countries. This group of diseases also includes the Human African trypanosomiasis (sleeping sickness), affecting the population in sub-Saharan Africa caused by the parasites *Trypanosoma brucei gambiense* and *Trypanosoma brucei rhodesiense* and transmitted by the tsetse fly. About 60 million people remain at risk of infection. Schistosomiasis is another neglected disease caused by a trematode of the genus *Schistosoma* that affects over 200 million people worldwide [179]. All of the above-mentioned make clear the necessity for new, efficacious, potent, and safe drugs against all these diseases.

**Figure 50:**
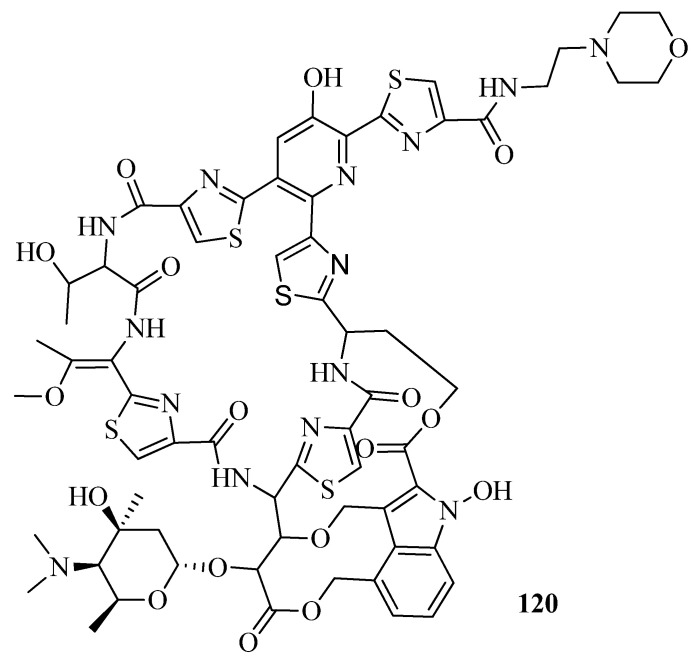
Structure of Nocathiacin I Antibiotics.

Sharma et al. [180] evaluated the potential antimalarial activity of two water-soluble derivatives of nocathiacin against the asexual blood stages of *Plasmodium falciparum*. The bactericidal activity of these derivatives against MRSA and other multidrug-resistant Gram-positive bacterial strains is comparable to that of vancomycin, the only drug for the treatment of MRSA [181,182]. For the evaluation of antiplasmodium activity of nocathiacin derivative **120** (Figure 50) against CQ-resistant parasites, the *P. falciparum* Dd2 and *P. falciparum* K1 strains were used. The IC_50_ values of **120** against these strains were at 85.67 and 99.44 nM, respectively. Derivative **120** also expresses a deleterious effect on gametocytes at a concentration of 1 μM. It should be mentioned that the parasiticidal activity of **120** against *P. falciparum* was 170-fold and 20-fold higher compared to those of thiostrepton and its derivatives.

**Figure 51:**
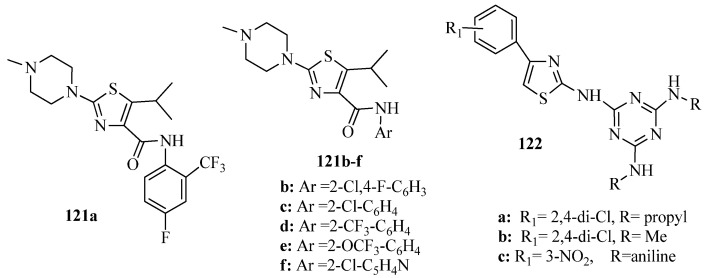
Structures of 5-isopropyl-2-(4-methylpiperazin-1-yl)-N-phenylthiazole-4-carboxamides and thiazole-1,3,5-triazines.

Bueno et al. [183] starting from the hit compound **121a** (Figure 51) from the Tres Cantos Antimalarial Set (TCAMS) synthesized a series of analogs, studied their structure–activity relationships, and evaluated their in vitro activities against the 3D7 strain of *P. falciparum*. The SAR revealed that ring B does not affect much the activity. The only condition is that position 2 of the thiazole should be directly bound by a nitrogen atom from a piperidine, piperazine, or N-methylpiperazine moiety. On the other hand, the benzene ring is the most important for the activity of these series of compounds. In addition, the presence of an isopropyl group in position 5 of the thiazole ring is very important for the antimalarial activity. The study revealed that the most potent compounds against *P. falciparum* were found to be compounds with an electron attractive group in position 2 of the benzene ring and hydrogen or F in position 4. Thus, compounds **121b**–**f** displayed very good activity with IC_50_ in the range of 0.031–0.08 μM in comparison with hit compound **1** (IC_50_ 0.024 μM). The best activity was achieved for compound **121d** with 2-CF_3_ substitution of the benzene ring. Replacement of the benzene ring by pyridine decreased the activity (IC_50_ = 0.08 μM).

Sahu et al. [184] designed the library of 378 thiazole-1,3,5-triazines and subjected them to ADME analysis. Twenty selected compounds were synthesized and evaluated by docking against wild and mutant Pf- DHFR complex, as well as by in vitro antimalarial screening. The antimalarial activities of the synthesized compounds were determined as percentage of dead rings, trophozoites, and schizonts followed by an IC_50_ determination. The study revealed that compounds **122a**–**b** (Figure 51) exhibited the highest percentage of dead rings, schizonts, and trophozoites against 3D (90, 100, and 97.5%) with an IC_50_ of 12.48, 10.03, and 11.34 μg/mL, respectively, and also against chloroquine resistant Dd2 strain (57.67 and 16%) with an IC_50_ of 15.73, 11.29, and 12.55 μg/mL respectively. Chloroquinone was used as reference drug (IC_50_ = 0.7 and 1.2 μg/mL, respectively).

The docking studies of compounds performed in the binding pocket of both the wild type (1j3i.pdb) and quadruple mutant (1j3k.pdb) pf-DHFR revealed that compound **122b** demonstrated the lowest binding energy. It formed strong H-bond interactions with Asp54 with one weak pi-cationic interaction with Phe58 in both wild and quadruple mutant pf-DHFR. The second compound with low binding energy, **122c**, showed pi–pi interactions with Phe116 and pi–sigma interaction with Ile14 of 1j3i, while with 1j3k, it formed pi–pi and H-bond interactions with Phe116, Ser111, and Arg59. As far as compound **122a** is concerned, it demonstrated a pi–cationic interaction with Phe58 and H-bonding with Ser 111 in both the wild and mutant forms of Pf-DHFR.

**Figure 52:**
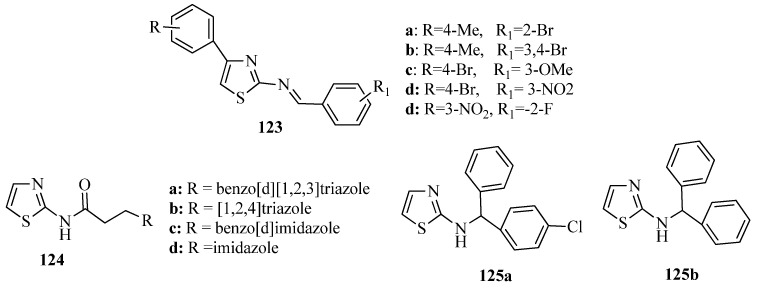
Structures of aminothiazole derivatives.

Brito et al. [185], based on previous studies on the leishmanicidal activity of 1,3-thiazole derivatives [186] and that phthalimide–thiazole derivatives showed higher affinity to *L. infantum* FeSOD than human CuZnSOD [187], designed a series of 2,4 substituted thiazole derivatives in order to evaluate their LbSOD inhibitory activity. Compounds were tested using thermal shift assay (TSA) as well as fluorescent protein-labeled assays (FLPA). TSA showed that **123c**, **123d**, and **123e** stabilize LbSOD (Figure 52). Nevertheless, a significant effect over LbSOD thermal stability (*p* < 0.05) was observed only for **123a** and **123b**. The last compound exhibited low-micromolar affinity to the macromolecular target (Kd = 4.8 ± 12.5 μM). These two compounds, having 4-nitrophenyl moiety in the position 4 of the thiazole ring, were found to increase the fluorescent signal in FPLA. Furthermore, TSA and FPLA revealed that **123b** has a low-micromolar affinity to *L. braziliensis* superoxide dismutase. The label-free methods proved the binding of **123b** to LbSOD.

Based on the data from the literature regarding significant health problems causing by leismaniasis [178,179,188] as well as the activity of the thiazole derivatives as antileishmanial agents [189,190,191], Rezai et al. [192] synthesized eight new aminothiazole derivatives and tested them for their activities against cutaneous leishmaniasis induced by *L. major* in BALB/c mice.

Based on the data from the literature that many heterocyclic five-member derivatives such as imidazole, triazole, thiazole, and furan derivatives could be potent inhibitors of 14-α-demethylase [193], a target in the drug discovery and development against leishmania infection, docking studies were also performed.

The evaluation of antileishmanial activity revealed that compounds with 1,2,4-triazole moiety (**124a** and **124b**, Figure 52) showed better antileishmanial activity, with the percentage of reducing mean area of the lesion being 86.59% and 78.24% compared with compounds **124c** and **124d** with imidazole moiety (48.94% and 55.84%). Among compounds with an amine group of aminothiazole linked to two or three phenyl rings, the most active were found to be compounds **125a** and **125b** with two phenyl groups. Compound **125a** with chloro substitution in the benzene ring showed the best activity. Docking study revealed that all tested compounds can bind to the 14a-demethylas active site from Leishmania with docking energy ranging from −7.9 to −9.7 kcal/mol. Compound **125a** showed the best docking energy (−9.7 kcal/mol) as well as the best anti-leishmanial activity. Taking into account the obtained results, someone can conclude that synthesized aminothiazole and amidinothiazole derivatives could be useful in the treatment of cutaneous leishmaniasis.

**Figure 53:**
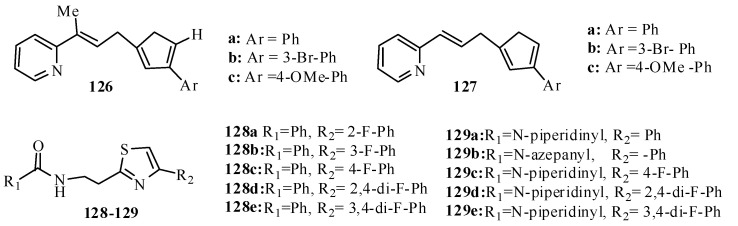
Structures of thiazoacetylpyridines, thiazopyridines (**126**, **127**) amide and urea derivatives of thiazol-2-ethylamines (**128**, **129**).

Based on the data from the literature regarding activity of thiazole-derived compounds against trypanosomatid parasites [194,195], de Oliveira et al. [196] synthesized ten thiazoacetylpyridines and thiazopyridines in order to evaluate their leishmanicidal activity (Figure 53) on *L. infantum* promastigote parasites. It was found that they inhibited promastigote growth. Nevertheless, the highest activity among thiazoacetylpyridines was shown by compounds **126a**–**c** with promastigote IC_50_ 3.57 ± 0.95, 3.12 ± 0.86, and 0.42 ± 0.03 μΜ and selectivity index of 12.73, 18.94, and 7.07. All compounds were of low cytotoxicity. Among the TP series, only **127c** showed antileishmanial activity with promastigote C_50_ = 1.35 ± 0.19. The most active compounds were also tested for their effect on intracellular amastigote. Their amastigote an IC_50_ (0.99 ± 0.03, 0.43 ± 0.11, and 0.59 ± 0.09 μΜ) was much lower and the amastigote selectivity index was higher (46.10, 137.37, and 59.05, respectively). The best activity as can be seen from the obtained result was exhibited by compound **127b**. Its SI is seven times higher than the minimum recognized for a promising drug [197].

Although the study demonstrated a significant reduction of peritoneal exudate cells (PECs) infected by *L. infantuma* and treated with tested derivatives, no significant increase in NO-infected macrophages as well as macrophages treated with compounds was observed. Finally, it can be concluded that these compounds are promising antileishmanial agents.

Except for malaria and leishmaniasis, there are also some other diseases in the group of neglected diseases. One of them is human African trypanosomiasis (HAT), which is spread mostly in sub-Saharan Africa. The origin of this disease, transmitted by tsetse flies, is chronic infection of *Trypanosoma brucei gambiense* (in western and Central Africa or an acute infection of *Trypanosoma brucei rhodesiense*, which occur in southern and eastern Africa, making up 98% of the reported infections.

Thus, Patrick et al. [197] synthesized 73 amide and urea derivatives of thiazol-2-ethylamines and studied their trypanocidic activity against *Trypanosoma brucei rhodesiense* as well as their cytotoxicity. The selectivity index (SI) was determined also. The study revealed that all compounds showed trypanocidic activity but to different extents. Halogenated compounds (**128a**–**e**, Figure 53) showed enhanced activity, with the greatest enhancements found with the fluorinated derivatives. Thus, 2-fluorophenyl derivative **128a** with 2-F with an IC_50_ of 156 nM was the best, followed by 4-fluoro (**128c**, IC_50_ 218 nM) and 3-fluoro (**128b**, IC_50_ 233 nM). The 2,4diflluoro (**128d**) and 3,4-difluoro (**128e**) exhibited similar activity with 2-fluoro derivatives. Trisubstituted urea’s derivatives showed the greatest increase of activity. The highest activity among them was achieved for compound **129a** (IC_50_, 20.4 nM) followed by compound **129b** with an IC_50_ of 51.6 nM. Finally, modification at the both ends of the molecule led to the most active compounds. The best activity was shown by compounds **129c**–**e** with IC_50_ values of 12, 9.9, and 9.7 nM, respectively and SI 10,200, 9960, and 11,700 respectively. These compounds and some others were found to be more potent than reference drugs pentamidine and melarsoprol (IC_50_ 2.8 and 4.0 μM). Nevertheless, no one of the 33 most active compounds with an IC_50_ less than 0.2 mg/mL chosen for the in vivo experiments on mice was able to cure them. In addition, their metabolic stability was poor. Some more modifications are planned in order to increase the metabolic stability of these compounds.

Schadich et al. [198], encouraged by the data from the literature regarding the antitrypanosomal activity of some thiazole-based compounds against *Trypanosoma brucei* and *Trypanosoma gambiense* [199], decided to test previously synthesized thiazolidinone and thiazole-based compounds [200,201] for their antileishmanial activity against *Leishmania major.*

**Figure 54:**
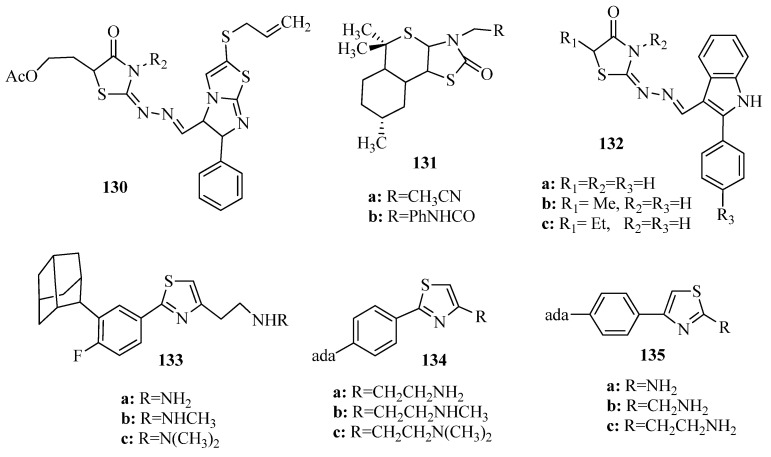
Structures of thiazole-based derivatives (**130**–**132**) and 2,4-disubstituted arylthiazoles (**133**–**135**).

The screen of 95 compounds from the library of previously synthesized compounds against promastigotes of *L. major* Friedlin V1 (FV1) strain demonstrated that 29 compounds at a concentration of 50 μM inhibited the growth of *L. major* FV1 promastigotes in a good rate (>50%). Among them, the thiazole derivatives showed good activity with IC_50_ values in range of 8.68–28.84 μM. The highest activity among them was achieved for compounds **130** and **131a**–**b** (Figure 54) with IC_50_ = 8.94 ± 1.2, 9.81 ± 1.11, and 8.68 ± 1,34 μM respectively compared to reference drug miltefosine (IC_50_ = 8.18 ± 1.57 μM). Nevertheless, thiazolidinone derivatives **132a**–**c** exhibited the best activity among all compounds with IC_50_ values in range of 1.01 ± 0.13–1.73 ± 0.22 μM. According to the obtained results, these compounds could be a scaffold for the development of novel antiparasitic drugs.

Georgiadis et al. [202] reported the synthesis of 27 new 2,4-disubstituted arylthiazoles with the purpose to study their activity against the bloodstream form *Trypanosoma brucei*. The evaluation of trypanocidal potency revealed that compounds **133a**–**c** (Figure 54) exhibited the highest activity among 2,4 disubstituted arylthiazoles with IC_50_ values of 0.42 ± 0.01, 0.90 ± 0.01, and 0.79 ± 0.02μΜ, respectively. Compounds **134a**–**c** also showed a prominent pharmacological profile with IC_50_ values of 0.80 ± 0.03, 0.59 ± 0.02, and 1.27 ± 0.07 μΜ respectively. Furthermore, the 2-phenylthiazol-4-ethylamines **133a**, **133c**, and **134a**, **134c**, were found to be more active than their isomeric 4-phenylthiazol-2-ethylamines **135c** and **135d** (IC_50_ 2.74 ± 0.29 μΜ and 1.41 ± 0.09 μΜ). It was observed that bulkier substituents than the methyl group on amine are not favorable for the trypanocidal activity. The presence of cyano and azido substituents at position 2 of the thiazole ring decreased trypanocidal activity, while the presence of a methylamino group (**135b**) at the same position (series 3) is more preferable than an amino group (**135a**, IC_50_ = 22.5 ± 0.6 μΜ). Moreover, even though the primary amine **134a** is less potent than its congener **133a**, it showed higher selectivity, indicating a promising perspective for the design of novel trypanocidals.

**Figure 55:**
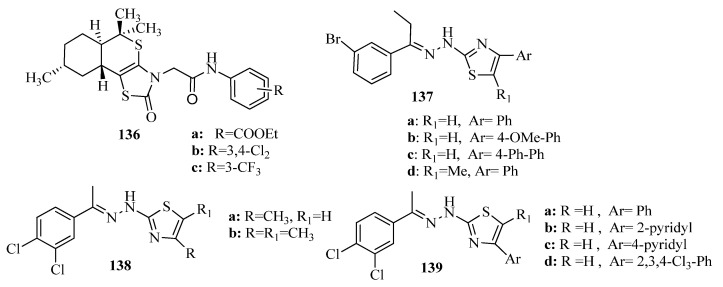
Structures of isothiochromeno[4a,4-d][1,3]thiazole derivatives (**136**), thiazolyl hydrazones.

Lesyk et al. [203] synthesized a series of isothiochromeno [4a,4-d][1,3] thiazole derivatives (Figure 55) and investigated them in an in vitro assay against *Trypanosoma brucei brucei*. Almost all compounds were found to be able to inhibit the growth of *Trypanosoma brucei* bloodstream forms with an IC_50_ range of 1.55–44.41 μM compared to reference drug Nifudimox (IC_50_ = 2.39 μM). The best activity higher than that of reference drug was achieved for compound **136a** with an IC_50_ of 1.55 μM, indicating the beneficial role of an ester group as a substituent at the N3 position of the thiazolidine core. The presence of halogen was also endowed with an increase of activity (**136b**–**c**) with an IC_50_ ranging from 2.97 to 8.50 μM, showing almost the same level of IC_50_ with the previously tested and structurally related thiopyranothiazoles with a norbornane moiety and the same fragments in the N3 position of the thiazolidine core [204]. The most active compounds were not very toxic with LD_50_ in the range of 240–480 mg/kg. Thus, compounds **136a**, **136b** (Figure 55) (IC_50_ = 4.67 μM), and **136c** (IC_50_ = 2.97 μM) can be used for further modifications with the aim of developing more potent and safer antitrypanosomal agents.

Promising results achieved by compounds bearing a 1,3-thiazole ring encouraged Gomez et al. [205] to explore the trypanocidal activity of novel thiazolyl hydrazones synthesized from 3-(bromopropiophenone) thiosemicarbazone [206]. Thus, they synthesized 3-(bromopropiophenone) hydrazinyl–1,3-thiazole derivatives and investigated their antiparasitic activity against the trypomastigote form of *T. cruzi* using benznidazole (Bzd) as a reference drug. Compounds **137a**–**d** (Figure 55) were found to be the most potent with IC_50_ values at 5.51, 3.64, 3.35, and 4.79 μM, respectively, being more potent than Bzd (IC_50_ 6.26 μM). Compound **137a** showed the same selectivity index (cytotoxicity/IC_50_) with Bzd. In order to elucidate the biological target, compounds were tested against the enzyme cruzain of the *T. cruzi*. Compound **137d** displayed good potency with an IC_50_ = 0.51 ± 0.02 μM. Interestingly, some of the most potent against *T. cruzi* compounds did not inhibit enzyme cruzain. The derivatives **137b** and **137c** exhibited improved selectivity for parasites when compared to the reference drug benznidazole. In order to understand a mechanism of cruzin, an inhibition docking study was performed. The score values for compounds **137a** and **137d** were −8.54 and −8.24 kcal/mol, respectively, which is almost in accordance with the experimental results. Compound **137d** formed a p-p T shaped interaction as well as a hydrogen bond with the residue CYS25. In conclusion, the 1,3-thiazoles core was found to be important as a building block for lead generation.

Filho et al. [207] reported the synthesis of a new series of 1,3-thiazoles (Figure 54) and evaluation of their anti-*T. cruzi* activity. Evaluation of cytotoxicity in macrophages revealed that most compounds (**138a**–**b** and **139b**–**d,** Figure 55) were toxic in concentrations less than 50 μM with four of them (**138a**, **139b**–**d**) being toxic in the same concentration for H9c2 rat cardiomyoblast cells. The most active among them against *T-cruzi* was found to be compound 148b with an IC_50_ = 0.37 μM, exhibiting higher potency than gentian violet and benznidazole, used as reference drugs (IC_50_ 0.45 and 10.61 μM, respectively. The second-best compound was **139d** (IC_50_ = 6.0 μM) with all other compounds (**138a**–**b**, **139a**, 148c) being equipotent or comparable with benznidazole.

The highest activity against cruzian enzyme was achieved for compounds **138a**–**b** with IC_50_ values of 0.6 and 9 μM. The most active compound **139b** among all did not inhibit the cruzain, suggesting that killing the parasite acts on another target. Authors showed that compound **139b** kills the parasite due to apoptosis induction.

**Figure 56:**
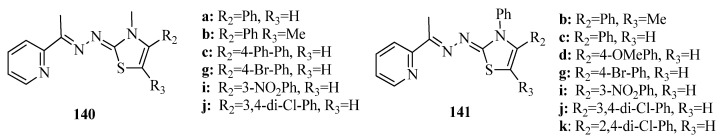
Structures of the active 2-(pyridin-2-yl)-1,3-thiazoles.

Da Silva et al. [208] synthesized 2-(pyridin-2-yl)-1,3-thiazoles (**140a**–**m** and **141a**–**m**, (Figure 56) and studied their antiparasitic and host cell cytotoxicity (Figure 56). Primarily, the ability of compounds to inhibit the epimastigote proliferation of *T. cruzi* Dm28 strain, as well as their toxicity against Y strain trypomastigotes, was determined. As reference, compound benznidazole was used (CC_50_ value of 6.2 μM against trypomastigots). The study revealed that the highest activity, better than that of the reference drug, was expressed by compounds **140b** (CC_50_ 2.7 μM), **140c** (CC_50_ 2.2 μM), **140g** (CC_50_ 2.2 μM), **140i** (CC_50_ 2.4 μM), and **140j** (CC_50_ 2.3 μM) with low cytotoxicity (CC50 of 6-36 μM), being less toxic than benznidazole (CC_50_ 44.7 μM) except for compound **140b**. As far as compounds of series **141a**–**m** are concerned, all compounds except for **141a** exhibited activity against trypomastigots higher than reference drug with CC_50_ values ranging from 1.2 to 3.1 μM. The best activity was achieved for compound **141d** (CC_50_ of 1.2 μM) followed by **141k** (CC_50_ of 1.6 μM). The evaluation of antiproliferative activity against epimastigotes demonstrated that almost all compounds were more potent than the reference drug. Only compounds **140a**–**b** and **141e**–**d** showed inhibitory activity against the enzyme cruzain (70 and 73% and 58, 48% respectively). According to these results, it seems that cruzain inhibition is not the mechanism of action of the trypanocidal compounds. The structure–activity relationships study revealed that the presence of halogen is beneficial for trypanocidal activity. The introduction of a second chlorine (**140j**) increases its activity 3-fold compared to the reference drug. In addition, it was found that its activity depends not only on substituents but also on position 1 of the ring. Thus, the 3-nitrophenyl derivative (**140i**) showed higher activity (3 times) compared to the 4-nitrophenyl one (**140h**).

In conclusion, one can say that the studied compounds are novel potent and selective antiparasitic agents and can be a starting point for the further development of new antiparasitic agents.

**Figure 57:**
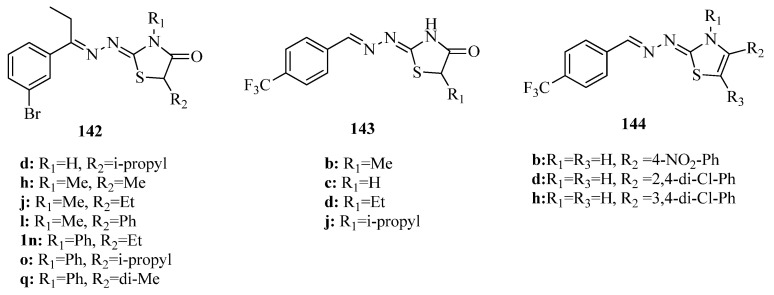
Structure of 4-thiazolidinone and 1,3-thiazole derivatives.

De Sigueira et al. [209] synthesized novel 4-thiazolidinone and 1,3-thiazole derivatives aiming to study their activity against *T. cruzi* (Figure 57). All compounds were evaluated in vitro for epimastigote, trypomastigote, and amastigote anti-*T. cruzi* activity as well as their toxicity. The study demonstrated that compounds **142j** and **142o** (IC_50_ of 46 and 42.2 μΜ respectively) were found to be equipotent with the reference drug benznidazole (IC_50_ = 48.8 μΜ), while compound **142q** (IC_50_ = 12.4 μΜ) showed higher activity than the reference drug. As far as trypanocidal activity is concerned, the best activity was achieved for compounds **142d**, **142l**, **142n**, and **143d** with an IC_50_ ranging from 3.7 to 8.1 μΜ. Among them, the most promising compound is **142n** (IC_50_ = 3.7 μΜ). As per activity in amastigote, the most active were thiazolidinone derivatives **142g** (IC_50_ 12.2 μΜ) and **142h** with an IC_50_ 2.4 μΜ. In the series of compounds **143**, compounds **143b**–**e** and **143j** were found to be active against trypomastigote form with an IC_50_ in the range of 7.8-61.9 μΜ, with the best activity showing by compound **143d.**

In the series of 1,3-thiazole derivatives, three compounds **144b** (IC_50_ 9.9 μΜ), **144d** (5.3 μΜ), and **144h** (8.9 μΜ) (Figure 57) were the most potent against amastigote form but with low selectivity. It should be mentioned that all these compounds had an electronegative group attached to the thiazole ring. The presence of a dichloro substituent in the aromatic ring of compounds **144d** and **144h** indicates their positive role for anti-*T. cruzi* activity.

### 3.7. Thiazoles as Antiflammatory Agents

Inflammation is the response of organisms to various stimuli. Many diseases such as arthritis, asthma, and psoriasis, requiring prolonged or repeated treatment, deal with inflammation. Non-steroidal anti-inflammatory drugs (NSAIDs) used in the treatment of acute and chronic inflammation, pain, and fever are the main therapeutic agents for the treatment of inflammatory diseases, being the most widely used drugs. On the other hand, their long-term clinical use is associated with significant side effects, such as gastrointestinal (GI) problems [210,211], adverse cardiovascular events [212], kidney disease [213,214], bleeding, and nephrotoxicity [211,215]. Therapeutic options investigated for inflammation control include the neutralization of tumor-necrosis factor (TNF), blockers of leukotriene receptors, inhibitors of cytokines or leukotriene synthesis, and other principal components of the inflammatory response for systemic conditions. However, cyclooxygenase (COX), the enzyme involved in the first step of the conversion of arachidonic acid to prostaglandins (PGs), remains the main target.

Classical NSAIDs, such as indomethacin, inhibit both isoforms of COX-1, which is constitutively expressed in most tissues and organs and catalyzes the synthesis of PGs involved in the regulation of physiological cellular activities; COX-2 is mainly induced by several stimuli such as cytokines, mitogens, and endotoxins in inflammatory sites [215,216]. Thus, their therapeutically effects are mainly due to the decrease of pro-inflammatory PGs produced by COX-2, whereas their unwanted side effects result from the inhibition of constitutive COX-1 isoform.

**Figure 58:**
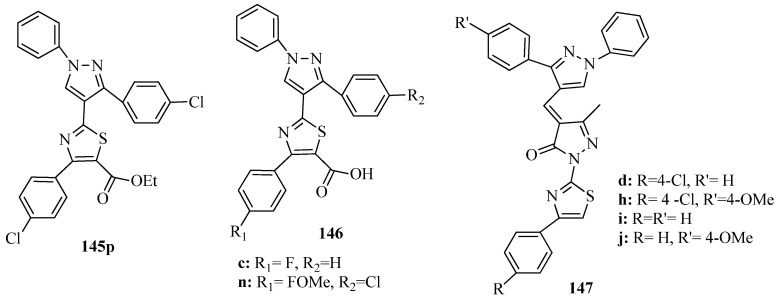
Structure of pyrazolylthiazolecarboxylates (**145**, **146**) and thiazole-bearing pyrazole derivatives.

Khloya et al. [217] reported on the design and synthesis of a novel series of pyrazolylthiazolecarboxylates **145a**–**p** (Figure 58) and corresponding acid derivatives **146a**–**p**. and in vivo evaluation of anti-inflammatory activity by carrageenan-induced rat paw edema method [218] The most active compounds were **146p**, **146c**, and **146n** with inhibition ranging from 93.06 to 89.59%. It seems that ester derivatives are more potent than acid ones.

Finally, it seems that these compounds are a promising scaffold for designing anti-inflammatory agents. It seems that ester derivatives are more potent than acids.

Kamble et al. [219] searching for novel, safe, and effective anti-inflammatory agents synthesized thiazole-bearing pyrazole derivatives **147a**–**l** (Figure 58) and their anti-inflammatory as well as COX-1/COX-2 inhibitory activities. Two derivatives **147e** and **147h** showed the highest protection of inflammation induced by carrageenan after 3 h (87.75% and 92.85%, respectively). Moreover, compound **147h** exhibited good protection even after 90 min (more than 80%) compared to diclofenac used as reference drug (95.45%). On the other hand, evaluation of COX-1/COX-2 inhibitory activity revealed that compounds **147h**, **147i**, **147d**, and **147j** appeared to be good COX-2 inhibitors with inhibition values of 78.91–61.84% (Figure 58). It seems that the presence of an electron-donating phenyl substituent on pyrazole moiety is beneficial for COX-2 inhibitory activity, enhancing it, while the presence of an electron withdrawal phenyl substituent on thiazole ring has a negative effect, highlighting the importance of the electron-donating phenyl substituent on the pyrazole nucleus as a possible pharmacophore for selective COX-2 inhibitors.

**Figure 59:**
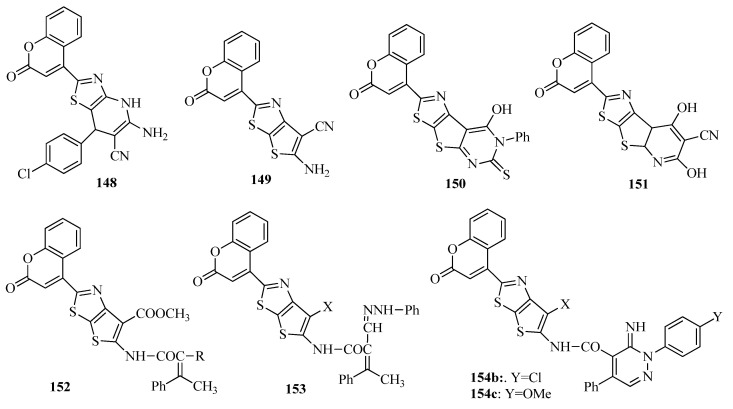
Structure of fused thiazole derivatives from 2-(2-oxo-2*H*-chromen-3-yl)thiazol-4(5*H*)-one.

Mohareb et al. [220] reported a synthesis of eleven series of thiazole derivatives and evaluation of their anti-inflammatory activity, using a carrageenan-induced paw edema model [218]. All synthesized compounds showed anti-inflammatory activity in the range of 33–96%. Compounds **148**–**153**, **154b**, and **154c** (Figure 59) were found to be the most potent inhibitors of edema, exhibiting high anti-inflammatory activity (81–96%). The best activity among all mentioned compounds was achieved for compound **150** with 96% inhibition followed by **153** and **154b** with 94% and 92% of edema inhibition, respectively. For 4,7-dihydrothiazolo[4,5-*b*]pyridine derivatives as well as pyridazine derivatives, the beneficial for activity was the presence of 4-chlorophenyl moiety (**148**), while for the thieno[3,2-*d*]thiazole derivatives, the presence of the 3-cyano group (**149**) was more favorable than the presence of the COOEt moiety (**149**). For the thiazolo[4′,5′:4,5]thieno[2,3-*d*]pyrimidine derivatives, a positive effect was observed with the presence of the hydroxyl group (**150**), while for arylhydrazone derivatives, the unsubstituted derivatives showed the best activity. Compounds **148**, **150**, **154b**, and **154c** demonstrated also high antiulcer activity.

**Figure 60:**
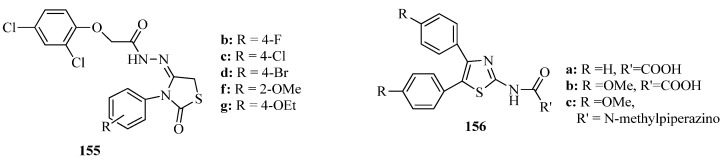
Structures of 2-imino-4-thiazolidinone derivatives and 4,5-diphenylthiazoles.

Alam et al. [221] synthesized fourteen 2-imino-4-thiazolidinone derivatives (**155a**–**n**, Figure 60) and evaluated them for in vivo anti-inflammatory activity and effect on ex-vivo COX-2 and TNF–a expression. As reference drugs, indomethacin and celecoxib were used. Compounds **155f** and **155g** showed 81.14**%** and 78.8% inhibition, respectively, which was higher than indomethacin and celecoxib with 76.1% and 77.67%, respectively, after 5 h. On the other hand, compounds **155b** (72.22%), **155d** (68.12%), **155e** (68.02%), and **155a** (64.00%) exhibited activity comparable to indomethacin. The best activity against COX-2 was achieved for compound **155f** with 68.3% inhibition, which was higher than indomethacin (66.23%) but less than celecoxib (72.96%). Compounds **155b**, **155d**, and **155f** with selectivity indexes of 14.06, 16.32, and 29.00 can be considered as selective COX-2 inhibitors toward indomethacin. Among all compounds tested ex vivo against TNF-α, after docking studies on this target, the best suppression was shown by compounds **155f** (70.20%) and **155g** (68.43%), which was higher than that of indomethacin, which suppresses TNF-α by 66.45%. Compounds **155d**, **155b**, and **155c** showed remarkable decreases in TNF-α concentration of 61.92–59.60%. Additionally, compounds **155f**, **155g**, and **155b** showed the best anti-inflammatory activity and did not show any gastric ulceration, which was probably due to their COX-2 selectivity.

Abdelazeem et al. [222], based on the recent studies that the COX-1 enzyme is not the only one that causes gastric ulcer [223,224,225,226], designed and synthesized novel 4,5-diphenylthiazoles **156** (Figure 60) to study their anti-inflammatory/analgesic and antiulcer activity. The design was based on isosteric replacement of an isoxazole ring in mofezolac, known COX-1 inhibitor, by thiazole. The evaluation of COX-1 inhibitory activity revealed that the most active were compounds **156a** and **156b** with an IC_50_ of 0.42 ± 1.03 and 0.32 ± 1.12 respectively, indicating that the presence of two methoxy groups improves the activity (**156b**). These compounds were found to be more active and selective toward COX-1 than COX-2 (Figure 59). The analgesic activity of these compounds was evaluated using the acetic acid-induced writhing test with diclofenac as a reference drug [226]. The results were in accordance with that of in vitro COX-1 inhibition, with compounds **156a** and **156b** bearing the free carboxylic group to be the most potent with the number of writhes between 10.67 and 14.40 compared to diclophenac (number of writhes 24).

The anti-inflammatory activity of synthesized compounds was tested using carrageenan-induced mouse paw edema assay with diclophenac as the reference drug [222,227]. In contrast to analgesic activity compounds, **156a** and **156b** showed moderate activity with edema inhibition of 53.99% and 50.30%, respectively. As far as anti-ulcer activity is concerned, the best activity was achieved for compounds **156a** and **156b** with ulcer indexes of 9.39 and 8.5, respectively. The molecular docking studies revealed similarity in docked poses of compound **156b** and mofezolac, explaining the significant activity and selectivity of compound **156b.**

**Figure 61:**
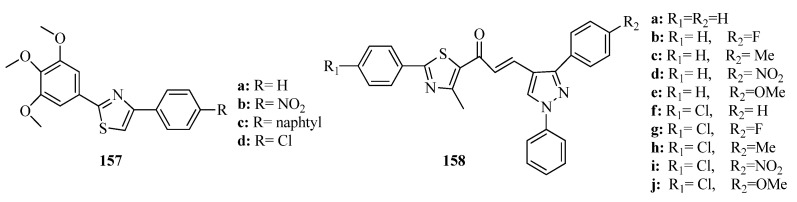
Structures of 4,5-disubstituted 2-(trimethoxyphenyl)thiazoles and thiazole-based chal cones.

Taking into account that it is approved by the US Food and Drug Administration (FDA), celecoxib still keeps some degree of COX-1 inhibition, being least specific among other coxibs showing a higher percentage of COX-1 inhibition than other coxibs [228]. Oniga et al. [229] aimed to develop only slightly COX-2 inhibitors and not COX-2 selective, mimicking the meloxicam pharmacological profile. In their previous studies [230,231,232], they designed and synthesized a series of thirteen 4,5-disubstituted 2-(trimethoxyphenyl)thiazoles **157a**–**m** (Figure 61) and evaluated their anti-inflammatory activity. Prompted by the obtained results, the authors determined their COX-1/COX-2 inhibitory activity as well as evaluated selectivity index.

The in vitro experiment revealed that the inhibitory activity of tested compounds varied. The best IC_50_ value of 26.88 μΜ was achieved for **157c** with a selectivity index (SI) of 9.24 near to that of meloxicam (SI 11.03) used as a reference, while **157a**, **157e**, **157f**, and **157d** also expressed good COX-1 inhibition. On the other hand, compound **157a** with an IC_50_ of 23.26 μΜ was the best COX-2 inhibitor was followed by compounds **157b**–**d** with an IC_50_ < 30 μΜ. The structure–activity relationships study indicated the importance of the substituents at position 4 of phenyl ring for selectivity for COX-2 without changing the affinity.

The molecular docking studies showed that compound **157b** fitted well to the binding site of COX-2 enzyme, occupying the similar region with meloxicam. Thus, the NO_2_ group has a hydrogen bonding interaction with Met522 and Trp387 in the active site.

Sirsat et al. [233] synthesized a series of thiazole-based chalcones and evaluated their anti-inflammatory activity by the denaturation of egg albumin using diclofenac as a reference compound. The majority of compounds showed anti-inflammatory activity. Compounds **158j**, **158e**, **158h**, and **158c** (Figure 61) exhibited a remarkable inhibition of protein denaturation from 71% to 86% compared to diclophenac (90.21%). The best activity was achieved for compound **158j** (86%). Compounds **158f**, **158g**, and **158b** displayed good anti-inflammatory activity, while the rest showed moderate inhibition.

Kerdawi et al. [234] reported the synthesis of a new series of benzimidazothiazole derivatives with the aim to evaluate their anti-inflammatory activity and mechanism of action.

**Figure 62:**
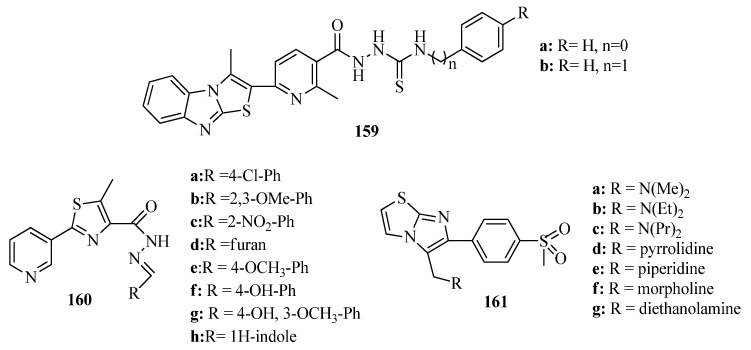
Structures of benzimidazothiazole, thiazole-based hydrazid and 4-(methylsulfonyl)phenyl)imidazo[2,1-b]thiazole derivatives.

The evaluation of anti-inflammatory activity was performed by carrageenan-induced mouse paw edema [235,236] with naproxen and celecoxib as reference drugs. The anti-inflammatory activity of compounds was in range of 49.0–79.5% Compounds **159a** and **159b** (Figure 62) showed the best inhibition of edema (79.5% and 75.9% respectively. In order to study the mechanism of anti-inflammatory activity, evaluation of COX-1/COX-2 inhibition was performed. The results revealed that compounds **159a** (IC_50_ 4.040 nΜ and 4.520 nM, respectively) and **159b** (IC_50_ 7.50 nΜ and 16.02 nM) were the most active COX-1/COX-2 inhibitors compared to celecoxib (IC_50_ 15.00 μΜ and 40.00 nM) and naproxen (IC_50_ 8.70 μΜ and 520.0 nΜ) with compound **159a** being more than 10 times more active than celecoxib and 115 times more potent than naproxen.

Kamat et al. [73] synthesized thiazole-based hydrazide derivatives and evaluated their anti-inflammatory activity by inhibition of the protein (bovine albumin) denaturation method using diclofenac sodium as a standard drug. The inhibition was determined using concentrations varying from 20 to 100 μg/mL. The highest inhibition was observed for concentration 100 μg/mL. All compounds showed inhibitory activity varying from 49.7 to 87.97%. Compound **160g** exhibited the best anti-inflammatory activity with 87.97% inhibition and IC_50_ 46.29 μg/mL, as compared to reference drug (94.6%, IC_50_ 35.03 μg/mL, Figure 61). It is obvious that the inhibitory activity of compounds is dependent on the nature and position of the substituent. Thus, the presence of 4-hydroxy = 3-methoxy substitution (**160g**, Figure 62) was found to be beneficial, while 3,4-dimethoxy-(**160b**) or 4-hydroxy (**160f**) were less potent with IC_50_ values of 76.12 and 62.74 μg/mL, respectively. Compounds with heterocyclic substituents (**160d**, **160h**) showed higher anti-inflammatory activity than compounds with aromatic substituents (**160a**, **160c**, **160e**, Figure 62). From the obtained results, it can be concluded that the presence of a hydroxyl substituent on the phenyl ring and/or heterocyclic moiety enhances the activity.

Zarghi et al. [237], prompted by their previous results [238], synthesized a new group of (4-(methylsulfonyl)phenyl)imidazo[2,1-b]thiazole derivatives possessing a methylsulfonyl at the para position of the C-6 phenyl ring and a different Mannich base on C-5 (**161a**–**g** Figure 62). For the evaluation of their COX-1 and COX-2 inhibitory activity, a chemiluminescent enzyme assay was used. The in vitro COX-1/COX-2 inhibition results indicated that all compounds were selective COX-2 inhibitors with IC_50_ in the range of 0.08–0.16 μΜ.

The most potent inhibitor among all compounds tested proved to be *N,N*-dimethyl-1-(6-(4-(methylsulfonyl)phenyl)imidazo[2,1-b]thiazol-5-yl)methanamine (**161a**) with IC_50_ 0.08 μΜ. It was observed that large alkylamino substituents such as diethylamino (**161b**) and dipropylamino (**161**c) were not favorable for the activity, decreasing it as well as selectivity. Among derivatives with cyclic amines (**161d**–**f**), which in general showed moderate activity, the more potent and selective appeared to be morpholino derivative (**161f**) with IC_50_ 0.10 μΜ and SI 551.

**Figure 63:**
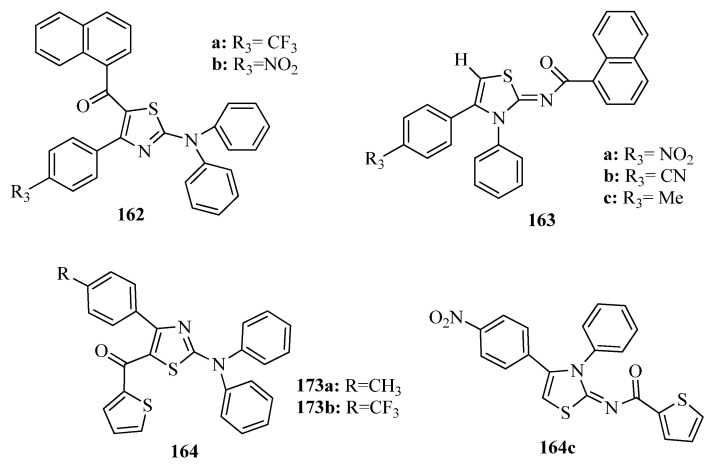
Structure of 2-amino-4-arylthiazol-5-ylnaphthalen-1-yl methanone derivatives, 2-(substituted)-4-(4-substituted phenyl)thiazol-5-yl)(thiophen-2-yl)methanone and N-(3-phenyl-4-(4-(substituted phenyl)thiazol- 2(3H)-ylidene)thiophene-2-carbox-amide.

Jakob et al. [239], taking into account that thiazoles are associated with various biological activities and enthused by their earlier studies [240], designed and synthesized 2-amino-4-arylthiazol-5-ylnaphthalen-1-yl methanone derivatives. All compounds were evaluated for their COX-1/COX-2 and LOX inhibitory activities. The investigation revealed that ten compounds showed good COX-2 inhibitory activity with IC_50_ in the range of 0.07–0.99 μΜ. Among them, the best activity was achieved for compound **162b** (Figure 63) with an IC_50_ of 0.07 μΜ and selectivity index (SI) of 115.4, which was higher than that of etoricoxib, 91.28, which is used as a reference drug. The next compound with good activity (IC_50_ 0.08 μΜ) was **163a** with SI 90. The good activity of compounds **163b** and **163a** (Figure 63) probably is due to the naphthoyl moiety, which provides bulkiness to the structure, since COX-2 has a larger active site. Compound **162a** was also among the best of compounds in terms of COX-2 inhibition but with low SI (24.71). The rest of the compounds exhibited moderate activity.

All compounds were also evaluated for soybean 5-LOX inhibitory activity. The best activity was shown by compounds **163b** and **163c** with IC_50_ values of 0.15 and 0.16 μΜ, respectively being equipotent to zileuton, which was used as the reference drug. Good activity was found also for compound **162b** (IC_50_ 0.29 μΜ). The rest of the compounds showed moderate LOX inhibition. Finally, compound **162b** was tested in vivo for anti-inflammatory activity by carrageenan-induced mouse paw edema [240,241], showing 63% inhibition in a dose of 20 mg/kg.

In their next work, Jakob et al. [242] reported the synthesis of 2-(substituted)-4-(4-substituted phenyl) thiazol-5-yl)(thiophen-2-yl)methanone and N-(3-phenyl-4-(4-(substituted phenyl)thiazol-2(3H)-ylidene)thiophene-2-carboxamide by multi-component one-pot green synthesis. All compounds were screened for COX-1/COX-2 and LOX inhibitory activity.

The study of inhibitory activity revealed that six compounds showed good inhibitory activity against both COX-2 and LOX enzymes with IC_50_ varied from 0.07 to 0.62 μM and 0.38–5.07 μM, respectively. The best activity toward COX-2 enzyme was achieved for compound **164a** (Figure 63) with IC_50_ = 0.09 ± 0.002 μM, while IC_50_ for COX-1 was 5.55 ± 0.77 μM. The selectivity index of this compound (SI 61.66) was higher than SI of the rest of compounds and comparable to etoricoxib (IC_50_ = 0.07 ± 0.007 μM, SI = 91.28), a known selective COX-2 inhibitor, which was used as the reference drug.

The investigation of LOX inhibitory activity revealed that compounds **164a** (IC_50_ = 0.38 ± 0.01 μM) and **164b** (IC_50_ = 0.39 ± 0.01 μM) showed significant inhibition compared with the reference drug zileuton (IC_50_ = 0.14 ± 0.01 μM) followed by thiazolidene derivative **164c** with IC_50_ = 0.58 ± 0.04 μM. However, the activity of thiazole derivatives was superior to thiazolidene ones**.** Thus, it can be concluded that compound **164a** is a dual COX-2/LOX inhibitor that was proved by docking to COX-2 and the LOX active site. This compound had the best binding pose in the COX-2 active site with binding energy −7.54 Kcal/mol forming a hydrogen bond through the oxygen of the carbonyl group with His351 amino acid residue. Compound **164a** formed two hydrogen bonds in the active site of LOX, one between the sulfur atom of the thiazole ring and Trp147 amino acid residue and another with the sulfur atom of the thiophene ring and Gln417, with binding energy −6.99 Kcal/mol better than zileuton (−6.43 Kcal/mol).

### 3.8. Antioxidant

In recent years, antioxidants attracted a lot of attention due to their potential as prophylactic and even therapeutic agents in many diseases. Reactive oxygen species (ROS) are constantly formed as a result of normal organ functions or under excessive oxidative stress conditions. High levels of free radicals could be responsible for the damage in various biological macromolecules, such as proteins, lipids, enzymes, and DNA in cells and tissues, resulting in mutations that can lead to cancer. Moreover, high levels of free radicals give rise to a number of inflammatory [243], neurodegenerative and metabolic disorders [244,245,246], cardiovascular and autoimmune diseases, and cellular aging [247,248]. Reducing oxidative damage may be an important approach to the primary prevention and treatment of these diseases. Over the past few years, the development of new synthetic compounds bearing the thiazole moiety as novel antioxidant agents has been a great success.

**Figure 64:**
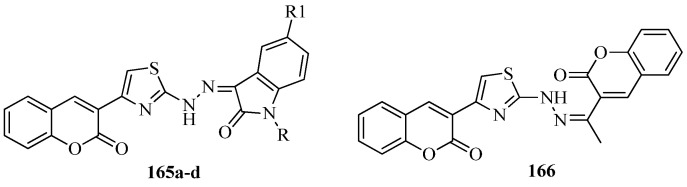
Structures of thiazolyl coumarin derivatives (**166a**, R = H, R’ = H; **166b**, R = CH_2_–C_6_H_5_, R’ = H; **166c**, R = H, R’ = F; **166d**, R = H, R’ = I).

In 2015, Thota et al. [249], based on the literature about the pharmacological action of fused heterocycles, decided to synthesize a new series of compounds containing both indole and thiazole-substituted coumarin moieties and reported their in vitro cytotoxic and antioxidant activities. The antioxidant activity of the synthesized compound (Figure 63) was evaluated in vitro by the DPPH scavenging method, and the results were compared with that of ascorbic acid. All the tested compounds exhibited good antioxidant activity compared to the standard drug ascorbic acid (IC_50_ 12.27 ± 0.86 μg/mL, inhibition concentration), while compounds **165c**, **165d**, and **166** (Figure 64) were found to be the most potent (IC_50_ 11.04 ± 0.18, 11.28 ± 0.06, and 12.16 ± 0.28 μg/mL, inhibition concentration, respectively).

**Figure 65:**
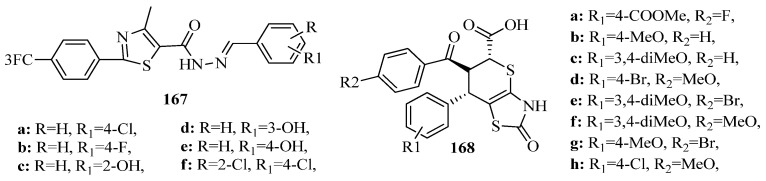
Structures of new acyl-hydrazones bearing thiazole scaffold (**167**) and new thiopyrano[2,3-*d*]thiazoles (**168**).

Nastasă et al. [250] synthesized a new series of hydrazones bearing thiazole scaffolds and evaluated their antioxidant activity using the DPPH method. All the tested compounds demonstrated low to moderate antioxidant activity, which was lower than the reference drug ascorbic acid. Compounds **167c**–**e** (Figure 65) bearing a hydroxyl group at the ortho, meta, and para position of the phenyl ring, respectively, exhibited the best DPPH activity among the others tested. This result confirms the theory that a hydroxyl group enhances the antioxidant activity. The activity of monohydroxy compounds mainly depends on the position of the hydroxyl group. Para-substitution has more stabilizing potential than meta-substitution. Actually, compound **167e** with the hydroxyl group in position 4 of the phenyl ring exhibited better activity than ortho- and meta derivatives. The lower activity of compound **167c** may be due to the intramolecular hydrogen bonding. New compounds containing fluorine (**167b**), in position para, or chlorine (**167f**) showed better activity than the others. Moreover, the addition of another chlorine atom on the structure of **167a**, in compound **167f**, increased the antioxidant activity.

Based on their ongoing efforts, Lozynskyi et al. [251] presented the synthesis and antioxidant activity of a new series of substituted thiopyrano [2,3-*d*]thiazoles. The results of the DPPH assay revealed that most of the compounds have moderate to good antioxidant activity compared to L-ascorbic acid, which was used as a reference drug. Compounds **168c**, **168e**–**h**, **168k**, **168l**, and **168p** (Figure 65) exhibited inhibition at a range of 43–77%. Lozynskyi et al. proposed that the presence of substituents (especially methoxy group) in phenyl and benzoyl fragments (position 6 and 7 of thiopyrano [2,3-d]thiazole core) is favorable for the antioxidant potency. The antioxidant potency of the compounds is probably linked to their electron or hydrogen radical releasing ability to DPPH, and in that way, they become stable diamagnetic molecules. It can also be explained by EHOMO and ELUMO; thus, compounds that possess higher EHOMO and ELUMO can be more potent agents for stabilizing DPPH radicals.

**Figure 66:**
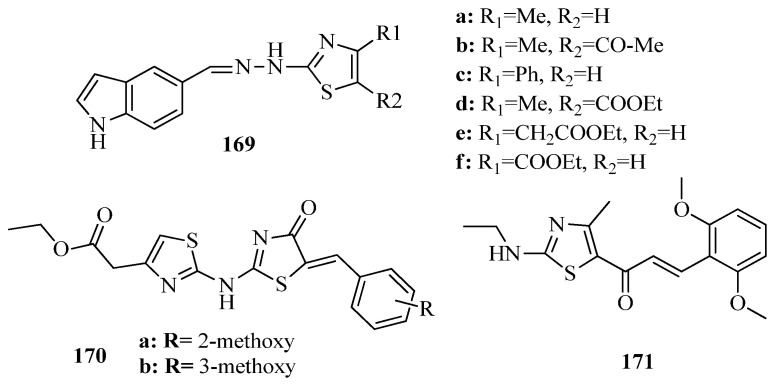
Structures of new 2-(2-((1H-indol-5yl)methylene)-hydrazinyl)–thiazoles (**169a**–**f_a; b; c; d; e; f; a; b; c; d; e; f; a; b; c; d; e;_**), structures of compounds **170a**, **170b**, and **171**.

In 2017, Grozav et al. [252], in continuation of their work and considering previous literature, designed and synthesized a novel series of 2-(2-((1*H*-indol-5yl)methylene)-hydrazinyl)-thiazole derivatives (Figure 66) and evaluated their antioxidant activity using three methods: DPPH, FRAP, and Electron Paramagnetic Resonance. The scavenging potential of the synthesized compounds was estimated by the DPPH method and decreases in the following range: **169a** > **169c** > **169b** > **169e** > **169d** > **1169f.** Compounds **169a** (Figure 66, IC_50_ of 5.377 μg/mL) and **178c** (IC_50_ of 9.131 μg/mL) displayed higher antioxidant activity than Trolox standard (IC_50_ = 9.74 μg/mL) but worse than ascorbic acid (IC_50_ of 2.46 μg/mL). The antioxidant activity of the compounds **169a**–**f** was evaluated by EPR spectroscopy, too. In the mixture with the tested compounds, the integral density of DPPH radicals is reduced compared to free DPPH solution (I = 252) and displays the oxido-reduction rate. The order of reactivity is identical to the one found by the DPPH spectrophotometric method. The reducing capacity of compounds **169a**–**f** has been measured as ferric-reducing antioxidant power (FRAP). The reduction of Fe^3+^ ion from tripyridyltriazine Fe (TPTZ)^3+^ complex to Fe^2+^ ion in the blue-colored Fe (TPTZ)^2+^ complex is affected by the electron-donating capacity of the tested compounds **169a**–**f** and was quantified by the spectrophotometric method. The reducing ability of the compounds can be presented as follows: **169a** > **169c** > **169e** > **169d** > **169f** > **169b.** Taking into account the results of all the three methods for the antioxidant activity, compound **169a** is the most active of the series.

As a continuation of their previous work, Djukic et al. [253] evaluated the antioxidant activity of three newly synthesized thiazolidinone derivatives of 1,3-thiazole (Figure 66) using three in vitro tests: DPPH, FRAP, and TBARS assay. In the DPPH assay, compounds exhibited low antioxidants activity. In the FRAP assay, compounds **170a** and **171** (Figure 66) were found to have moderate antioxidant activity, which was higher when compared to α-LA, while compound **170b** exhibited good antioxidant activity but still lower than that of vitamins C and E. As far as the TBARS assay is concerned, compounds **170a** and **171** (62.11% and 60.93% respectively) exhibited very good antioxidant activity, which was almost identical with vitamin C (62.32%), while compound **170b** demonstrated the lowest activity (23.51%).

**Figure 67:**
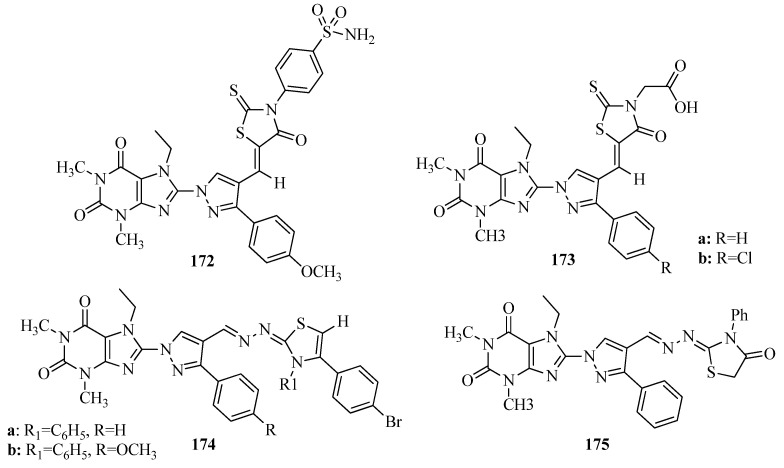
Structures of purine–pyrazole hybrids bearing thiazole moiety.

In continuation of their ongoing efforts, Afifi et al. [254] in 2019 reported the synthesis and biological evaluation of purine–pyrazole hybrids incorporating thiazole, thiazolidinone, or rhodanine moiety. The antioxidant screening was carried out with two methods: determination of the free radical scavenging activity by the 1,1-diphenyl-2-picrylhydrazyl (DPPH) antioxidant screening assay method and determination of nitric oxide radical scavenging activity. Among the tested compounds, compound **175** was found to be the best scavenger. In addition, compounds **173a**, **173b**, and **174a**–**b** (Figure 67) exhibited good DPPH free radical scavenging potential with IC_50_ ranging from 4.89 to 14.43 μg/mL, higher than that of the standard ascorbic acid with IC_50_ 15.34 μg/mL. In addition, compounds **172**, **173a**–**b**, and **174a** exhibited potent nitric oxide scavenging ability.

**Figure 68:**
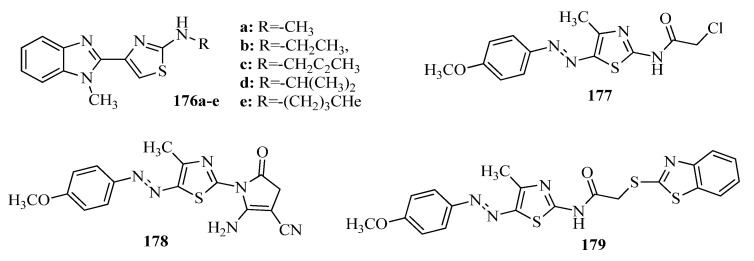
Structures of. 2-alkylamino-4-(1-methylbenzimidazol-2-yl)thiazoles and 4-methyl5-(phenylarylazo)thiazol-2-yl derivatives.

In 2019, Chithra et al. [255] successfully synthesized a series of new 2-alkylamino-4-(1-methylbenzimidazol-2-yl) thiazoles **176a**–**e** (Figure 68) and evaluated their antioxidant potential by DPPH assay. The results of the DPPH method revealed that the compounds have very good scavenging activity. The scavenging potential in terms of hydrogen-donating ability decreases in the following range: **176b** > **d** > **e** > **a** > **c.** The antioxidant potential of all the tested compounds is better than the standard BHA. Chithra et al. studied also the HOMO and LUMO energy values. The antioxidant potential of all the tested compounds is better than the standard BHA.

Hossan et al. [256] synthesized a series of novel 4-methyl-5-(phenylarylazo)thiazol-2-yl derivatives **177**–**179** (Figure 68), which were screened in vitro to evaluate their antioxidant activity through the DPPH radical spectrophotometric technique using ascorbic acid as a positive control. Furthermore, molecular docking was carried out to estimate their antioxidant efficacy (PDB code: 3MNG) as antioxidant enzyme receptor. The results revealed that the methoxy derivatives in each series exhibited good antioxidant activity in comparison to the rest of the analogues, and this may be attributed to the action of the methoxy group as an electron-releasing group that increases the electronic cloud along the molecule.

### 3.9. Thiazole Derivatives as Carbonic Anhydrase Inhibitors

Carbonic anhydrises (CAs, EC 4.2.1.1) are important enzymes [257] that catalyze the formation of bicarbonate from carbon dioxide in two steps [258,259,260]. Since the CA activity of some isoforms is unregulated, leading to different diseases, the studies of inhibitors or activators of the CA activity is very important. Among the inhibitors of CA isoforms are drugs for the treatment of glaucoma and epilepsy, which have been used for decades [261]. Inhibitors of some other isoforms were studied against tumors, obesity, or as anti-infectives [68]. Many CA subtypes constitute interesting targets for the design of pharmacological agents.

Many different compounds such as nitro [262], uracil derivatives [263], bromophenoles [264], thiureas [265], coumarins [266], and many others have shown CA inhibitory activity. However, the most important class is sulfonamides [267,268,269].

**Figure 69:**
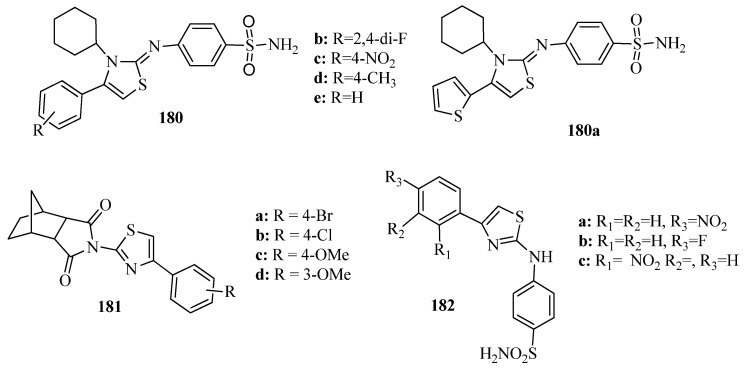
Structures of 4-[(3-cyclohexyl-4-aryl-2,3-dihydro-1,3-thiazol-2-ylidene) amino]benzene-1-sulfonamides, 2-(4-(aryl)thiazole-2-yl)-3a,4,7,7a-tetrahydro-1*H*-4,7-methanoisoindole-1,3(2H)-dione, and sulfonamide-bearing thiazole derivatives.

Keeping all these in mind and based on the results of Sechi [270], Maccioni et al. [271] designed and synthesized a series of 4-[(3-cyclohexyl-4-aryl-2,3-dihydro-1,3-thiazol-2-ylidene) amino]benzene-1-sulfonamides and evaluated their inhibitory activity toward hCA I, II, IX, and XII. Among the tested compounds, only **180a** (Figure 69) showed good inhibitory activity against hCA II isoform with an inhibition constant (Ki) of 1.8 nM. On the other hand, all these compounds inhibited hCA II isoform in the nanomolar range with Ki values from 0.07 to 91.5 nM, except for MAC8001a. Again, the best activity was observed for compound **180a** (Ki = 0.07 nM) compared to acetazolamide (AAZ), which was used as a reference drug (Ki = 12 nM). Compounds **180e** and **180c** exhibited good activity against CA IX isoform (KI = 26.9, 29.9, and 29.5 nM) better than AAZ (Ki = 25 nM), but none of them were selective toward the enzyme. All tested compounds showed inhibitory activity in the nanomolar range against CA XII isoform with KI values ranging from 0.46 to 29.5 nM. Three compounds, **180b**, **180d,** and **180a,** were found to be more potent (Ki = 5.5, 5.3, and 0.46 nM) than AAZ (Ki = 5.7 nM). The binding mode in a way proves the importance of substitution at position e and 4 of the thiazolidinone moiety because it allows better accommodation in the binding pocket. The nature of the substituent at position 4 of the dihydrothiazole ring was found to play an important role for determining the activity and selectivity of these compounds.

Kocyigit et al. [272] synthesized a number of novel 2-(4-(aryl)thiazole-2-yl)- 3a,4,7,7a-tetrahydro-1H-4,7-methanoisoindole-1,3(2H)-dione derivatives (Figure 69) and evaluated their inhibitory characteristics against the human CA isoenzymes I and II (hCA I and hCA II). All compounds showed inhibitory activity against hCA I isoform with an IC_50_ range of 26.0–31.5 nM and Ki ranging from 27.07 to 37.80 nM. The best activity was expressed by compound **181a** (Figure 69) with IC_50_ and Ki values of 26.65 nM and 27.0 ± 4.11 nm, respectively (Figure 66). Compound **181c** also showed good activity with an IC_50_ of 26.0 nM, but its constant of inhibition was higher (Ki 32 ± 12.21 nM) than that of compound **181a**. Nevertheless, both compounds were more potent than the reference drug acetazolamine (AZA) with IC_50_ and Ki values of 40.46 and 34.5 ± 0.02 nM, respectively. Synthesized compounds were also found to be inhibitors of hCA II isoform with IC_50_ in the range of 21.0–24.75 nM and Ki of 11.80–25.81 nM. It is obvious that the inhibitory activity of compounds against the hCA II isoform is better than that against hCA I. Compound **181b** demonstrated the highest activity with IC_50_ at 20.38 nM and Ki at 11.8 ± 1.78 nM, which was better than that of AZA (IC_50_ = 24.16 nM and Ki = 28.93 ± 0.05 nM). The second-best compound according to the constant of inhibition was found to be **181d** (Ki = 18.96 ± 4.26). From the obtained results, it seems that these compounds can be lead compounds for the development of more active CA inhibitors.

Kılıcaslan et al. [273] reported the synthesis of sulfonamide-bearing thiazole (**182a**–**l**, Figure 69) in order to evaluate their inhibitory activity against hCA I and hCA II isoforms. All compounds possessed inhibitory activity against both isoforms with IC_50_ in range of 0.35–1.34 μΜ against hCA I and 0.35–1.56 μΜ against hCA II. 4-Nitro derivative **182a** exhibited the best activity among all compounds tested against both isoforms (IC_50_ of 0.35 μΜ), followed by **182b** (4-F-derivative) with an IC_50_ of 0.37 μΜ, indicating the positive role of these groups for CA inhibitory activity. While 4-nitro derivative was the most potent, 2-nitro (**182c**) was the less potent, which was probably due to sterical impairment for the binding of the compounds to the Zn(II) ion in the enzyme active site. It should be mentioned that the compounds are non-competitive inhibitors with Ki values for hCA I (Ki = 0.33–1.21 μΜ) being close to the clinically used sulfonamide AZA (acetazolamide, Ki = 0.250 μM), whereas Ki = 0.11–1.99 μM for hCA II were higher than AZA (Ki = 0.012 μM).

**Figure 70:**
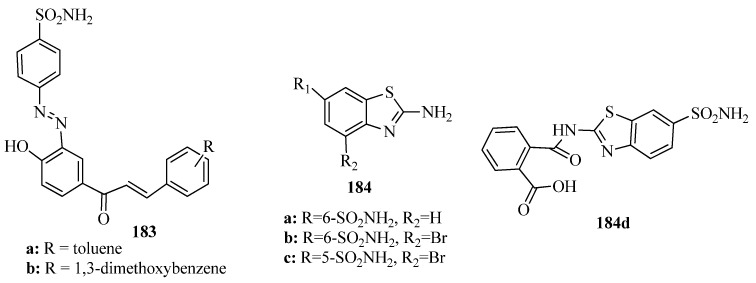
Structures of sulfonamide derivatives.

Arslan et al. [274] synthesized a new series of sulfonamide derivatives incorporating substituted chalcone core in the frame of one molecule and studied their inhibitory activity against CA cytosolic isoforms I and II. The study revealed that all compounds showed inhibitory activity against hCA I isoform with Ki in range of 9.88–24.4 nM. Compound **183b** (Figure 70) was found to be the most promising with a Ki value of 9.88nM, compared to AZA used as the reference drug (Ki of 250 nM). As far as the hCA II isoform is concerned, the compounds exhibited activity with Ki values in the range of 18.25–55.43 nm. The highest activity among them was achieved for compound **183a** (Ki of 18.25 nM) compared to AZA (Ki of 12 nM). Although all compounds demonstrated quite a good inhibitory profile against both CA isoforms, their selectivity was low.

Abdoli et al. [275] reported the synthesis of a series of benzo[d]thiazole-5- and 6-sulfonamides **184a**–**d** (Figure 70) and evaluated their CA inhibitory activity against hCAI, II, VII, and IX isoforms using a stopped flow CO_2_ hydrase assay [276]. All compounds inhibited all four hCA isoforms but with a different range of inhibition constants. Thus, the range of Ki for hCA I was 84.1–2327 nM. The most active compounds were found to be compound **184a** and **184d** with Ki values of 84.1 and 97.1 nM, while the Ki for hCA II was in the range of 7.8–369 nM. In this case, the best activity was shown by compounds **184b**, **184c,** and **184d** with Ki values of 8.7, 7.8, and 13.5 higher or equal with that of AZA (12.1 nM) (Figure 67). As far as the hCAVII isoform is concerned, the Ki value range was much lower, 0.8–81.5 nM, with the highest activity achieved for compound **184c** (Ki = 0.8 nM), while for the hCA IX isoform, the inhibition constant ranged from 3.6 to 1000 nM. The best activity against this isoform was exhibited by compounds **184a** and **184d** with Ki values of 3.7 and 10 nM respectively better than that of both reference drugs AZA (Ki = 25.8 nM) and EZA (Ki = 34.2 nM). Thus, the most active compounds against four isoforms were compounds **184a**–**d**, which can be a starting point for the further development of more effective agents.

**Figure 71:**
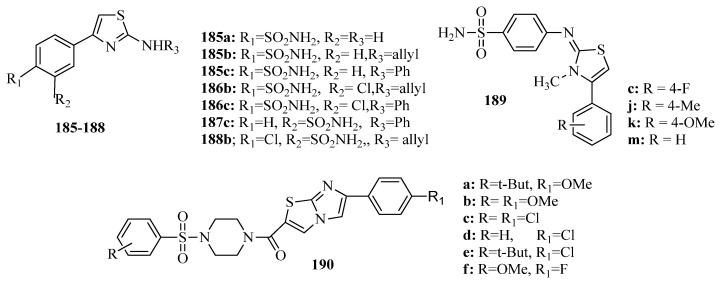
Structures of para-or meta chloro-substituted thiazolylbenzenesulfonamides, 4-[(3-methyl-4-aryl-2,3-dihydro-1,3-thiazol-2-ylidene)amino]benzene-1-sulphonamides and imidazo[2,1-b]thiazole-sulfonyl piperazines (**190**).

Capkauskaite et al. [277] designed and synthesized four series of para- or meta chloro-substituted thiazolylbenzenesulfonamides **185**–**188a**–**c** (Figure 71) and evaluated their inhibitory activity against all catalytically active recombinant human carbonic anhydrase (CA) isoforms using the fluorescence-based thermal shift assay (FTSA). Compounds **185b** and **186b** were the most potent inhibitors of CA I isoform with dissociation constants (Kd) of 25 and 16.7 nM and intrinsing dissociation constants (Kd*int*) of 0.03 and 0.06 nM, respectively. Kd and Kd*int* of AZA were 1800 and 810 nM, respectively. Additionally, compound **185b** was the most potent against CA II isoform (Kd = 71.4 nM) but less active than AZA (Kd = 46.98 nM). None of the compounds tested showed good activity against CA III, CA VA, CA VB, CA VI, and CA XII isoforms, while compounds **188b** (Kd = 200 nM) and **188c** (Kd = 417 nM) were among the most active ones against CA IV isoform compared to AZA (Kd 87 nM). Compound **186c** showed the highest activity (Kd = 33.3 nM) against CAV isoform compared with AZA (Kd = 840 nM), while compound **186b** was the most active among all tested compounds against CAVII isoform (Kd = 154 nm) but less active than AZA (Kd = 13 nM). The highest activity against CA IX isoform was shown by compounds **185a** and **185c** with Kd of 66.7 nM, which does not exceed the activity of AZA (Kd = 13 nM). Compound **186c** exhibited excellent activity against CA X III isoform (Kd = 33.3 nM), which was almost 4-fold higher than that of AZA (Kd = 120 nM), whereas compound **185b** was the best (Kd = 40 nM) against CA XIV isoform, which was better than AZA (Kd = 63 nM). The general observation of this study is that the para-substituted benzenesulfonamides (compounds **185**–**186**(**a**–**c**)) exhibited higher binding affinity than their meta-substituted benzenesulfonamide analogs (compounds **187**–**188**(**a**–**c**)**.**

Based on their previous observations [278,279,280,281], Distinto et al. [282] designed a library of 4-[(3-methyl-4-aryl-2,3-dihydro-1,3-thiazol-2-ylidene)amino] benzene-1-sulphonamides and synthesized in order to evaluate the effect of substituents in positions 3 and 4 of the dihydrothiazole ring on the inhibitory potency and selectivity toward human carbonic anhydrase isoforms I, II, IX, and XII. The study revealed that in accordance with their previous observations for compounds with similar structure, none of the synthesized compounds was active against hCA I isoform. Instead, some compounds showed very good activity against hCA II, IX, and XII isoforms. Thus, compounds **189a**–**d** (Figure 71) showed good activity against hCA II isoform with Ki values of 5.3, 4.5, 3.8, and 5.3 nM, respectively being more potent than reference drug AAZ (Ki = 12 nM). It seems that the introduction of halogen with a small atomic radius at the 4 position of the phenyl ring at the 4 position of thiazole moiety is beneficial. The introduction of halogen with a larger atomic radius led to a decrease of activity. Consequently, the Ki are 4-F (**189a**) 5.3 nM < 4-Cl 13.1 nM < 4-Br 45.8 nM. It is interesting to mention that the unsubstituted derivative **189d** exhibited the same activity with 4-F derivative **189a.** In case of hCA IX isoform, the highest activity was achieved for compounds **189b** (Ki = 23.3 nM) and **189c** (Ki =25.4 nM), which demonstrated activity lower or equal with reference drug (Ki = 25 nM). In this instance, the role of halogen was the opposite. The larger the atomic radius, the larger the activity (4Br > 4-Cl > 4-F). Compounds **189b** and **189c** were also found to exhibit very good activity with KI values of 3.1 and 4.6 nM against hCA XII isoform being more potent than AAZ (Ki = 5.7 nM). In case of hCA XII, the presence of dihalogens was beneficial, following the order 2,4-di-F > 2.4-di-Cl. Finally, the introduction of 4-Me and 4-OMe substituents at the position 4 of the main core seems to be beneficial for inhibitory activity against hCA II, IX, and XII isoforms.

Alvala et al. [283] synthesized new imidazo [2,1-b]thiazole-sulfonyl piperazines (**190a**–**f**, Figure 71) and screened for their CA inhibitory activity against cytosolic (hCA I, hCA II) and the tumor-associated (hCA IX, hCAX II) isoforms using a stopped flow CO_2_ hydrase assay [269] and AAZ as a reference drug. None of compounds inhibited the hCA I cytosolic and the tumor-associated hCA IX, hCA XII isoform. However, some compounds **190a**–**f** exhibited inhibitory activity against hCA II isoform with Ki values in the range of 57.7–79.9 nM. The best activity was achieved for compound **190a** with Ki of 57.7 nm followed by **190c** (Ki = 57.8 nM) and **190e** with Ki of 62.1 nM. Nevertheless, none of these compounds exerted the activity of AAZ Ki = 0.012 nM.

**Figure 72:**
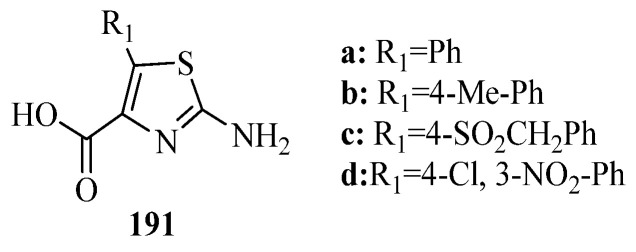
Structure of 4,5-trisubstituted thiazole derivatives.

Jaidi et al. [284] presented the synthesis of 17 compounds with 2,4,5-trisubstituted thiazole scaffold and evaluation of their inhibitory activity against cytosolic CA III isoform. For the evaluation, size exclusion HDM chromatography [285,286,287] was used due to the low catalytic activity of CA in CO_2_ hydration. The study revealed that only compounds **191a**–**d** (Figure 72) inhibited CA III isoform with Ki in range of 0.5–86.6 μΜ. The unsubstituted at the para position of the phenyl ring compound **191a** exhibited the best activity with Ki of 0.5 μΜ better than that of reference drug, vanillic acid (Ki = 6.8 μΜ). Substitution at the para position of the phenyl ring led to compounds **191b**–**d** with decreased activity (Ki = 18.9, 86.6, and 81.2 μΜ).

### 3.10. Anticonvulsant

**Figure 73:**
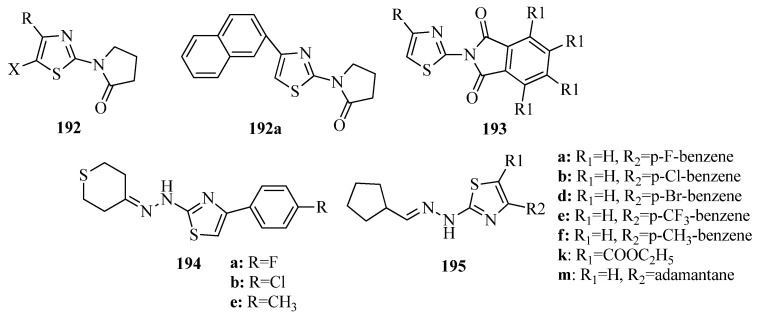
Structures of 2,4,5-trisubstituted thiazoles (**192**), 2-(thiazol-2-yl)isoindoline-1,3-diones(**193**) and 2-(cyclopentylmethylene)hydrazinyl-1,3-thiazoles.

In 2015, Ghabbour et al. [288] synthesized two new series of compounds, bearing the thiazole ring, and evaluated their anticonvulsant activity. They synthesized 13 new 1-(thiazol-2-yl)pyrrolidin-2-ones of general structure **192** and 14 new 2-(thiazol-2-yl) isoindoline-1,3-diones of general structure **193** (Figure 73). The activity was established in three seizure models: PTZ, picrotoxin and MES. The most active compound was found to be 1-(4-(naphthalen-2-yl) thiazol-2-yl) pyrrolidin-2-one (**192a**, Figure 73) showing a PTZ effective dose (ED_50_) value of 18.4 mg/kg in mice. A computational study was also carried out, including prediction of pharmacokinetic properties and docking studies.

Łączkowski et al. in 2016 [289] developed a small library of tetrahydro-*2H*-thiopyran-4-yl based thiazoles and selenazoles and tested them for their antimicrobial and anticonvulsant activity. Based on their previous results, which have shown that compounds containing -F, -Cl, and -CH_3_ substituents demonstrated significant anticonvulsant activity in pentylenetetrazole model, Łączkowski et al. decided to choose compounds **194a**, **194b,** and **194e** (Figure 73) for the anticonvulsant activity tests. The results of anticonvulsant screening revealed that compounds **194a**,**b** showed a statistically significant anticonvulsant activity in the pentylenetetrazole model, whereas compound **194a** showed protection in the 6-*Hz* psychomotor seizure model. It is worth noting that none of the compounds impaired animals’ motor skills in the rotarod test. Therefore, the active compounds can be considered as interesting new leading structures in the search of antiepileptic drugs.

Continuing their previous investigation, Łączkowski et al. [290] decided to incorporate cyclopentylmethylidene into the thiazol-2-yl-hydrazine pharmacophore containing various electron-withdrawing and electron-donating substituents, which can modulate electronic effects and may have an influence on the hydrophobic and hydrophilic properties of the synthesized compounds. Therefore, they synthesized 13 novel thiazole derivatives and evaluated the in vivo anticonvulsant properties, using the PTZ seizure, the maximal electroshock seizure tests, the pilocarpine-induced seizures, and the rotarod test. The in vivo pharmacological studies of the compounds have shown the significance of the substituent in the para position of the benzene ring (compounds **195a**–**l**, Figure 73) for their anticonvulsant activity. Seven of the synthesized compounds (**195a**, **195b**, **195d**, **195e**, **195f**, **195k** and **195m** (Figure 73) containing F-, Cl-, Br-, CF_3_-, CH_3_-, and adamantyl substituents exhibited the best anticonvulsant activity in the PTZ model. All the synthesized compounds demonstrated no effect on electrically induced seizures and no protection was shown on pilocarpine-induced seizures. It is also important to note that none of the synthesized derivatives impaired animals’ motor skills in the rotarod test. The compounds **195a** and **195b** delayed the onset of clonic seizures in a statistically significant manner and reduced the number of seizure episodes in the PTZ test, so they can be regarded as interesting lead structures for further investigation.

**Figure 74:**
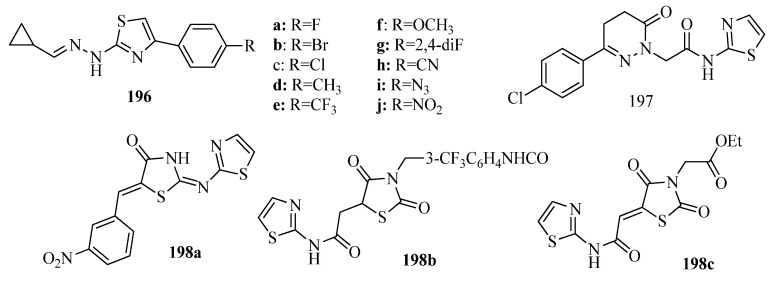
Structures of hydrazinylthiazole, hybrid pyridazinone–thiazole derivatives.

In 2018, Łączkowski et al. [291] based on their previous efforts and taking into account that in the past few years more drugs with a cyclopropyl moiety have accessed the clinical trials’ phase, designed and synthesized 10 new hydrazinylthiazole derivatives containing cyclopropyl group (**196a**–**j**, Figure 74). The synthesized compounds were tested for their antifungal, anticonvulsant and anti-Toxoplasma gondii activity. The anticonvulsant activity of the novel thiazole derivatives was evaluated in vivo in mouse models of electrically and chemically induced seizures. The four most active compounds were also tested for their influence on the animals’ motor coordination. At the MES test, compounds with chloro- and trifluoromethyl groups exhibited good anticonvulsant activity, but at the PTZ test, compounds with the best anticonvulsant activity were those containing methoxy- and azido group. Eventually, in the rotarod test, none of the tested compounds worsened animals’ motor skills.

Mishchenko et al. in 2020 [292] taking into account the structural demands for new anticonvulsants, synthesized new compounds with similar structure to the known anticonvulsant drug ralitoline, containing two thiazole moieties. The team tested the anticonvulsant activity of the compounds in two different assays: pentylenetetrazole-induced seizures (PTZ) and maximal electroshock seizure tests. Between the synthesized compounds, compounds, **197a** (5Z-(3-nitrobenzylidene)-2-(thiazol-2-ylimino)-thiazolidin-4-one), **197b** (2-[2,4- dioxo-5- (thiazol-2-ylcarbamoylmethyl)-thiazolidin-3-yl]-N-(2-trifluoromethylphenyl) acetamide), and **197c** ((2,4-dioxo-5-(thiazol-2-ylcarbamoylmethylene)-thiazolidin-3-yl)acetic acid ethyl ester) (Figure 74) exhibited very good anticonvulsant potential in both assays. It is important to note that these compounds were found to have low acute toxicity.

As a continuation of their previous efforts and based on the fact that compounds containing pyridazinone moiety have been known to be potent anticonvulsant agents, Siddiqui et al. [293] designed and synthesized a series of hybrid pyridazinone–thiazole compounds connected through amide linkage. Among the synthesized compounds, compound **198** (Figure 74) exhibited good anticonvulsant activity with median effective dose of 24.38 mg/kg at the MES test and 88.23 mg/kg at the PTZ test. The GABA estimation assay resulted in a remarkable increase in the GABA level in comparison with the control. The GABA modulatory effect of **198** was validated by the molecular docking study at the active site of GABA receptor.

## 4. Conclusions

During the period from 2015, a lot of thiazole derivatives were synthesized and evaluated for different kinds of biological activities such as antimicrobial, antiinflammatory, anticancer, antidiabetic, anticonvulsant, antitubercular, as well as carbonic anhydrase inhibitors and neglected diseases. Many of studied thiazole derivatives appeared to be more potent than reference drugs, being good candidates for further modification and development new active and safe derivatives.

Thus, for example, among thiazole derivative with antimicrobial activity 2,5-disubstituted thiazole derivatives demonstrated very good activity against MRSA, VISA and VRSA strains with compound T(Z)-2-(1-(2-(4-(hex-1-yn-1-yl)phenyl)-4-methylthiazol-5-yl)ethylidene)hydrazinecarboximidamid (**7**) exhibiting MIC in range of 0.7–2.8 μg/mL comparable with vancomycin (MIC 0.7–190.2 μg/mL.

Hydrazide and *N,N*-dimethylguanidine-containing derivatives 4-(2-(4-butylphenyl)-4-methylthiazol-5-yl)pyrimidin-2-amine compound with 2-(4-butylphenyl)-5-(2-hydrazinylpyrimidin-4-yl)-4-methylthiazole (18) (MIC 0.4 μg/mL) and its analogue **19** (MIC 0.78 μg/mL) were found to be more potent than vancomycin.

The thiazolyl-hydrazothiazole (**37b**) was superior to ampicillin (MIC 0.03 µg mL^−1^) against *S. pneumoniae*. On the other hand, functionalized 2,3-dihydrothiazoles tagged with sulfisoxazole moiety (**15a**–**e**) demonstrated very good, better than amphotericin B (MIC 23.7 μg/mL activity against *A.fumigatus, S. racemosum*, and *G. candidum* with MIC values in range of 11.5–26.9 μg/mL. Chlorophenyl thiazolocoumarinyl hydrazides were more potent than fluconazole (MIC 8 μg/mL) against *A. flavus*) and *A. fumigatus with MIC* being 0.4 μg/mL

2-Hydroxy-5-(2-(4-(2-(phenylamino) thiazol-4-yl)phenyl)thiazol-4-yl) benzamide (**27e**) displayed better activity (MIC at 7.81 μg/mL) compared to fluconazole (MIC 15.62 μg/mL) against *Candida albicans ATCC 10,231 and Candida krusei ATCC 6258.*

(E)-2-(2-(Cyclohex-3-en-1-ylmethylene) hydrazinyl)thiazole derivatives showed very good activity with MIC 0.015–3.91 μg/mL against C.species being equipotent with nystatin.

As far as antitubercular activity is concerned, thiazole derivatives in general showed moderate activity. No one derivative exceeded the activity of reference drugs.

Thiazole derivatives displayed different activity against different cancer cell lines. Thus, 2-(2-amino-4,5,6,7-tetrahydrobenzo[b]thiophen-3-yl)-7-imino-6,7-dihydro-5*H*-pyrano[2,3-d]thiazol-5-amine showed excellent activity, which was 35 times higher than the reference drug against HA22T, while 2-(2-(2-amino-4,5,6,7-tetrahydrobenzo[b]thiophen-3-yl)-4-oxothiazol-5(4*H*)-ylidene)-2-phenylhydrazin-2-ium-1-ide (**67a**) demonstrated the best activity against the NUGC cell line with an IC_50_ value of 23 ± 80 nmol/L higher than the reference drug CHS-828 (IC_50_ of 25 ± 10 nmol/L). Arylazothiazole derivative **72c** (IC_50_ of 4.9 ± 0.5 μM) against hepatocellular (HepG2) carcinoma cell was more potent than cisplatin (IC_50_ = 6.9 ± 0.7 μM). (Z)-4-(2,4-dichlorophenyl)-2-((E)-(4-(trifluoromethyl)benzylidene)hydrazono)-2,3-dihydrothiazole (**74d**) displayed the best activity against four tumor cell lines (NCI-H292, HEp-2, HT-29, K5620).

Among antiviral thiazole derivatives, bis-thiazole derivative **91a** demonstrated good activity against HCV virus of EC_50_ 0.56 μM, while 2*H*,*5H*-chromeno [4′,3′:4,5]thiopyrano[2,3-d][1,3]thiazole-5-carboxylic acids (**93**, **94**) were found to be the most active against influenza virus type A (H_3_N_2_, Perth strain), exhibiting high activity with EC_50_ = 0.6 ÷ 2.5 μg/mL and SI = 40.0 ÷ > 170.0. Spirothiazolidinone derivatives of imidazo[2,1-b] thiazole (**98d**) exhibited very good activity against Feline corona virus in CRFK cells, with an EC_50_ value of 4.8 μM and SI more than 20, being much more potent than ganciclovir. Pyrazolo [3.4,d] thiazole derivatives (**101a** and **101b**) were the most potent inhibitors of HIV-1 replication against HIV-1 IIIB with EC_50_ = 0.74 and 1.08 μg/mL respectively while benzothiazole-based thiazolidin-4-ones (**102a**–**102c**) displayed excellent activity against HI1RT with IC_50_ value lower than 5 nm.

The derivatives of {[5-arylidene-2-(4-fluorophenylimino)-4-oxothiazolidin-3-yl]methyl}benzoic acids and 2-thioxo-4-thiazolidinone analogues (**104c**, **104e**, and **105f**) with IC_50_ values of 1.6, 1.5, and 1.9 μΜ appeared to be good PTP1b inhibitors as well as 4-oxo-2-phenylthiazolidin-3-yl)methyl) benzoate (**109e**) with IC_50_ = 0.92 μΜ. On the other hand, thiazole-based quinoline salts (**113**) was found to be a good DPP4 inhibitor with an IC_50_ value of 0.38 nm, while coumarin–thiazoles (**114a**–**114c**) as well as 1,3-diarylpyrazol-rhodanine-3-hippuric acid derivatives were good inhibitors of ALR2 with IC_50_ values in range of 0.11–0.12 μΜ and 0.60 nM, respectively.

Among compounds with antimalarial activity, the derivative of nocathiacin **120** showed 170-fold and 20-fold higher activity against *P. falciparum* compared to thiostrepton and its derivatives, while N-((4-chlorophenyl)(phenyl)methyl)thiazol-2-amine (**125a**) could be useful in the treatment of cutaneous leishmaniasis, while the SI of compound **127b** was found to be 7-fold higher than the minimum recognized for a promising drug against trypanosomatid parasites, considering this compound as a promising antileishmanial agent.

Trisubstituted urea derivatives, especially compounds **129c**–**e** with IC_50_ values of 12, 9.9, and 9.7 nM respectively and SI 10200, 9960, and 11700 respectively appeared to be good agents against trypanosomiasis being more potent than reference drugs pentamidine and Melarsoprol (IC_50_ 2.8 and 4.0 μM). However, they lack *in vitro* results. 2,4-Disubstituted arylthiazole derivative 1**34α** showed higher selectivity, indicating a promising perspective for the design of novel trypanocidals, whereas among isothiochromeno[4a,4-d][1,3]thiazole derivatives compound **136a** displayed good activity against *Trypanosoma brucei* with an IC_50_ range of 1.55μM compared to reference drug Nifudimox (IC_50_ = 2.39 μM). 2-(pyridin-2-yl)-1,3-thiazole derivatives **140c**, **140g**, **140i,** and **140j** with (CC_50_ 2.2 and 2.3 μM respectively against rypomastigots seem to be novel potent and selective antiparasitic agents and can be a starting point for the further development of new antiparasitic agents.

Among compounds with anti-inflammatory activity, pyrazolylthiazolecarboxylates **146p**, **146c**, and **146n** with inhibition ranging from 93.06 to 89.59% seem to be a promising scaffold for designing anti-inflammatory agents. Thiazole-bearing pyrazole derivatives **147e** and **147h** offered protection of inflammation induced by carrageenan after 3 h (87.75% and 92.85%, respectively) being good COX-2 inhibitors with inhibition values of 78.91%, while thiazolo [4′,5′:4,5] thieno [2,3-*d*] pyrimidine derivative **150** exhibited 96% inhibition of edema. On the other hand, benzimidazothiazole derivative **159a** (IC_50_ 4.040 nΜ and 4.520 nM respectively) was the most active COX-1/COX-2 inhibitor, being more than 10 times higher than celecoxib and 115 times more potent than naproxen.

It was observed that thiazolidinone derivatives of 1,3-thiazole **170a** and **171** exhibited very good antioxidant activity (62.11% and 60.93%, respectively) according to TBARS assay, while purine–pyrazole hybrids incorporating thiazole, thiazolidinone, or rhodanine moiety compounds **173a**, **173b**, and **174a**–**b** exhibited good DPPH free radical scavenging potential (IC_50_ from 4.89 to 14.43 μg/mL) higher than ascorbic acid with an IC_50_ of 15.34 μg/mL

It should be mentioned that among thiazole derivatives with CA inhibitory activity, 4-[(3-cyclohexyl-4-aryl-2,3-dihydro-1,3-thiazol-2-ylidene) amino]benzene-1-sulfonamide derivative **180a** inhibited hCA II isoform in the nanomolar range (Ki = 0.07 nM) compared to AZZ (Ki = 12 nM). On the other hand, benzo[d]thiazole-5- and 6-sulfonamide **184c** inhibited hCA VII with Ki = 0.8 nM, while compounds **189b** and **189c** were very good inhibitors against hCA XII isoform with KI values of 3.1 and 4.6 nM against hCA XII isoform being more potent than AAZ (Ki = 5.7 nM).

Finally, it was observed that **197b** (2-[2,4-dioxo-5-(thiazol-2-ylcarbamoylmethyl)-thiazolidin-3-yl]-N-(2-trifluoromethylphenyl) acetamide) **197b** and **197c** ((2,4-dioxo-5-(thiazol-2-ylcarbamoylmethylene)-thiazolidin-3-yl)acetic acid ethyl ester) (Figure 73) demonstrated very good anticonvulsant potential in PTZ and MES assays.

The general observation is that hybrid derivatives of thiazole with different heterocyclic rings such as piperazine, pyridine, thiophen, imidazole, triazine, coumarin etc. as well as hydrazonyl thiazole derivatives are responsible for activities discussed in this review, while for PTP1b inhibition, thiazolidinone and heteroarylidene 1,3-thiazolidin-4—one derivatives are promising scaffolds.
